# Indications of Cannabinoids for the Palliation of Cancer-Associated Symptoms: A Systematic Review and Meta-Analysis

**DOI:** 10.1007/s11912-025-01695-x

**Published:** 2025-08-01

**Authors:** Ioana Creangă-Murariu, Ioana-Irina Rezuș, Roshanak Karami, Anett Rancz, Ádám Zolcsák, Marie Anne Engh, Mahmoud Obeidat, Bogdan-Ionel Tamba, Péter Hegyi, Stefania Bunduc

**Affiliations:** 1 Centre for Translational Medicine, Semmelweis University, Baross Utca 22, Budapest, 1082 Hungary; 2 Advanced Center for Research and Development in Experimental Medicine (CEMEX), “Grigore T. Popa” Medicine and Pharmacy University, Iași, Romania; 3 Medical Oncology-Radiotherapy Department, Grigore T. Popa University of Medicine and Pharmacy, Iași, Romania; 4 Department of Radiology, ” Grigore T. Popa” University of Medicine and Pharmacy, Iași, Romania; 5 Pharmacy Faculty, Semmelweis University, Budapest, Hungary; 6 Department of Biophysics and Radiation Biology, Semmelweis University, Budapest, Hungary; 7 Carol Davila University of Medicine and Pharmacy, Bucharest, Romania; 8 Digestive Disease and Liver Transplant Center, Fundeni Clinical Institute, Bucharest, Romania; 9 Institute for Translational Medicine, Medical School, University of Pécs, Pécs, Hungary; 10 Institute of Pancreatic Diseases, Semmelweis University, Budapest, Hungary; 11 Translational Pancreatology Research Group, Interdisciplinary Centre of Excellence for Research Development and Innovation, University of Szeged, Szeged, Hungary

**Keywords:** Meta-analysis, THC, CBD, Marijuana, Palliative care, Cancer

## Abstract

**Purpose of the Review:**

As cancer survival rates are increasing, alternative treatments to improve quality of life, such as cannabinoids, are gaining attention. Although cannabinoids are widely used to manage cancer-related symptoms, clear guidelines are lacking. This systematic review and meta-analysis assessed the safety and efficacy of cannabinoids in the management of symptoms among cancer patients. The study protocol was registered on PROSPERO (CRD42023479375). A systematic search was conducted using three main databases (PubMed, Embase, and CENTRAL) on 4 November 2023. We included interventional and observational studies that evaluated cannabinoids for symptom management in cancer patients compared to standard care, placebo, or baseline values. Pooled mean differences (MD), proportions and odds ratios (OR), and the 95% confidence intervals (CI) were calculated with a random-effects model.

**Recent Findings:**

Overall, 98 articles were eligible. Cannabinoids reduced pain (MRAW: -1.22, CI: -1.92; -0.52) and anxiety (MRAW: -1.30, CI: -2.22; -0.39) as compared to baseline values. Appetite (MRAW: -1.88, CI: -6.23; 2.46), chemotherapy-induced nausea and vomiting (OR: 2.18, CI: 0.79; 6.00), as well as insomnia (MD: -1.08, CI: -2.48; 0.33) presented with a tendency toward improvement. Cannabinoids do not influence constipation, depression, fatigue, mobility or overall quality of life. In terms of safety issues, THC-predominant formulations increase the risks of psychiatric (OR: 10.62, CI: 1.35; 83.57), neurological (OR:2.24, CI: 1.15; 4.35), and gastrointestinal (OR:2.69, CI:0.73;9.90) side effects. The risk of bias of articles included varied from some concerns to high.

**Summary:**

Cannabinoids may be beneficial for the treatment of cancer-related pain and anxiety; however, their use carries a significant risk of adverse effects, particularly psychiatric complications. Careful patient selection is essential when considering cannabinoid-based treatments.

**Supplementary Information:**

The online version contains supplementary material available at 10.1007/s11912-025-01695-x.

## Introduction

The global cancer incidence is projected to increase by nearly 30% until 2040, becoming the most prevalent disease worldwide [1]. However, cancer-associated mortality is on a downward trend, and survival rates continue to improve [2]. As a result, ensuring an optimal quality of life has become a central focus in the care of cancer patients.

The term"cannabinoids"refers to all agonists and antagonists of cannabinoid receptors, encompassing phytocannabinoids derived from the *Cannabis sativa* plant and synthetic cannabinoids. The best-known active compounds are tetrahydrocannabinol (THC) and cannabidiol (CBD) [3]. Cannabinoids have a long-standing history in medical practice, demonstrating benefits for degenerative, inflammatory, and neurological conditions [4]. Previous studies have reported potential benefits in alleviating chemotherapy-induced nausea and vomiting (CINV), insomnia, and pain in cancer patients [5]. Notably, 20% to 40% of cancer patients report using cannabinoids at least once to manage cancer-related symptoms [6–8].

The medicinal use of cannabinoids has been legalised in a few countries, such as the Netherlands, Canada, and certain regions of the United States, while others have adopted decriminalised policies. However, the majority of cancer patients still obtain cannabinoids through illicit means [9]. Although cannabinoids have demonstrated efficacy in managing cancer-related symptoms, including analgesia [10], cachexia [11], or chemotherapy-induced nausea and vomiting [12], clear recommendations are lacking in oncology guidelines.

Given the increasing popularity and use of cannabinoids among cancer patients, coupled with a growing body of research, yet limited conclusive evidence of their benefits, we conducted a systematic review and meta-analysis to address this issue. Our objective was to assess the safety and efficacy of various cannabinoid treatments for the management of cancer-associated symptoms in this patient population.

## Methods

We report our systematic review and meta-analysis based on the recommendations of the PRISMA 2020 guideline (Supplementary Table 1S) [13]; our study was conducted according to the Cochrane Handbook [14]. We fully adhered to the study protocol, which was initially registered on PROSPERO (CRD42023479375) [15].

### Eligibility Criteria

All available literature on the subject, including interventional and observational studies, was eligible for assessment irrespective of publication date and language. We included studies reporting on (P—population) cancer patients (regardless of age, sex, localization, histology, and stage) who were administered (I—intervention) cannabinoids (regardless of type, ratio of active molecules, mode of administration, form, and dosage) for the management of cancer-associated symptoms as compared to (C—control) standard of care, placebo, no cannabinoid group (for two-arm studies) or baseline values (in one-arm studies). Our (O) outcomes of interest were efficacy in the cancer-associated symptom control (such as pain, nausea, vomiting, insomnia, anxiety, depression, anorexia, mobility, and overall quality of life) and safety outcomes (adverse reactions such as neurologic, psychiatric, cardiovascular, gastrointestinal, pulmonary, hematologic or neoplasm progression). In terms of study design (S), both randomised controlled trials (RCTs), observational studies, and registered ongoing trials were eligible. We excluded conference abstracts, case reports, and case series.

### Information Sources

Our systematic search was conducted on 4 November 2023, in three major databases: MEDLINE (via PubMed), Embase, and CENTRAL (The Cochrane Central Register of Controlled Trials), without any filters or restrictions. The reference lists of all included articles were further checked using *citationchaser* (Version 2.0, Stockholm Environment Institute, Sweden)[16] on 19 November, 2023 to identify eligible articles. The search key included terms and synonyms for “cancer” and “cannabinoids”, as seen in Supplementary Table 7*.*

### Selection Process

The selection was performed by three independent review authors (IC-M, IIR, and RK). All references were imported in *Endnote 20* (Clarivate, 2013) for the removal of duplicate articles, followed by title-abstract selection using *Rayyan* (Version 1.0, Qatar Computing Research Institute (QCRI), Qatar) [17] and full-text selection. Cohen’s kappa coefficient (k) was calculated to measure inter-rate reliability after each selection step. Conflicts were resolved by a fourth independent reviewer (AR).

### Data Collection Process

Three authors (IC-M, IIR, RK) independently collected data, with a fourth independent reviewer (AR) resolving disagreements. Using a standardized form, we extracted the following data from eligible articles: title, first author, year of publication, Digital Object Identifier (DOI), country, number of centres involved, study design, study duration, inclusion/exclusion criteria, patient demographics, cancer localization, stage, previous cannabinoid use, background treatment for symptom control, number of patients enrolled in intervention/control, type of cannabinoid, concentration, form, dosage/24 h, total days of exposure, outcomes and outcome assessment tools. For continuous outcomes, we extracted sample size, mean and standard deviation (SD) or median, and interquartile ranges (IQR). For dichotomic outcomes, odds ratios (ORs) and 95% confidence intervals (CI) were extracted if reported. Otherwise, they were calculated based on the total number of patients with the event of interest from the intervention and control groups and the total number of patients in each group. If data were available only in figures and graphs, we used the *WebPlotDigitizer* tool (Version 4.6, Automeris, USA) to extract them. If data were incompletely reported, we contacted the corresponding authors and requested additional information necessary for our analysis. Articles where data were not poolable for meta-analysis were included in the systematic review. Outcomes were usually measured using some type of validated questionnaire (e.g. European Organisation For Research And Treatment of Cancer- Core Quality of Life questionnaire (EORTC QLQ-C30), Numerical Rating Scale (NRS), Visual Analogue Scale (VAS), Edmonton Symptom Assessment Scale (ESAS), Functional Assessment of Cancer Therapy (FACT) etc.), where symptom intensity was reported by the patient and quantified on a rating scale.

### Study Risk of Bias Assessment

Two authors (IC-M and IIR) independently performed the risk of bias assessment, and a third investigator (AR) resolved disagreements. We used ROB2 tool [18] for RCTs and ROBINS-I for non-randomised studies of intervention [19]. The certainty of evidence level was evaluated using the Grading of Recommendations Assessment, Development and Evaluation (GRADE) approach [20] and the GRADEpro tool (Version 3.2, McMaster University, Canada).

### Synthesis Methods

As we assumed considerable between-study heterogeneity at all cases, a random-effects model was used to pool effect sizes in a frequentist framework. Odds ratio (OR) was used as effect size measure for binary outcomes. To calculate the the odds ratios and the pooled odds ratio, the total number of patients and those with the event of interest in each group separately was extracted from the studies. The difference between the mean (MD) used for the effect size measure for continous outcomes. However, when the scales were not convertable to each other, we used standardized mean difference (SMD). To calculate the study MDs and pooled MD, the sample size, the mean and the corresponding standard deviation (SD) was extracted or estimated from each study (in each group separately). We reported the results as the odds of event of interest in the cannabioid treated group versus the odds of event of interest in the control group, or the mean in the cannabioid treated group minus the mean in the control group. Results were considered statistically significant, if the pooled 95% confidence interval (CI) does not contain the null value. We summarized the findings related to meta-analysis on forest plots. Between-study heterogeneity was described by the between-study variance (π [2]) and the Higgins and Thompson’s I^2^ statistics too [21]. We reported directly the prediction interval only if the study number was large enough (e.g. 5) and not too heterogenious—to give a meaningful estimation. Small study publication bias was assessed by visual inspection of Funnel-plots and calculating Egger (for continous outcomes), Pustejovsky (for SMD) or Harbord (for dichotomous) test *p*-value [22]. Although, we kept in mind that the test has a limited diagnostic assessment below ~ 10 study. Potential outlier publications was explored using different influence measures and plots following the recommendation of Harrer et. Al [23]. We performed subgroup analysis based on THC/CBD content of drugs. All statistical analyses were calculated by R software using the meta5 package for basic meta-analysis calculations and plots, and dmetar6 package for additional influental analysis calculations and plots.

## Results

### Search and Selection

**Fig. 1:**
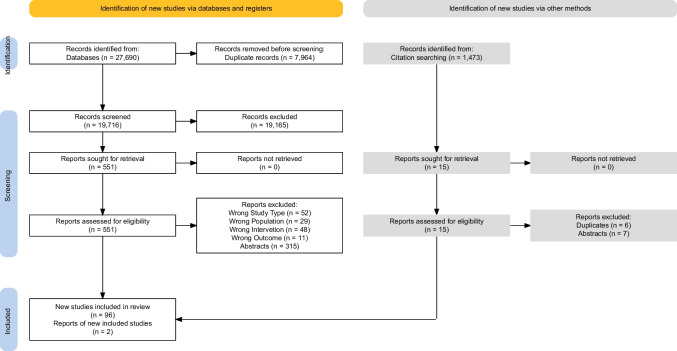
PRISMA flowchart of the article selection process

Altogether 27,690 studies were identified using our systematic literature search approach. In total, 98 articles were eligible, two of which were identified via *citationchaser.* A summary of the selection process is presented in Fig. 1**.**

### Characteristics of Studies Included

We included 56 RCTs [24–77], 13 non-randomised trials [78–90], and 29 observational studies [68, 91–115] (Table 1), where 21,397 patients were included. They included patients undergoing curative-intent or palliative oncological treatment who received different types of cannabinoid-based treatment (either THC-predominant, CBD-predominant, or a balanced ratio of THC and CBD (THC:CBD)) in a fixed dosage or titrated by the patient, via inhalation, oral, intramuscular, or topical route (Table 4, supplementary) and were compared either to baseline (pre-intervention) values for one-arm studies, or with placebo/no cannabinoid group, for two-arm studies. Data were limited for some outcomes evaluated, and heterogeneity was high. Nevertheless, the results across the studies included showed clear trends in favour of cannabinoid use.

**Table 1:** Basic characteristics of studies included

Nr	Author, Year	Study type	Cancer stage	Age (years)	Number of patients enrolled in intervention/control
1	Abrahamov et al., 1995 †	Non-RCT	Curable and Advanced	3–13 §	8 ‡
2	Ahmedzai et al., 1983 †	RCT	Advanced	58 ¶	34 ‡
3	Anderson et al., 2019 †	Prospective cohort	Curable and Advanced	59 ¶	1120 ‡
4	Aprikian et al., 2023 (a)	Prospective cohort	Curable and Advanced	57.6 ||	88 ‡
5	Aprikian et al., 2023 (b)	Prospective cohort	Curable and Advanced	57.6 ||	136 ‡
6	Aprikian et al., 2023 (c)	Prospective cohort	Curable and Advanced	57.6 ||	59 ‡
7	Aviram et al., 2020 (a)	Prospective cohort	Curable and Advanced	63 ¶	56 ‡
8	Aviram et al., 2020 (b)	Prospective cohort	Curable and Advanced	66 ¶	19 ‡
9	Aviram et al., 2020 (c)	Prospective cohort	Curable and Advanced	66 ¶	33 ‡
10	Aviram et al., 2022 †	Prospective cohort	Curable and Advanced	64 ¶	324 ‡
11	Awofisayo et al., 2021	Cross-sectional	Curable and Advanced	52 ||	14 ‡
12	Bar Sela et al., 2018 †	Case–control study	Advanced	63 ||	17 ‡
13	Bar-Sela et al., 2013 †	Prospective cohort	Curable and Advanced	NR	211 ‡
14	Brisbois et al., 2011	RCT	Advanced	67 ||	24/22
15	Chan et al., 1986 †	RCT	Curable and Advanced	11.8 ||	40 ‡
16	Chang A. et al., 1979 †	RCT	Curable	24 ¶	15/15
17	Chang A. et al., 1981 †	RCT	Curable	41 ¶	8/8
18	Chang et al., 2019	Retrospective cohort	Curable and Advanced	52 ¶	98/181
19	Clarke et al., 2022	Non-RCT	Advanced	55.9 ||	25 ‡
20	Cone et al., 1982 †	Non-RCT	Curable and Advanced	60.3 ||	52 ‡
21	Côté et al., 2016	RCT	Curable	63.5 ||	28/28
22	Crawford et al., 1986 †	RCT	Curable and Advanced	NR	32 ‡
23	Cronin et al., 1981 †	Non-RCT	Curable and Advanced	33 ¶	31 ‡
24	Cunningham et al., 1988 †	RCT	Curable and Advanced	42 ||	80 ‡
25	Dalzell et al., 1986 †	RCT	Curable and Advanced	NR	23 ‡
26	Davies et al., 1974 †	RCT	Advanced	50–70 §	12 ‡
27	Diasio et al., 1981 †	RCT	Curable and Advanced	47 ||	27 ‡
28	Dominika et al., 2023 †	Non-RCT	Curable and Advanced	62.3 ||	22/10
29	Donovan et al., 2019	Retrospective cohort	Curable and Advanced	49.2 ||	156/660
30	Duran et al., 2010 †	RCT	Advanced	50 ¶	7/9
31	Einhorn et al., 1981 †	RCT	Curable and Advanced	28 ¶	61 ‡
32	Einhorn et al., 1982 †	RCT	Curable and Advanced	28 ¶	100 ‡
33	Elder et al., 2015 †	Retrospective cohort	Curable and Advanced	13.9 ||	66 ‡
34	Eliott et al., 2016 †	Non-RCT	Curable	64 ||	16 ‡
35	Fallon et al., 2017	RCT	Advanced	60 ||	200/199
36	Frytak et al., 1979	RCT	Curable and Advanced	NR	38/37
37	Gerhartz et al., 1983 †	RCT	Curable and Advanced	NR	11 ‡
38	Good et al., 2020 (a)	Non-RCT	Advanced	57.7 ||	21 ‡
39	Good et al., 2020 (b)	Non-RCT	Advanced	57.7 ||	21 ‡
40	Grimison et al., 2020 †	RCT	Curable and Advanced	55 ||	40 ‡
41	Gulbransen et al., 2020 †	Prospective cohort	Curable and Advanced	NR	24 ‡
42	Hardy et al., 2022	RCT	Advanced	63.6 ||	70/72
43	Heim et al., 1982 †	RCT	Advanced	19–66 §	20 ‡
44	Heim et al., 1984 †	RCT	Advanced	49 ||	57 ‡
45	Herman et al., 1979 †	RCT	Curable and Advanced	33 ||	152 ‡
46	Hutcheon et al., 1988	RCT	Curable and Advanced	50.4 ||	27 ‡
47	Jatoi et al., 2002 †	RCT	Advanced	67 ||	152/159
48	Johansson et al., 1982 †	RCT	Curable and Advanced	18–70 §	27 ‡
49	Johnson et al. 2013 (a) †	RCT	Advanced	57.7 ||	39 ‡
50	Johnson et al. 2013 (b) †	RCT	Advanced	58.6 ||	4 ‡
51	Johnson et al., 2010 (a)	RCT	Advanced	59.4 ||	60/59
52	Johnson et al., 2010 (b)	RCT	Advanced	61.3 ||	58/59
53	Jones et al., 1982 †	RCT	Curable and Advanced	NR	54 ‡
54	Joss et al., 1982 †	Non-RCT	Curable and Advanced	49 ||	23 ‡
55	Kasvis et al., 2022 †	Prospective cohort	Curable and Advanced	57.6 ||	358 ‡
56	Lane et al., 1991 †	RCT	Curable and Advanced	47 ||	21 ‡
57	Laszlo et al., 1961 †	Non-RCT	Curable and Advanced	18–70 §	35 ‡
58	Lee et al., 2023 †	Cross-sectional	Curable and Advanced	61.1 ||	1464 ‡
59	Levitt et al., 1982 †	RCT	Advanced	17–73 §	57 ‡
60	Lichtman et al., 2018	RCT	Advanced	59.2 ||	199/198
61	Lucas et al., 1980 †	Non-RCT	Curable and Advanced	NR	57 ‡
62	Lucraft et al., 1982 †	RCT	Curable and Advanced	65 ||	29/14
63	Lynch et al., 2014	RCT	Curable and Advanced	58 ||	9/9
64	Macari et al., 2020 †	Cross-sectional	Curable and Advanced	> 18	46/142
65	Maida et al., 2008 †	Prospective cohort	Advanced	67 ||	47/65
66	Mccabe et al., 1988 †	RCT	Curable and Advanced	48 ||	36 ‡
67	McClure et al., 2023 †	Cross-sectional	Curable and Advanced	> 18	1036 ‡
68	Meghani et al., 2021 †	Prospective cohort	Curable and Advanced	60.9 ||	136 ‡
69	Meiri et al., 2007	RCT	Curable and Advanced	61.6 ||	17/14
70	Nathan et al., 2023	Retrospective cohort	Curable and Advanced	NR	83 ‡
71	Neiderle et al., 1986 †	RCT	Curable and Advanced	25 ||	20 ‡
72	Neidhart et al., 1981 †	RCT	Curable and Advanced	41 ||	37 ‡
73	Nelson et al., 1994 †	RCT	Advanced	64 ||	19 ‡
74	Nielsen et al., 2022 †	Cross-sectional	Curable and Advanced	all ages	2775 ‡
75	Niiranen et al., 1985 †	RCT	Curable and Advanced	61 ||	32 ‡
76	Niiranen et al., 1987 †	RCT	Curable and Advanced	> 18	40 ‡
77	Orr et al., 1980 †	RCT	Curable and Advanced	46 ||	79 ‡
78	Pasawarat et al., 2020 †	Retrospective cohort	Curable and Advanced	57 ||	137/95
79	Pitchard et al., 2019	Retrospective cohort	Advanced	48 ||	22/61
80	Polito et al., 2018 †	Retrospective cohort	Curable and Advanced	14 ||	110 ‡
81	Pomeroy et al., 1986 †	RCT	Advanced	42 ||	28 ‡
82	Portenoy et al., 2012	RCT	Active	59 ||	91/91
83	Saadeh et al., 2018 †	Cross-sectional	Curable and Advanced	61 ||	175 ‡
84	Sallan et al., 1975 †	RCT	Curable and Advanced	29.5 ||	20 ‡
85	Scheidler et al., 1984 †	RCT	Curable and Advanced	18–70 §	20 ‡
86	Schleider et al., 2018	Prospective cohort	Curable and Advanced	59.5 ||	2923 ‡
87	Schloss et al., 2021	RCT	Advanced	53.3 ||	88 ‡
88	Stambaugh et al., 1984 †	RCT	Curable and Advanced	NR	20/1
89	Strasser et al., 2006 (a)	RCT	Advanced	61 ||	95/48
90	Strasser et al., 2006 (b)	RCT	Advanced	60 ||	100/48
91	Sukpiriyagul et al., 2023	RCT	Curable and Advanced	54.4 ||	30/30
92	Sweet et al., 1981 †	Non-RCT	Curable and Advanced	51.5 ||	25 ‡
93	Turcott et al., 2018	RCT	Curable and Advanced	61.1 ||	14/19
94	Underleider et al., 1982 †	RCT	Curable and Advanced	47 ||	214 ‡
95	Underleider et al., 1985 †	RCT	Curable and Advanced	18–82 §	139 ‡
96	Wada et al., 1982 †	RCT	Curable and Advanced	57 ||	114 ‡
97	Waissengrin et al., 2015 †	Retrospective case–control	Curable and Advanced	57 ¶	279 ‡
98	Waissengrin et al., 2021 †	Retrospective cohort	Curable and Advanced	62.5 ||	246/265
99	Welsh et al., 1983 †	Non-RCT	Curable and Advanced	NR	15 ‡
100	Wongkongdech et al., 2022 (a) †	Cross-sectional	Advanced	56.2 ||	40/80
101	Wongkongdech et al., 2022 (b) †	Cross-sectional	Advanced	56.2 ||	40/80
102	Wongkongdech et al., 2022 (c) †	Cross-sectional	Advanced	56.2 ||	40/80
103	Zaki et al., 2017 †	Cross-sectional	Curable and Advanced	all age groups	2573 ‡
104	Zhang et al., 2018	Prospective cohort	Advanced	62.3 ||	74/74
105	Zutt et al., 2006 †	Non-RCT	Advanced	59 ||	7 ‡
106	Zylla et al., 2021	RCT	Advanced	57 ||	15/15

^†^ study included only in the systematic review

^‡^ studies without a comparator

^¶^ median

|| mean

^§^ range

*NR* not reported, *RCT* randomised controlled trial

- Cannabinoids reduce pain and anxiety regardless of THC/CBD content

**Fig. 2:**
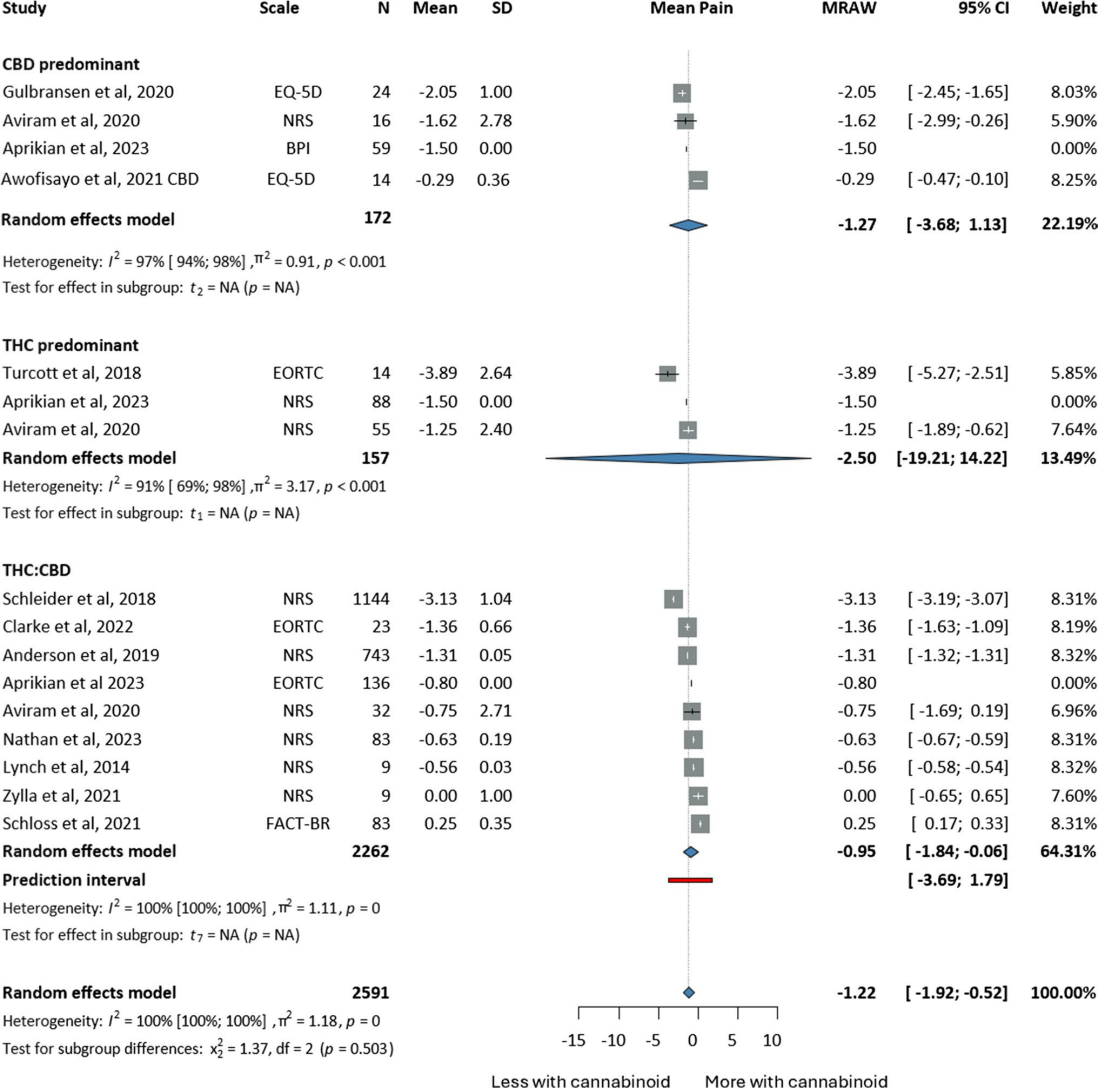
Forrest plots on the improvement of pain of cancer patients undergoing cannabinoid treatment; annotations: Tetrahydrocannabinol (THC), Cannabidiol (CBD), Sample Size (N), Standard Deviation (SD), Confidence Interval (CI), Raw or untransformed mean (MRAW), European Organisation For Research And Treatment of Cancer- Core Quality of Life questionnaire (EORTCQLQ30), Numerical Rating Scale (NRS), Brief Pain Inventory (BPI), Functional Assessment of Cancer Therapy (FACT), EuroQol (EQ-5D), Edmonton Symptom Assessment Scale (ESAS)

The results on cannabinoid efficacy in pain management are summarised in Fig. 2 and include twelve studies [54, 68, 69, 73, 77, 79, 91–94, 100, 107], where 2,591 patients were analyzed. Subgroup analyses were performed based on the THC/CBD content of the drug. Pain was assessed in one-arm studies, where baseline values were compared with post-intervention values and measured using the NRS scale, where 0 indicated no pain and 10 meant worst pain. Pain significantly decreased in the intervention group, regardless of the THC/CBD (MRAW: −1.22, CI:−1.92–0.52, I^2^ = 100% (Fig. 2)). Patients who were administered THC-predominant drugs seemed to have better analgesia levels (MRAW: −2.50, CI:−19.2;14.22).

**Fig. 3:**
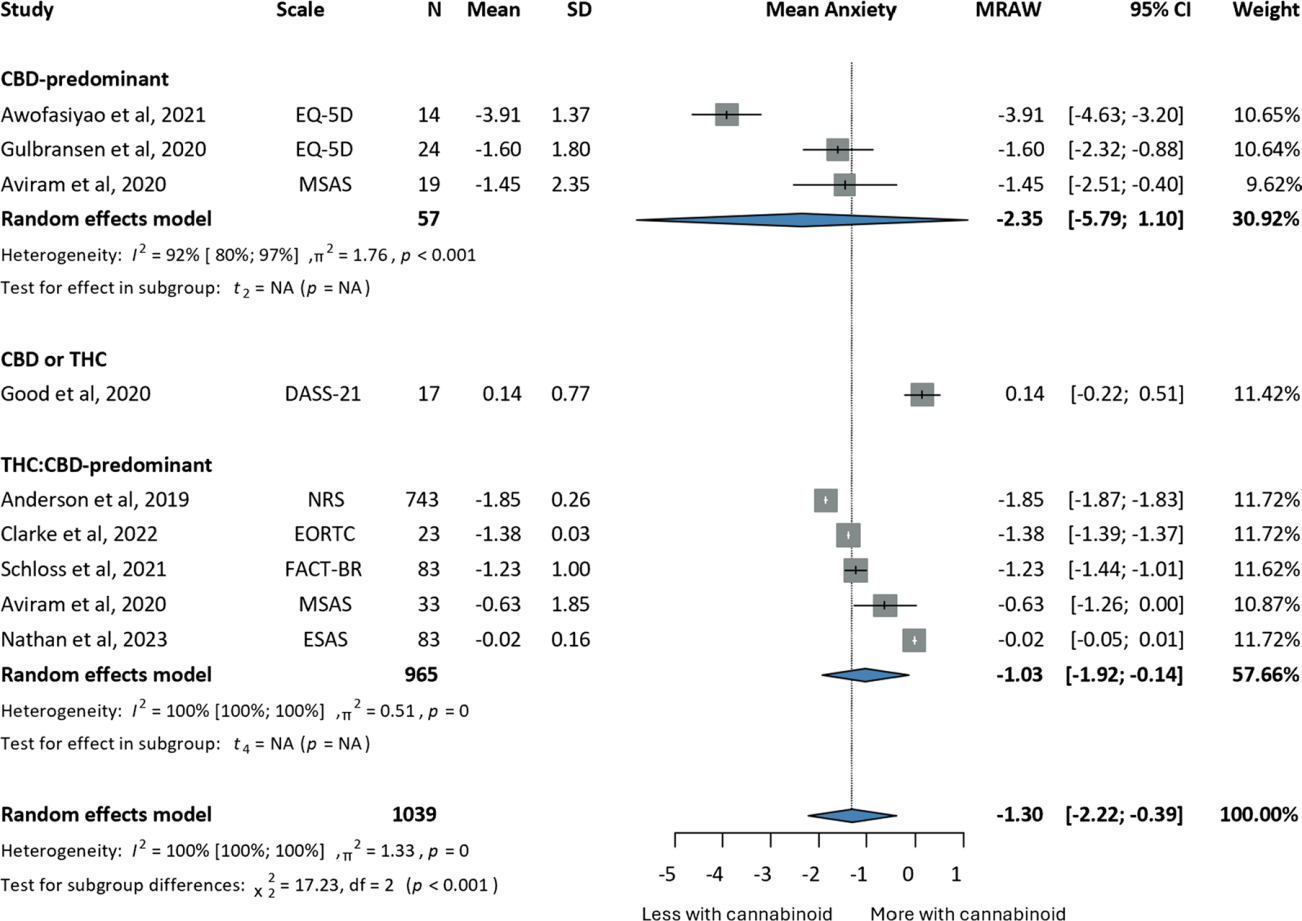
Forrest plots on the improvement of anxiety of cancer patients undergoing cannabinoid treatment; annotations: Tetrahydrocannabinol (THC), Cannabidiol (CBD), Sample Size (N), Standard Deviation (SD), Confidence Interval (CI), Raw or untransformed mean (MRAW), European Organisation For Research And Treatment of Cancer- Core Quality of Life questionnaire (EORTC-QLQ30), Numerical Rating Scale (NRS), Edmonton Symptom Assessment Scale (ESAS), Functional Assessment of Cancer Therapy (FACT), Pittsburgh Sleep Quality Index (PSQI) Memorial Symptom Assessment Scale Scale (MSAS), Depression, Anxiety and Stress Scale – 21 (DASS-21)

Baseline and post-intervention anxiety levels were compared to 1,039 patients from one-arm studies. Anxiety decreased to a significantly greater extent in the intervention versus the control group (MRAW: −1.30, CI:−2.22;−0.39, I^2^ = 100% (Fig. 3)) [69, 79, 84, 91, 93, 94, 100, 107]. CBD-predominant users presented with the highest decrease in anxiety (MRAW: −2.35, CI:−5.79;1.10).

b) Cannabinoids tend to improve appetite, nausea and insomnia

**Fig. 4:**
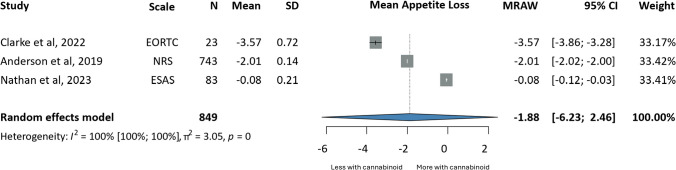
Forrest plots on the effects of cannabinoids on appetite; annotations: Sample Size (N), Standard Deviation (SD), Confidence Interval (CI), Raw or Untransformed Mean (MRAW), European Organisation For Research And Treatment of Cancer- Core Quality of Life questionnaire (EORTC-QLQ30), Numerical Rating Scale (NRS), Edmonton Symptom Assessment Scale (ESAS)

**Fig. 5:**
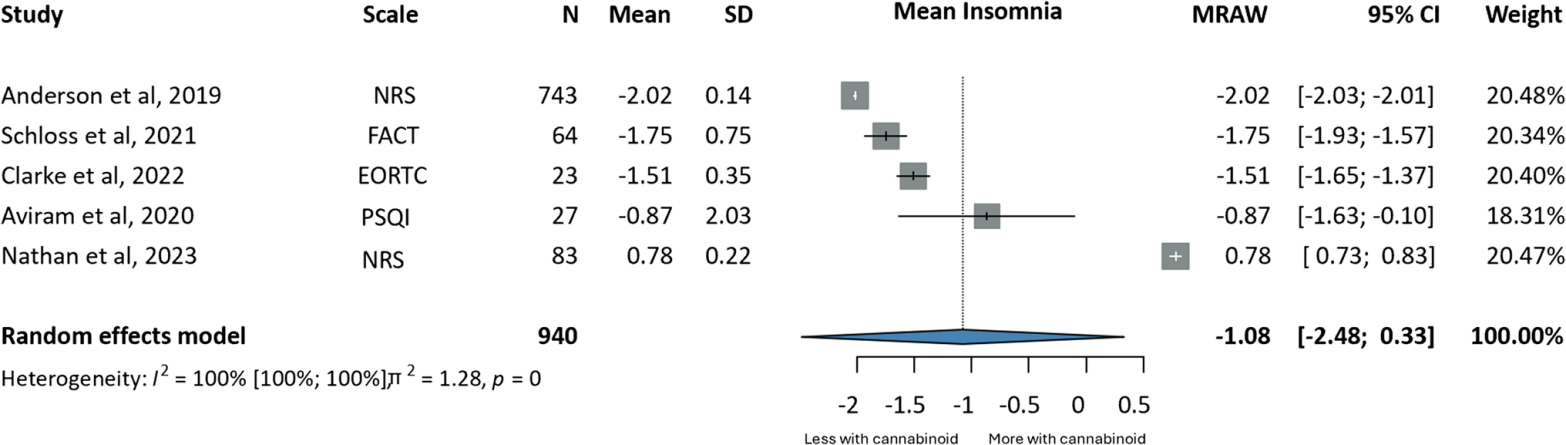
Forrest plots on the effects of cannabinoids on insomnia; annotations: Sample Size (N), Standard Deviation (SD), Confidence Interval (CI), Raw or Untransformed Mean (MRAW), European Organisation For Research And Treatment of Cancer- Core Quality of Life questionnaire (EORTC-QLQ30), Numerical Rating Scale (NRS), Functional Assessment of Cancer Therapy (FACT), Pittsburgh Sleep Quality Index (PSQI)

**Fig. 6:**
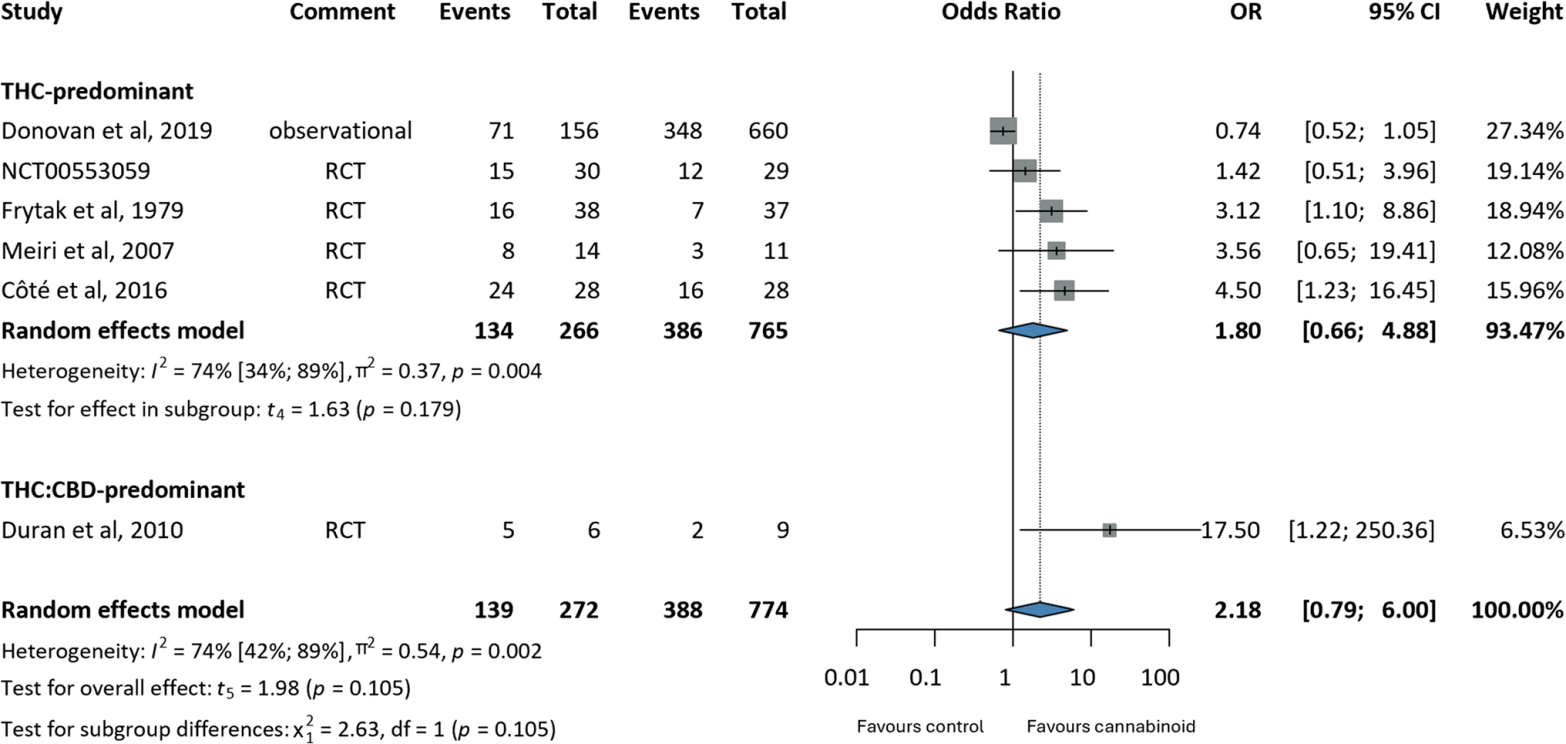
Forrest plots on the effects of cannabinoids on chemotherapy-associated nausea; annotations: Confidence Interval (CI), Odds Ratio (OR), Randomised Controlled Trial (RCT)

Appetite loss (MRAW: −1.88, CI:−6.23;2.46) [79, 91, 107] and insomnia (MD: 1.08, CI:−2.48;0.33) [69, 79, 91, 93, 107] were improved to a greater extent in the intervention group, compared to baseline values; however, differences were not statistically significant. Similar results were obtained for complete response to chemotherapy-associated nausea and vomiting (OR 2.18, CI:0.79;6.00) (i.e. no vomiting and no rescue medications used) [29, 34, 38, 56, 98], where patients in the intervention group were compared to placebo (Figs. 4, 5 and 6).

c) No effect of cannabinoids on constipation, depression, fatigue, mobility, or overall quality of life. Nabiximols does not improve pain and insomnia

In comparison to placebo, we did not find any clinically relevant effects of cannabinoids on constipation (MD: −0.19, CI:−0.68;0.30) [37, 41, 47, 52], depression (MD: 0.60, CI:−0.65;1.86) [41, 115], fatigue (MD: 0.30, CI:−1.24;1.83) 41, 47, 73, 115], mobility (MD: −0.05, CI:−0.42;0.32) [41, 47, 54, 73, 115], or overall quality of life (MD: 0.16, CI:−0.02;0.35) [25, 41, 47, 71, 73] (Supplementary Figs. 3S, 5, 6, 10, 19). Moreover, we performed a separate analysis on Nabiximols, which failed to reduce pain (MD: −0.25, CI:−0.5;−0.01) [37, 41, 52, 54, 65] or insomnia (MD: −0.2, CI:−0.35;−0.05)[37, 41, 47, 52, 73] as compared to placebo, as seen in Figs. 8, and 17 in Supplementary Material.

d) Side effects of cannabinoids: psychiatric, neurological and gastrointestinal symptoms

Side effects *(*Table 2*)* were assessed in two-arm studies, where cannabinoid use almost doubled the risk of gastrointestinal adverse effects (OR: 1.88, CI: 1.14;3.11) [34, 37, 38, 41, 47, 50, 52, 54, 56, 71, 72]. The most frequently reported were diarrhea, constipation, abdominal pain, nausea, vomiting, dry mouth, or stomatitis. Neurological side effects included confusion, difficulty concentrating, coordination problems, and dizziness and were twice as common in the cannabinoid group (OR: 2.06, CI:1.15;3.68)[34, 37, 38, 44, 47, 50, 52, 54, 56, 58, 65, 71, 72], regardless of the THC/CBD content. Notably, psychiatric side effects (hallucinations, delusion, paranoia, psychosis, nightmares, anxiety, and mood swings) were three times more prevalent in the intervention group (OR: 3.24, CI:1.48;7.1) [34, 38, 41, 50, 54, 58, 65, 72], and even ten times higher for THC-predominant drugs (OR: 10.62, CI:1.35;83.57). Overall, the odds of discontinuation of cannabinoid treatment due to side effects were 1.53 (CI:0.99;2.35), with twofold higher values for THC-predominant products (OR: 3.01, CI:0.32;27.89) [34, 37, 38, 41, 47, 50, 52, 56, 65, 71]. This effect was approximately twice as large for THC-predominant products, with an OR of 3.01 (CI: 0.32–27.89).

**Table 2:** Pooled results for cannabinoid-induced adverse events

Adverse event	Intervention	OR (95%CI)	I^2^ (95%CI)	Articles included (n)
Neurologic	All types	2.26 (1.33;3.84)	49% (11%,71%)	17
Neurologic	THC-predominant	2.24 (1.15;4.35)	37% (0%, 71%)	9
Neurologic	Balanced THC:CBD	2.12 (1.06;4.24)	47% (0%,73%)	12
Psychiatric	All types	4.32 (1.37;13.66)	64% (27%,83%)	9
Psychiatric	THC-predominant	10.62 (1.35;83.57)	18% (0%,87%)	4
Psychiatric	Balanced THC:CBD	2.58 (0.20;33.12)	65% (0%,88%)	4
Pain	All types	0.89 (0.45;1.62)	0% (0%,85%)	4
Hematological	All types	1.07 (0.78;1.46)	0% (0%,75%)	6
Asthenia	All types	1.47 (0.81;2.67)	12% (0%,53%)	9
Asthenia	THC-predominant	1.84 (1.56;2.18)	0% (0%,90%)	3
Asthenia	Balanced THC:CBD	1.63 (0.20;12.95)	54% (0%,83%)	5
Anorexia	All types	0.51 (0.08;3.32)	74% (36%,90%)	5
Dyspnea	All types	1.76 (0.81;3.79)	0% (0%,79%)	5
Cardiovascular	All types	1.03 (0.51;2.09)	0% (0%,60%)	11
Cardiovascular	THC-predominant	1.40 (0.34;5.80)	0% (0%,79%)	5
Cardiovascular	Balanced THC:CBD	0.95 (0.31;2.90)	8% (0%,77%)	6
Somnolence	All types	1.51 (0.95;2.40)	38% (0%,68%)	13
Somnolence	THC-predominant	0.73 (0.35;1.54)	1% (0%,79%)	5
Somnolence	Balanced THC:CBD	2.63 (1.77;3.89)	0% (0%,71%)	7
Gastrointestinal	All types	1.88 (1.14;3.11)	61% (32%−77%)	16
Gastrointestinal	THC-predominant	2.69 (0.73;9.90)	67% (13%,87%)	5
Gastrointestinal	Balanced THC:CBD	1.79 (0.92;3.49)	60% (20%,80%)	10
Neoplasm progression	Balanced THC:CBD	1.16 (0.70;1.92)	53% (0%,81%)	6
Stopped intervention	All types	1.61 (0.89;2.90)	37% (0%,68%)	12
Stopped intervention	THC-predominant	2.68 (0.37;19.56)	61% (0%,85%)	5
Stopped intervention	Balanced THC:CBD	1.32 (1.04;1.67)	0% (0%,65%)	9

Annotations: Tetrahydrocannabinol (THC), Cannabidiol (CBD), odds ratio (OR), Confidence Interval (CI). Corresponding forest plots are included in Supplementary Material, Figs. 33–60

### Risk of Bias Assessment

The results of the risk of bias assessment are presented in the *Supplementary Material.* For the RCTs, the risk of bias ranged from some concerns to high, and the randomization process, deviation from the intended interventions, missing outcome data, issues with outcome measurement, or selection of the reported results were the main bias sources. The results were similar in observational studies where the main bias sources were due to confounding factors, selection of participants, missing data, or selection of reported results.

### Publication Bias and Heterogeneity

Heterogeneity ranged between moderate and high (I^2^ 48%,100%) for pain, appetite, constipation, opioid intake, depression, anxiety, fatigue, mobility, CINV, with the exemption of insomnia and QoL in two-arm studies where I^2^ was zero. The risk of publication bias was high for anxiety, appetite improvement, insomnia, nausea, pain, QoL (Supplementary Tables 23, 24, 26, 28, 30, 32). The certainty of evidence assessed with the GRADE tool was very low to low for all outcomes, mainly due to population heterogeneity and lack of uniformity in intervention administration, as seen in Supplementary Tables 5 and 6.

## Discussion

We conducted a comprehensive systematic review and meta-analysis on the efficacy and safety of all types of cannabinoid treatment for cancer patients. Our study is the first to show clear, objective, and clinically significant benefits of cannabinoids in alleviating cancer-related pain (especially THC-predominant drugs) and anxiety (especially CBD-predominant drugs). Moreover, we showed that cannabinoids might improve appetite, CINV, and insomnia. On the other hand, our study draws attention to the safety profile of cannabinoids, emphasizing the risk for neurological, psychiatric, and gastrointestinal side effects.

Pain is among the most studied outcomes in the trials of cannabinoid treatment for cancer patients, and previous results failed to clarify their efficacy. Several meta-analyses reported on pain but included only RCTs where Nabiximols alone was evaluated and failed to show any clinically relevant improvement [116–118]. However, other meta-analyses did not evaluate other cannabinoids for cancer pain, nor did they include data from both interventional and observational studies. Our results bring comprehensive evidence for cannabinoid efficacy for cancer pain. Cancer pain is caused by different etiologies and underlying mechanisms, with distinct entities such as nociceptive, neuropathic, bone, somatic, or visceral, often in need of different treatment approaches [119], but it is impossible to subgroup accordingly based on the articles analyzed. Nevertheless, the population included patients at different stages of cancer, curable or metastatic, which greatly contributed to differences in pain or anxiety levels [120]. Another important point is the lack of standardization or good manufacturing practice (GMP) certification for several of the products used across the studies included. For pain analysis, only five trials used standardised, GMP-certified drugs [54, 69, 73, 79, 93, 100], whereas, in seven trials, the GMP certification was not mentioned [68, 77, 91–94, 107].

Anxiety is usually a secondary outcome in cannabinoid studies in cancer patients. The Multinational Association of Supportive Care in Cancer guidelines published in 2023 concluded that due to increased heterogeneity and ineffectiveness of the available data, no recommendation was possible for the use of cannabinoids for anxiety, depression, or insomnia [121]. Our results revealed a clinically significant reduction in anxiety levels in cannabinoid users. Although we are the first to show the beneficial effects of cannabinoids on the reduction of pain and anxiety in cancer patients, the level of evidence of our findings as per the GRADE evaluation was very low. This was mainly due to the high heterogeneity of the studies included, which differed in terms of population, intervention and design. Notably, only four out of eight articles on anxiety [69, 79, 84, 91, 93, 100], used standardised cannabinoids.

Interestingly, when pooling data from observational and interventional studies, we found that cannabinoids with balanced THC:CBD seemed to increase appetite. Other groups focused exclusively on RCTs, where cannabinoids were inefficient for appetite improvement [122], which is why oncology guidelines currently recommend against this indication [12]. In fact, Dronabinol, Nabilone, and a balanced ratio of THC:CBD products are mentioned as efficient for refractory nausea and vomiting in addition to the standard of care but with moderate quality of evidence [12]. It should be noted that the majority of studies were published in the 1980 s, before modern antiemetic prophylactic regimens and evidence-based guidelines became available. Also, these were mainly cross-over studies that did not report data before switching to the standard of care. Our results bring evidence for the benefits of cannabinoids on CINV, but without statistical significance, which is in line with an older comprehensive review on the topic [123]. Our results on insomnia are also similar to those of a previous systematic review—cannabinoids tend to reduce it; however, the differences between the groups are not statistically significant [124].

As for adverse events, when administering cannabinoids, proper dosing and titration are of utmost importance for naïve or chronic users [12]. The most prominent type we identified was psychiatric adverse events. Amidst THC-predominant users, a high proportion abandoned treatment due to the severity of the events. These results are consistent with data on psychiatric side effects in healthy adults who use THC-based drugs for recreational purpose [125]. However, CBD has the unique property of tapering down the psychotropic activity of THC by modulating different molecular pathways within the central nervous system (CNS), as emphasised by previous authors [126]. This is also supported by our results, where the addition of CBD to THC-predominant drugs resulted in a lower risk of psychiatric events. We also found important associations for confusion, dizziness, concentration, coordination problems, and somnolence, regardless of THC/CBD content. Cannabinoids exert their effects by binding the cannabinoid receptors 1 and 2, which have great densities within the CNS [127], which may explain the variety of their the adverse effects but also the improvement of symptoms processed in different areas of the brain, such as pain or nausea.

Our study lacked uniformity in outcome results, partly due to the lack of standardised dosage control. Patients were often advised to titrate the drug based on their individual needs, resulting in variability. This variability is compounded by the biphasic nature of cannabinoids, where their effects can be antagonistic depending on the dosage. For instance, lower doses of THC reduce pain, whereas higher doses can exacerbate pain and anxiety[65, 128]. In addition, heterogeneity was due to differences in THC/CBD ratios and routes of administration across trials, which included oral capsules, smoking, vaporization, oromucosal sprays, and intramuscular injections. To address this, we stratified analyses by THC/CBD content to explore variations in outcomes based on the type of cannabinoid. Our findings indicate that balanced THC:CBD ratios were most effective for insomnia and appetite, CBD-predominant products were more effective for anxiety, and THC-predominant products showed efficacy for pain and chemotherapy-induced nausea and vomiting (CINV). It is important to note, however, that cannabis plants contain more than 500 additional chemical compounds beyond THC and CBD, including phenols, alcohols, aldehydes, n-alkanes, alkaloids, flavonoids, terpenoids, wax esters, and steroids. These compounds contribute to the “entourage effect,” where the combined action of various components influences the overall therapeutic effects. The concentrations and proportions of these compounds vary across cannabis strains, further contributing to differences in health outcomes [129].

### Strengths and Limitations

Our study has a number of significant strengths. It is the most comprehensive and up-to-date systematic review on the usage of cannabinoids for cancer patients, incorporating data on a wide range of cannabinoid product types derived from both randomised trials and real-world evidence from observational studies. The analysis encompasses diverse highly relevant outcomes, addressing both efficacy and adverse effects. In addition, the inclusion of a large patient population in most analyses enabled clinically meaningful subgrouping. Furthermore, the study adheres to a rigorous methodology with transparent reporting, maintaining complete alignment with the pre-registered protocol.

However, the limitations of this work should also be emphasised. Although novel, our results rely on a low level of evidence. The main findings are based on data from real-world, observational trials and the included studies have a generally increased risk of bias. This raises the question of whether the observed benefits are truly objective or potentially influenced by the"placebo effect,"which is known to significantly impact outcomes in quality-of-life studies, although its underlying mechanisms remain not fully understood[130]. Moreover, there was considerable heterogeneity across most of our results, as discussed earlier. An important aspect is the substantial body of cannabinoid research from studies conducted in the 1980 s, primarily crossover studies. These studies often carry a high risk of bias and questionable result reporting, yet they form the basis of the current guideline recommendations.

### Implications for Practice and Research

The use of cannabinoids in the palliative care of cancer patients may be extended to include indications for pain and anxiety management [131, 132]. Prescribers should be aware of the safety profile, which implies a strict selection of patients, given the increased risk of psychiatric, neurological, or gastrointestinal side effects.

No recommendations on specific forms of administration or effective dosages can be made at this time. Further high-quality RCTs are needed to strengthen the evidence base to confirm the beneficial effects of cannabinoids in the treatment of pain and anxiety. In addition, these studies should evaluate their efficacy in addressing other symptoms such as appetite loss, insomnia, and CINV. Future research should focus on GMP-certified cannabinoid products to facilitate standardization of dosage regimens and ensure consistent clinical application [5].

## Conclusion

Cannabinoids may be effective in the treatment of cancer-associated pain and anxiety and may also provide benefits for appetite, chemotherapy-induced nausea and vomiting (CINV), and insomnia. However, their use is associated with significant psychiatric, neurological, and gastrointestinal side effects. These findings have substantial and immediate clinical implications, underscoring the necessity of updating guidelines to refine the indications for cannabinoid-based treatments of cancer patients.

## Key references

- Boland EG, Bennett MI, Allgar V, Boland JW. Cannabinoids for adult cancer-related pain: systematic review and meta-analysis. BMJ Support Palliat Care 2020; 10: 14–24.
  - Previous meta-analysis did not find any clinically relevant pain improvement with Nabiximols.
- Whiting PF, Wolff RF, Deshpande S, et al. Cannabinoids for medical use: A systematic review and meta-analysis. JAMA 2015; 313: 2456–73.
  - Previous meta-analysis did not find any clinically relevant pain improvement with Nabiximols.
- Mücke M, Weier M, Carter C, et al*.* Systematic review and meta-analysis of cannabinoids in palliative medicine. J Cachexia Sarcopenia Muscle 2018; 9: 220–34.
  - Previous meta-analysis did not find any clinically relevant pain improvement with Nabiximols.
- De Feo G, Case AA, Crawford GB, et al. Multinational association of supportive care in cancer (MASCC) guidelines: cannabis for psychological symptoms including insomnia, anxiety, and depression. Support Care Cancer 2023; 31: 176.
  - Previous meta-analysis which did not find any clinically relevant anxiety improvement with cannabinoids.

## Supplementary Information

Below is the link to the electronic supplementary material.

Supplementary file1 (DOCX 2319 KB)

## Data Availability

No datasets were generated or analysed during the current study.
