# Supplementary material for: Indications of Cannabinoids for the Palliation of Cancer-Associated Symptoms: A Systematic Review and Meta-Analysis
Source: Curr Oncol Rep. 2025 Aug 1;27(9):1080–96. doi: 10.1007/s11912-025-01695-x (PMC12450232; doi:10.1007/s11912-025-01695-x)
Supplement: Supplementary file 1 — Supplementary file1 (DOCX 2319 KB) [file 11912_2025_1695_MOESM1_ESM.docx]

**Indications of cannabinoids for the palliation of cancer-associated symptoms: a systematic review and meta-analysis**

**Authors**

Ioana Creangă-Murariu^1,2,3^, Ioana-Irina Rezuș^1,2,4^, Roshanak Karami^154^, Anett Rancz^1^, [Ádám Zolcsák](https://pubmed.ncbi.nlm.nih.gov/?sort=date&size=200&term=Zolcs%C3%A1k+%C3%81&cauthor_id=39154431)^6^, Marie Anne Engh^1^, Mahmoud Obeidat^1^, Bogdan-Ionel Tamba^2*^, Péter Hegyi^1,,9,10,11^* Stefania Bunduc ^1,7,8 *^

**Affiliations**

^1^ Centre for Translational Medicine, Semmelweis University, Budapest, Hungary

^2^ Advanced Center for Research and Development in Experimental Medicine (CEMEX), “Grigore T. Popa” Medicine and Pharmacy University Iasi, Romania

^3^ Regional Institute of Oncology, Iasi, Romania

^4^ Department of Radiology, ”Grigore T. Popa” University of Medicine and Pharmacy Iași, Romania

^5^ Pharmacy Faculty, Semmelweis University, Budapest
^6^ Department of Biophysics and Radiation Biology, Semmelweis University, Budapest, Hungary;

^7^ Carol Davila University of Medicine and Pharmacy, Bucharest, Romania

^8^ Digestive Disease and Liver Transplant Center, Fundeni Clinical Institute, Bucharest, Romania

^9^ Institute for Translational Medicine, Medical School, University of Pécs, Pécs, Hungary 
^10^ Institute of Pancreatic Diseases, Semmelweis University, Budapest, Hungary

^11^ Translational Pancreatology Research Group, Interdisciplinary Centre of Excellence for Research Development and Innovation, University of Szeged, Szeged, Hungary

| **Section and Topic** | **Item #** | **Checklist item** | **Location where item is reported** |
| --- | --- | --- | --- |
| **TITLE** | | |  |
| Title | 1 | Identify the report as a systematic review. | PAG. 1 |
| **ABSTRACT** | | |  |
| Abstract | 2 | See the PRISMA 2020 for Abstracts checklist. | PAG. 3 |
| **INTRODUCTION** | | |  |
| Rationale | 3 | Describe the rationale for the review in the context of existing knowledge. | PAG. 5 |
| Objectives | 4 | Provide an explicit statement of the objective(s) or question(s) the review addresses. | PAG. 5 |
| **METHODS** | | |  |
| Eligibility criteria | 5 | Specify the inclusion and exclusion criteria for the review and how studies were grouped for the syntheses. | PAG. 6 |
| Information sources | 6 | Specify all databases, registers, websites, organisations, reference lists and other sources searched or consulted to identify studies. Specify the date when each source was last searched or consulted. | PAG. 6 |
| Search strategy | 7 | Present the full search strategies for all databases, registers and websites, including any filters and limits used. | Supplementary |
| Selection process | 8 | Specify the methods used to decide whether a study met the inclusion criteria of the review, including how many reviewers screened each record and each report retrieved, whether they worked independently, and if applicable, details of automation tools used in the process. | PAG. 6 |
| Data collection process | 9 | Specify the methods used to collect data from reports, including how many reviewers collected data from each report, whether they worked independently, any processes for obtaining or confirming data from study investigators, and if applicable, details of automation tools used in the process. | PAG. 6 |
| Data items | 10a | List and define all outcomes for which data were sought. Specify whether all results that were compatible with each outcome domain in each study were sought (e.g. for all measures, time points, analyses), and if not, the methods used to decide which results to collect. | PAG. 6 |
|  | 10b | List and define all other variables for which data were sought (e.g. participant and intervention characteristics, funding sources). Describe any assumptions made about any missing or unclear information. | PAG. 6 |
| Study risk of bias assessment | 11 | Specify the methods used to assess risk of bias in the included studies, including details of the tool(s) used, how many reviewers assessed each study and whether they worked independently, and if applicable, details of automation tools used in the process. | PAG. 7 |
| Effect measures | 12 | Specify for each outcome the effect measure(s) (e.g. risk ratio, mean difference) used in the synthesis or presentation of results. | PAG. 7 |
| Synthesis methods | 13a | Describe the processes used to decide which studies were eligible for each synthesis (e.g. tabulating the study intervention characteristics and comparing against the planned groups for each synthesis (item #5)). | PAG. 6,7,8 |
|  | 13b | Describe any methods required to prepare the data for presentation or synthesis, such as handling of missing summary statistics, or data conversions. | PAG. 7 |
|  | 13c | Describe any methods used to tabulate or visually display results of individual studies and syntheses. | PAG. 7 |
|  | 13d | Describe any methods used to synthesize results and provide a rationale for the choice(s). If meta-analysis was performed, describe the model(s), method(s) to identify the presence and extent of statistical heterogeneity, and software package(s) used. | PAG. 7 |
|  | 13e | Describe any methods used to explore possible causes of heterogeneity among study results (e.g. subgroup analysis, meta-regression). | PAG. 7 |
|  | 13f | Describe any sensitivity analyses conducted to assess robustness of the synthesized results. | N/A |
| Reporting bias assessment | 14 | Describe any methods used to assess risk of bias due to missing results in a synthesis (arising from reporting biases). | PAG. 7 |
| Certainty assessment | 15 | Describe any methods used to assess certainty (or confidence) in the body of evidence for an outcome. | N/A |
| **RESULTS** | | |  |
| Study selection | 16a | Describe the results of the search and selection process, from the number of records identified in the search to the number of studies included in the review, ideally using a flow diagram. | PAG. 7 |
|  | 16b | Cite studies that might appear to meet the inclusion criteria, but which were excluded, and explain why they were excluded. | N/A |
| Study characteristics | 17 | Cite each included study and present its characteristics. | PAG. 8 |
| Risk of bias in studies | 18 | Present assessments of risk of bias for each included study. | Supplementary |
| Results of individual studies | 19 | For all outcomes, present, for each study: (a) summary statistics for each group (where appropriate) and (b) an effect estimate and its precision (e.g. confidence/credible interval), ideally using structured tables or plots. | PAG. 8-12 |
| Results of syntheses | 20a | For each synthesis, briefly summarise the characteristics and risk of bias among contributing studies. | N/A |
|  | 20b | Present results of all statistical syntheses conducted. If meta-analysis was done, present for each the summary estimate and its precision (e.g. confidence/credible interval) and measures of statistical heterogeneity. If comparing groups, describe the direction of the effect. | PAG. 8-12 Supplementary |
|  | 20c | Present results of all investigations of possible causes of heterogeneity among study results. | PAG. 12-15 |
|  | 20d | Present results of all sensitivity analyses conducted to assess the robustness of the synthesized results. | N/A |
| Reporting biases | 21 | Present assessments of risk of bias due to missing results (arising from reporting biases) for each synthesis assessed. | Supplementary |
| Certainty of evidence | 22 | Present assessments of certainty (or confidence) in the body of evidence for each outcome assessed. | N/A |
| **DISCUSSION** | | |  |
| Discussion | 23a | Provide a general interpretation of the results in the context of other evidence. | PAG. 13-14 |
|  | 23b | Discuss any limitations of the evidence included in the review. | PAG. 15 |
|  | 23c | Discuss any limitations of the review processes used. | PAG. 15 |
|  | 23d | Discuss implications of the results for practice, policy, and future research. | PAG. 15 |
| **OTHER INFORMATION** | | |  |
| Registration and protocol | 24a | Provide registration information for the review, including register name and registration number, or state that the review was not registered. | PAG. 5 |
|  | 24b | Indicate where the review protocol can be accessed, or state that a protocol was not prepared. | PAG. 5 |
|  | 24c | Describe and explain any amendments to information provided at registration or in the protocol. | PAG. 5 |
| Support | 25 | Describe sources of financial or non-financial support for the review, and the role of the funders or sponsors in the review. | PAG. 2 |
| Competing interests | 26 | Declare any competing interests of review authors. | PAG. 2 |
| Availability of data, code and other materials | 27 | Report which of the following are publicly available and where they can be found: template data collection forms; data extracted from included studies; data used for all analyses; analytic code; any other materials used in the review. | PAG. 5-7 |

**Table 1S. PRISMA Checklist**

1. **Two-arm studies (intervention vs placebo/standard of care/no cannabinoid group)**
2. ***Appetite improvement***


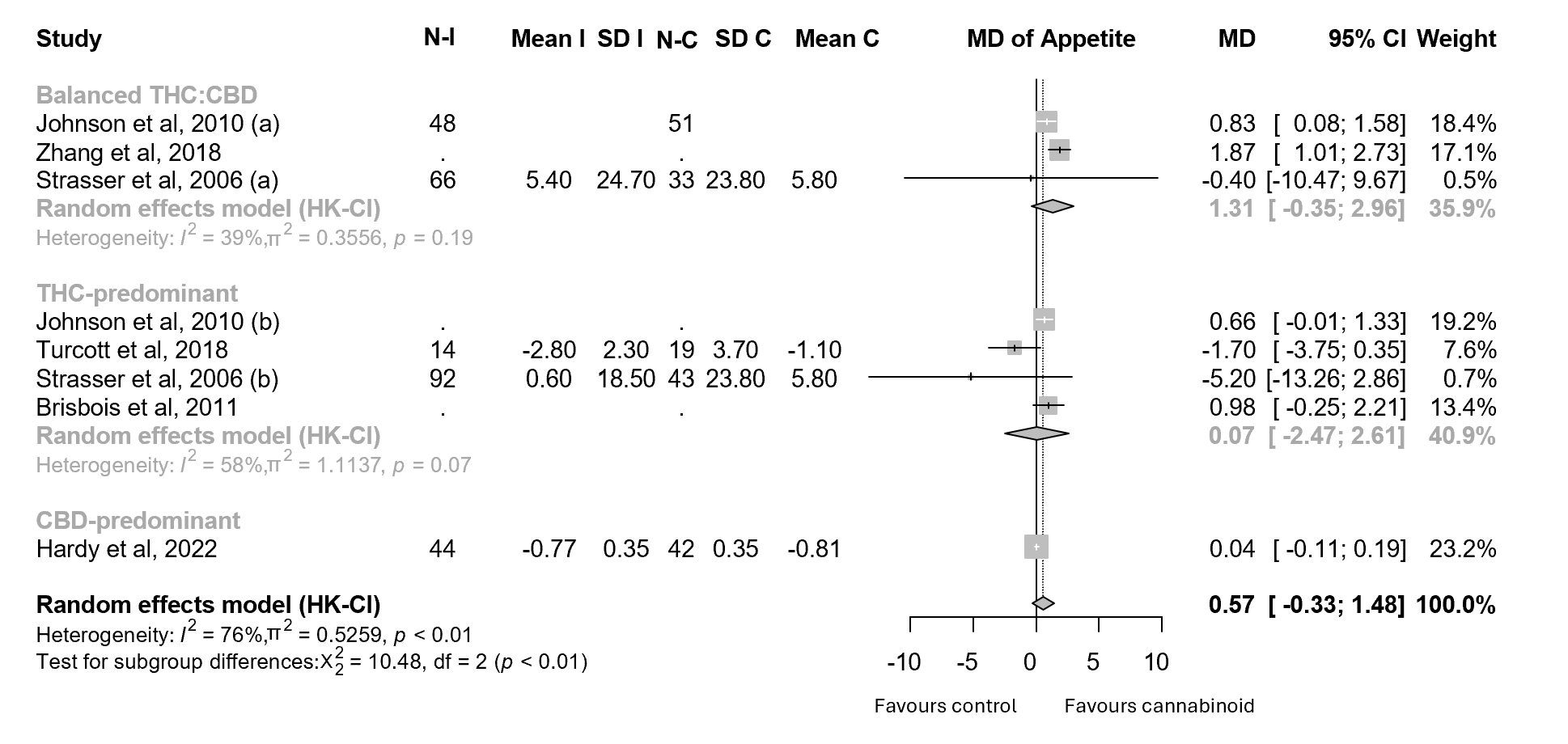


**Figure 1(S). Forest plots for the improvement of APPETITE of cancer patients undergoing cannabinoid treatment;** annotations: Tetrahydrocannabinol (THC), Cannabidiol (CBD), Sample Size (N), Intervention (I), Control (C), Standard Deviation (SD), Confidence Interval (CI), Mean Difference (MD), Standardised Mean Difference (SMD).


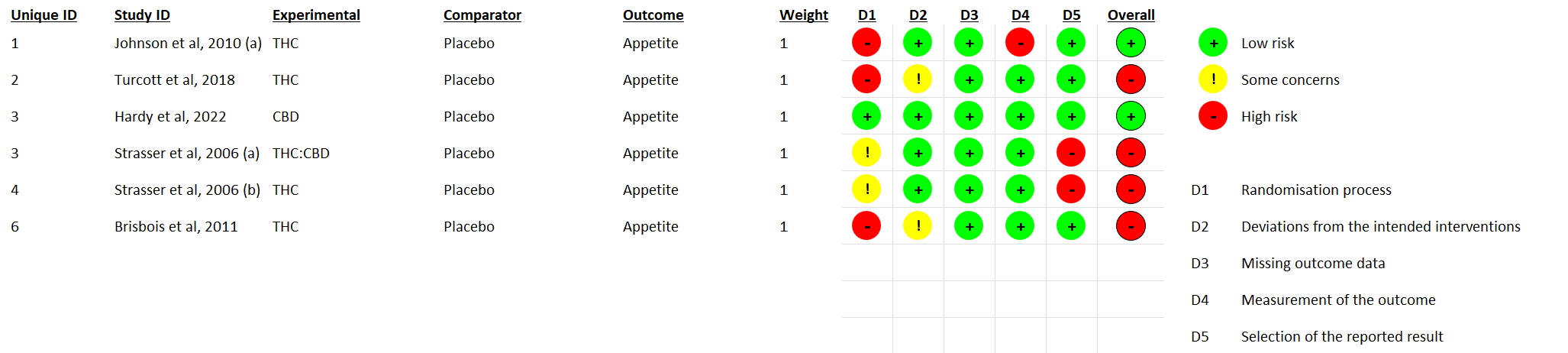


**Figure 2 (S). Risk of bias for studies included in the APPETITE analysis;** annotations: Tetrahydrocannabinol (THC), Cannabidiol (CBD).

1. ***Constipation***


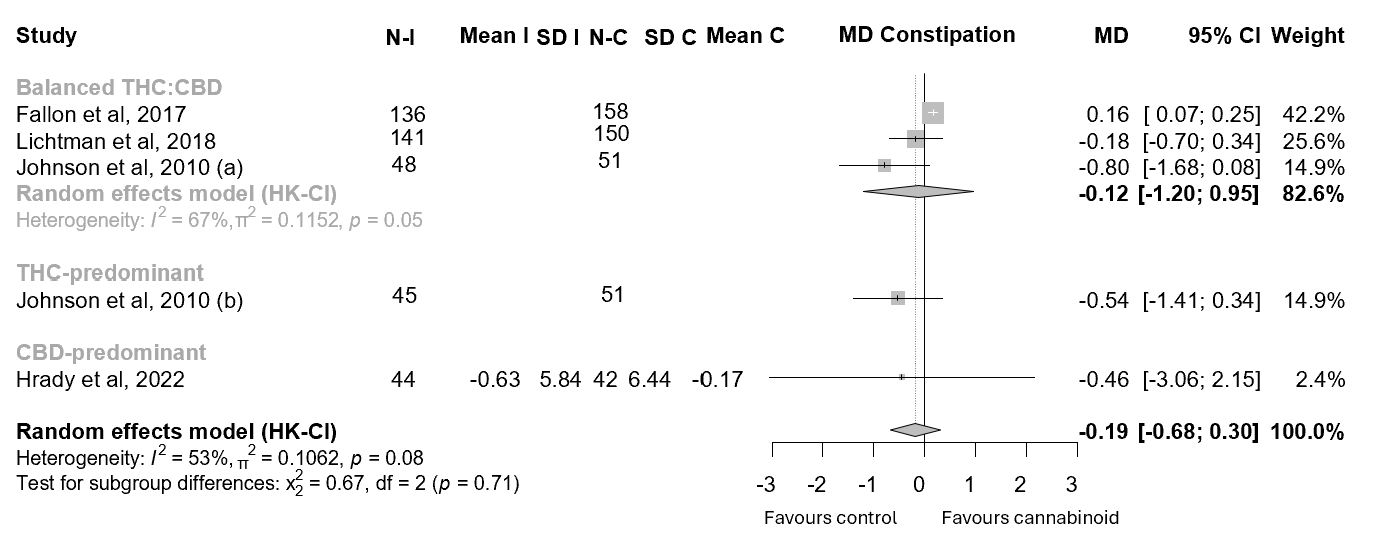


**Figure 3(S). Forest plots for the improvement of CONSTIPATION of cancer patients undergoing cannabinoid treatment;** annotations: Tetrahydrocannabinol (THC), Cannabidiol (CBD), Sample Size (N), Intervention (I), Control (C), Standard Deviation (SD), Confidence Interval (CI), Mean Difference (MD).


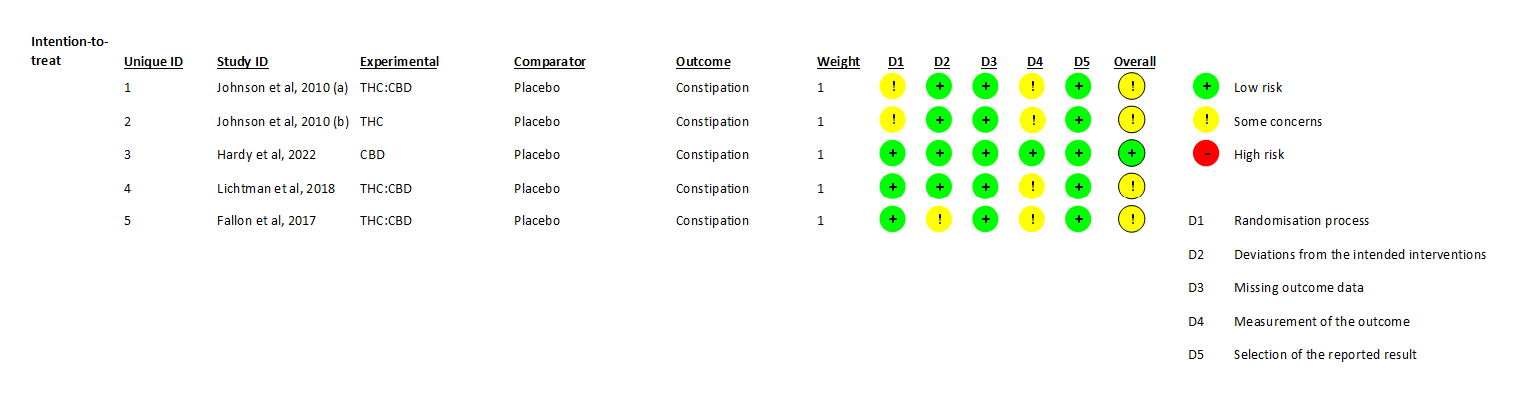


**Figure 4. Risk of bias for studies included in the CONSTIPATION analysis;** annotations: Tetrahydrocannabinol (THC), Cannabidiol (CBD).

1. ***Depression***


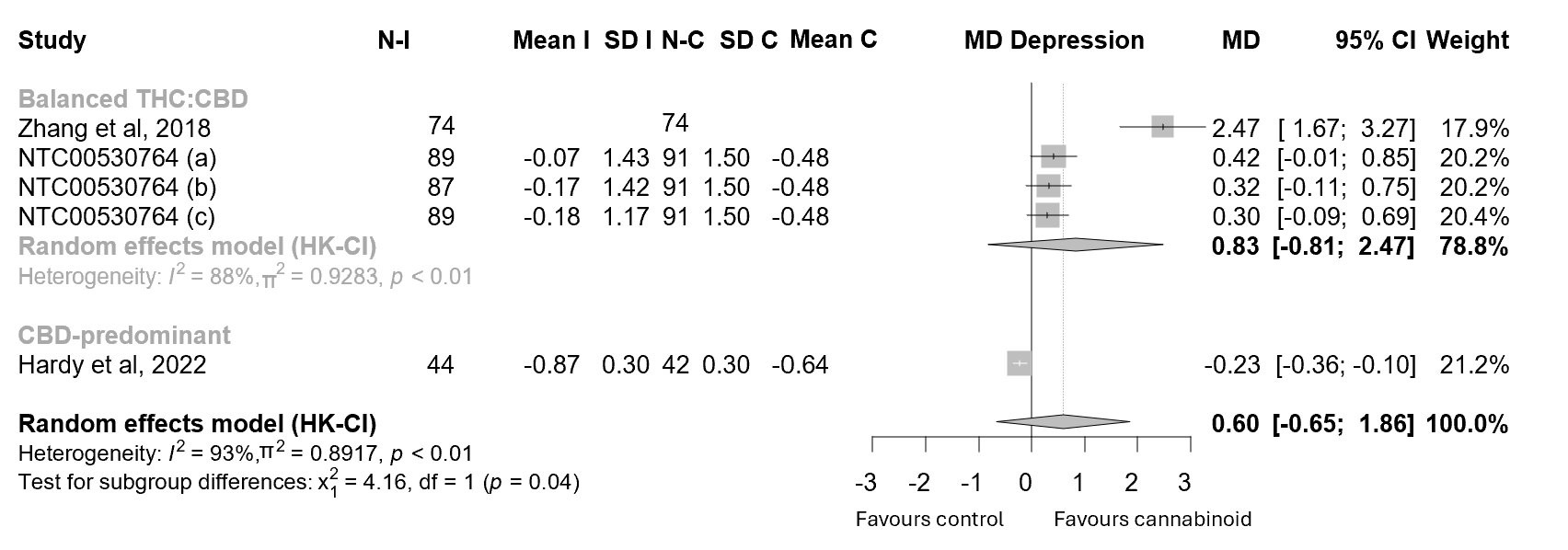


**Figure 5. Forest plots for the improvement of DEPRESSION of cancer patients undergoing cannabinoid treatment;** annotations: Tetrahydrocannabinol (THC), Cannabidiol (CBD), Sample Size (N), Intervention (I), Control (C), Standard Deviation (SD), Confidence Interval (CI), Mean Difference (MD).

1. ***Fatigue***


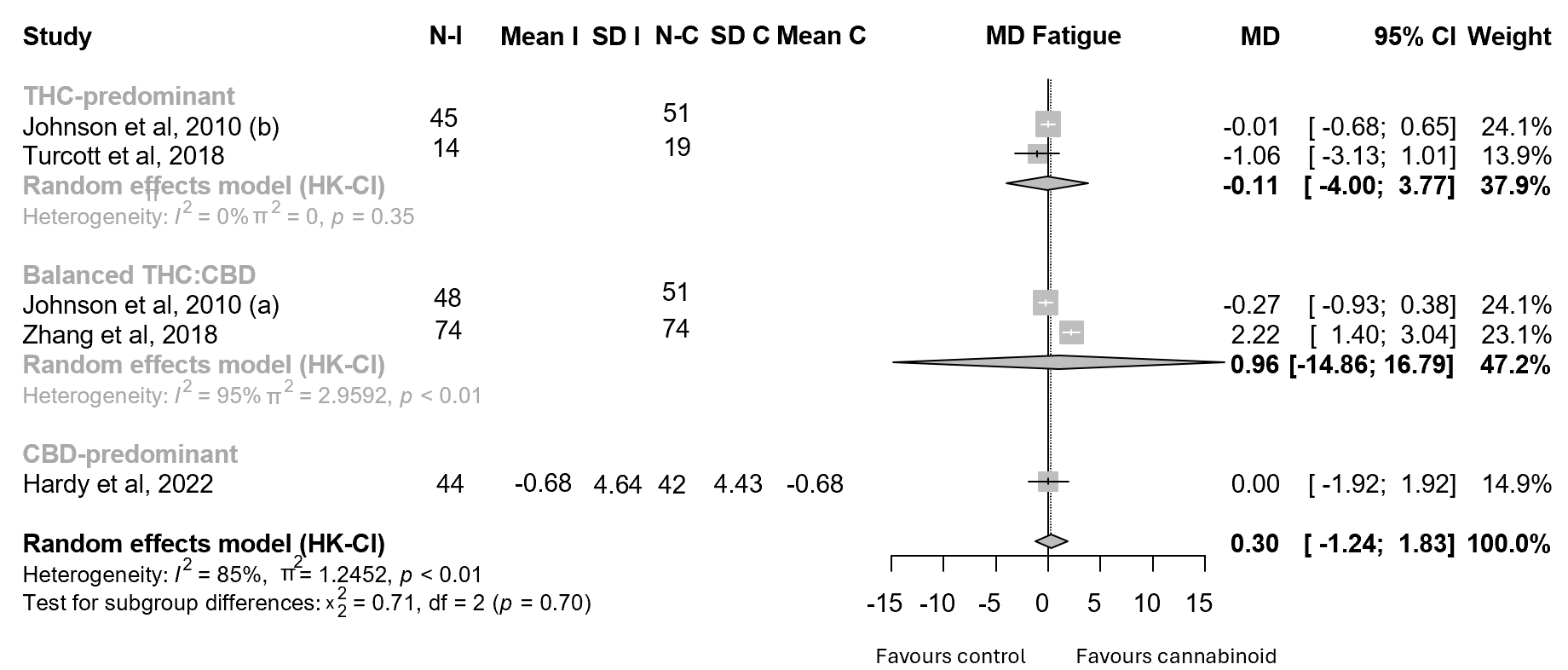


**Figure 6. Forest plots for the improvement of FATIGUE of cancer patients undergoing cannabinoid treatment;** annotations: Tetrahydrocannabinol (THC), Cannabidiol (CBD), Sample Size (N), Intervention (I), Control (C), Standard Deviation (SD), Confidence Interval (CI), Mean Difference (MD).


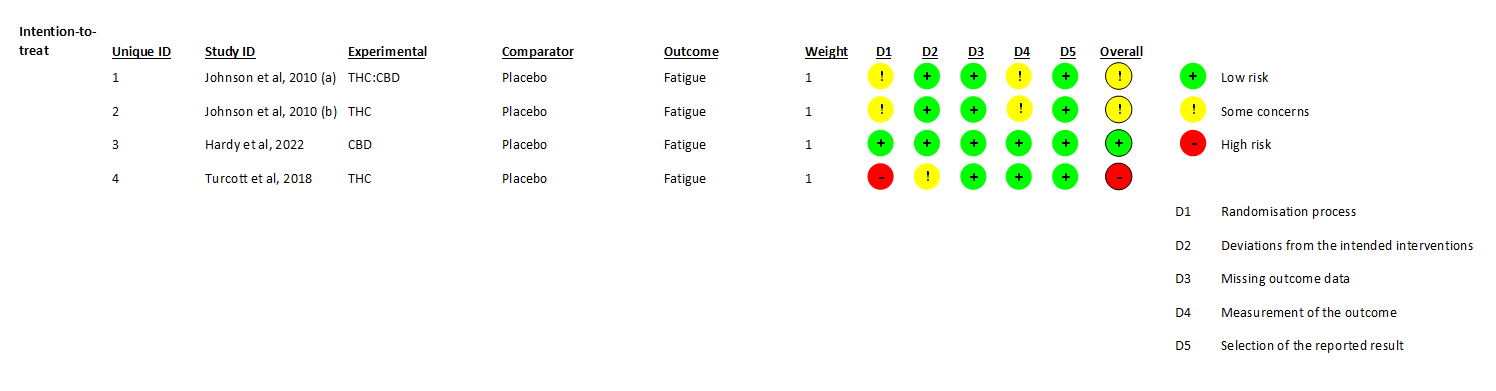


**Figure 7. Risk of bias for studies included in the FATIGUE analysis;** annotations: Tetrahydrocannabinol (THC), Cannabidiol (CBD).

1. ***Insomnia***


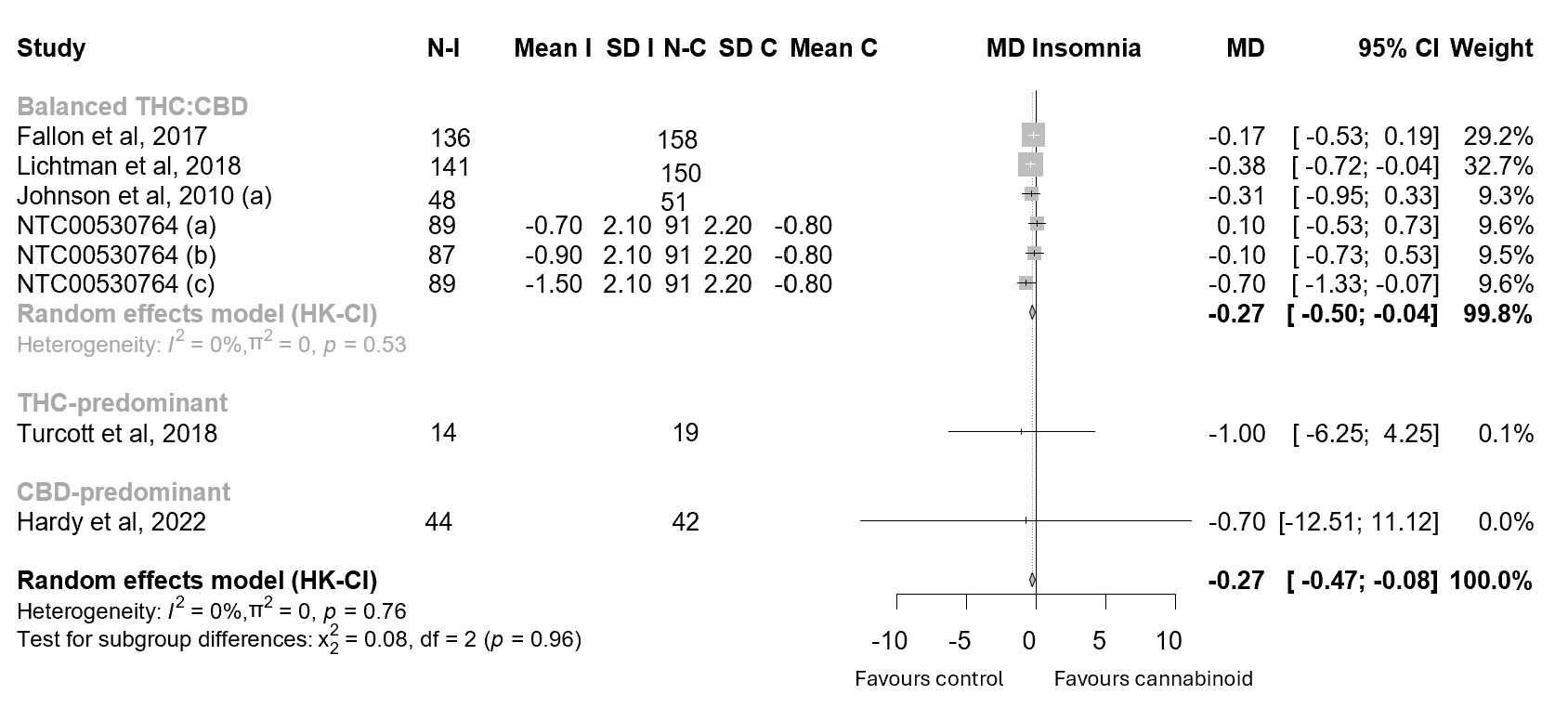


**Figure 8. Forest plots for the improvement of INSOMNIA of cancer patients undergoing cannabinoid treatment;** annotations: Tetrahydrocannabinol (THC), Cannabidiol (CBD), Sample Size (N), Intervention (I), Control (C), Standard Deviation (SD), Confidence Interval (CI), Mean Difference (MD).


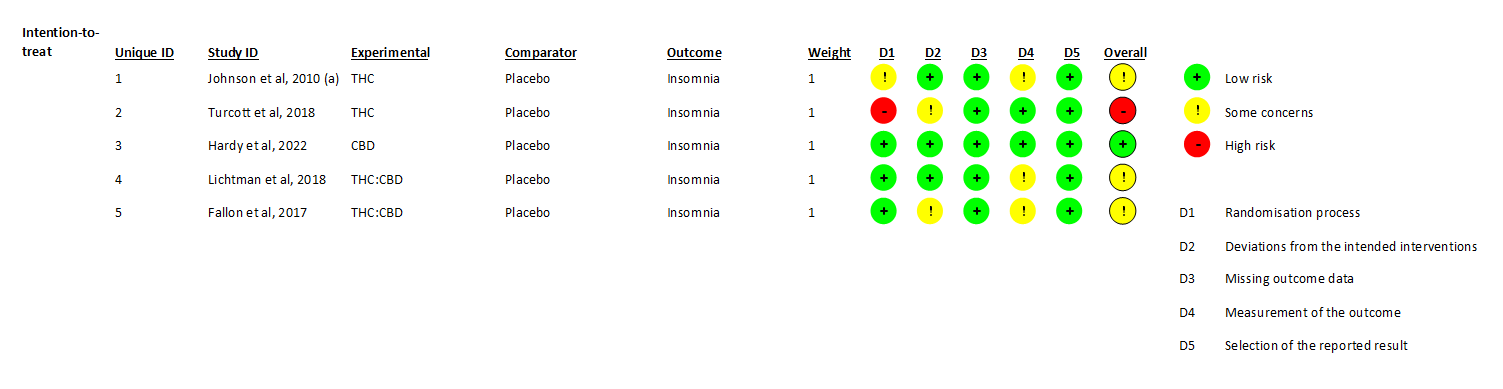


**Figure 9. Risk of bias for studies included in the INSOMNIA analysis;** annotations: Tetrahydrocannabinol (THC), Cannabidiol (CBD).

1. ***Mobility***


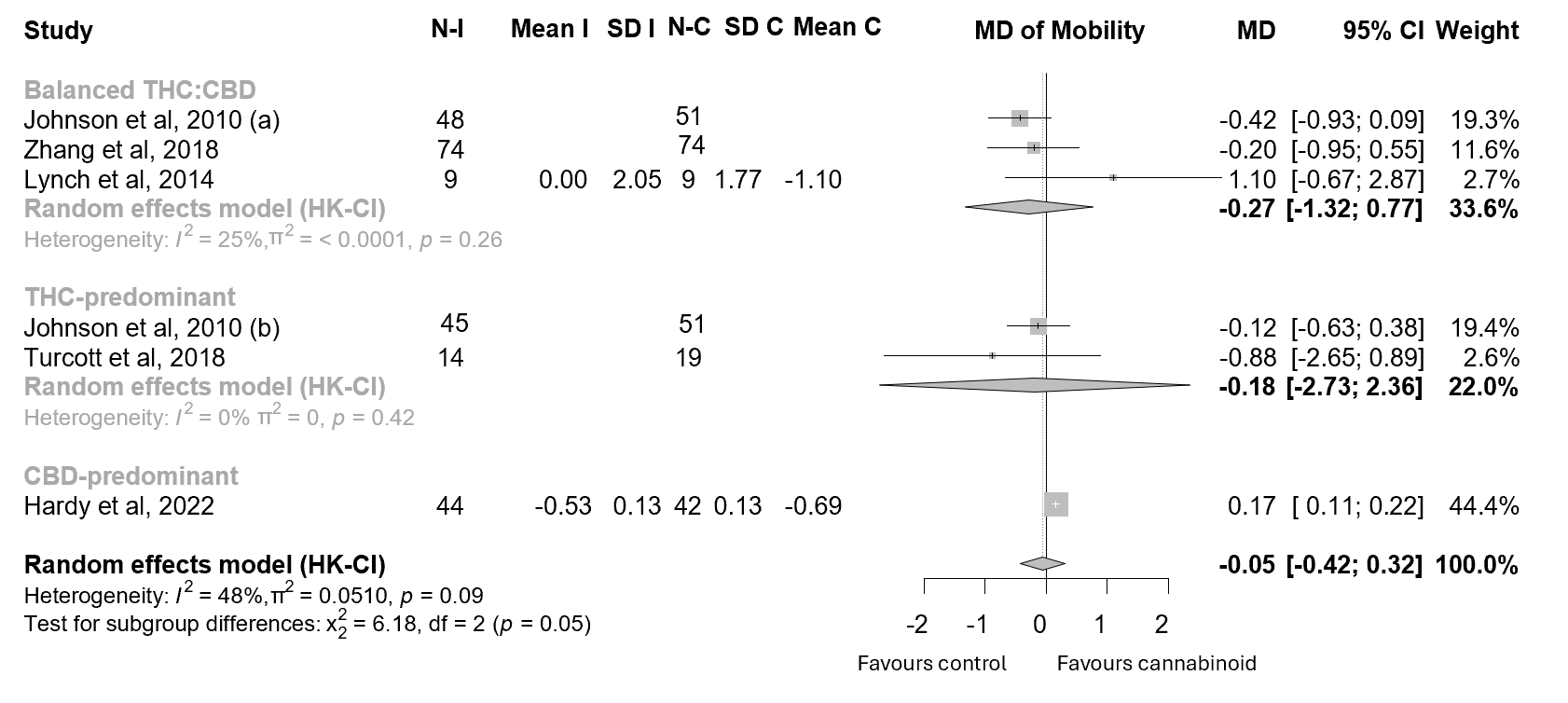


**Figure 10. Forest plots for the improvement of MOBILITY of cancer patients undergoing cannabinoid treatment;** annotations: Tetrahydrocannabinol (THC), Cannabidiol (CBD), Sample Size (N), Intervention (I), Control (C), Standard Deviation (SD), Confidence Interval (CI), Mean Difference (MD).


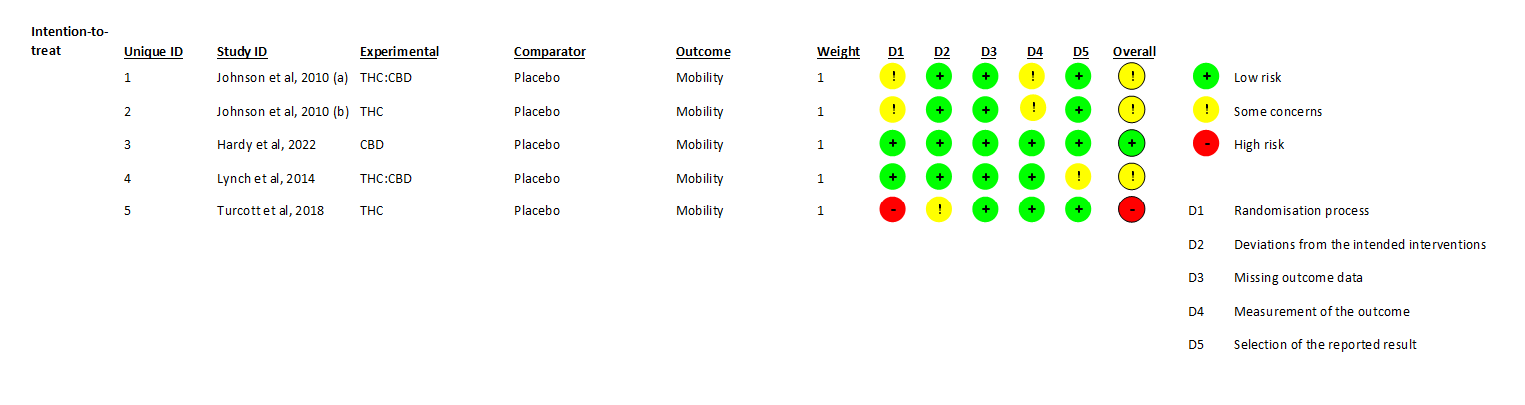


**Figure 11. Risk of bias for studies included in the MOBILITY analysis;** annotations: Tetrahydrocannabinol (THC), Cannabidiol (CBD).

1. ***Nausea***


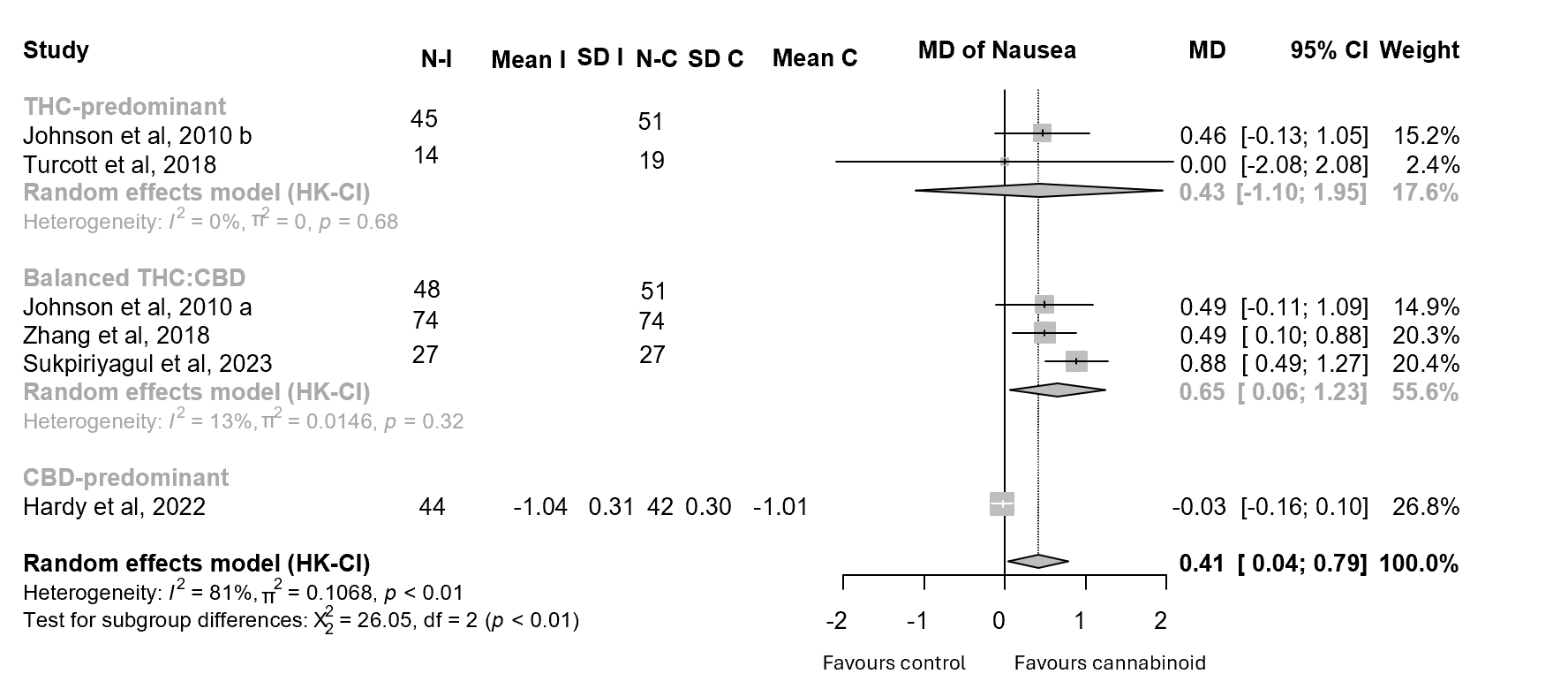


**Figure 12. Forest plots for the improvement of NAUSEA of cancer patients undergoing cannabinoid treatment;** annotations: Tetrahydrocannabinol (THC), Cannabidiol (CBD), Sample Size (N), Intervention (I), Control (C), Standard Deviation (SD), Confidence Interval (CI), Mean Difference (MD).


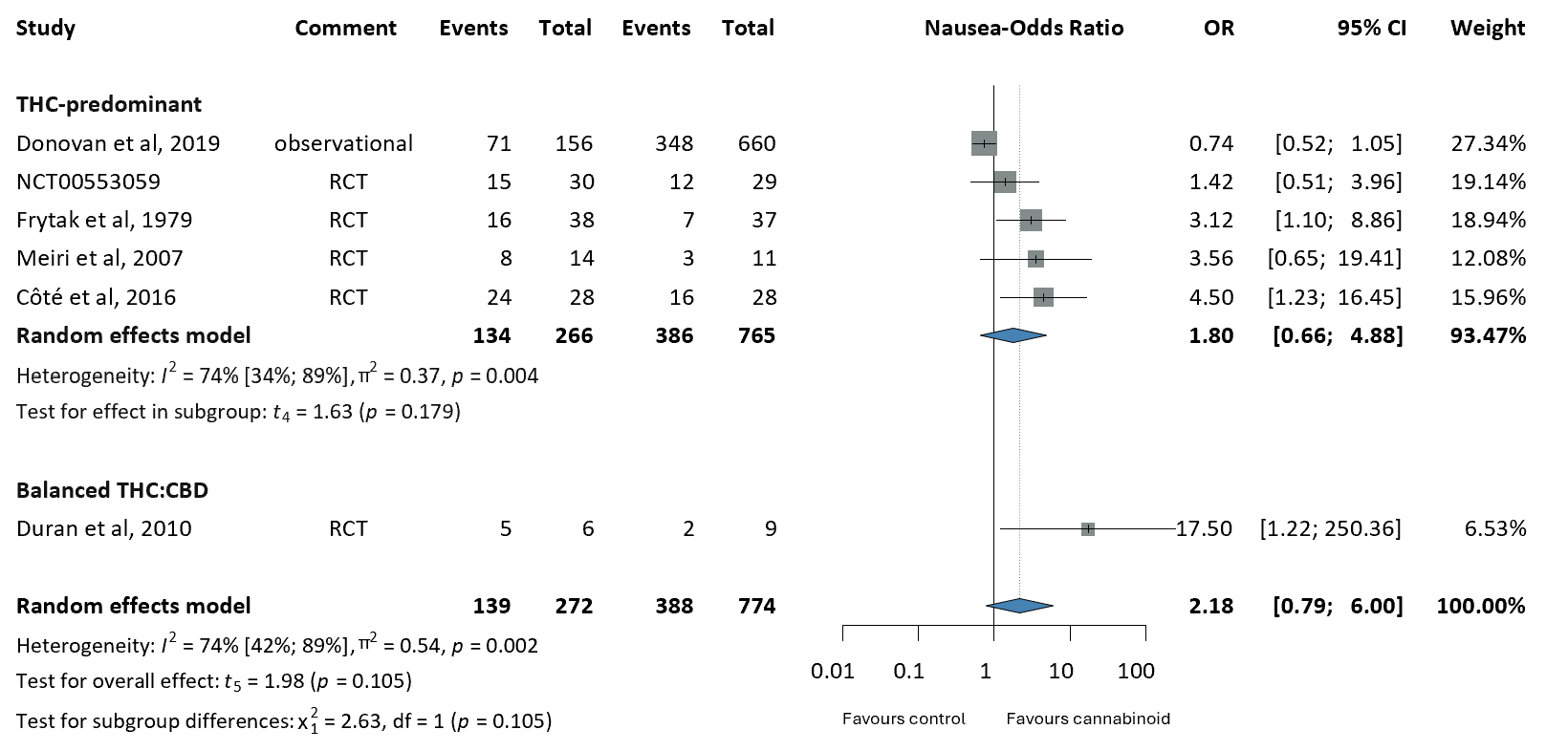


**Figure 13. Forest plots for the improvement of NAUSEA of cancer patients undergoing cannabinoid treatment;** annotations: Tetrahydrocannabinol (THC), Cannabidiol (CBD), Sample Size (N), Intervention (I), Control (C), Standard Deviation (SD), Confidence Interval (CI), Odds Ratio (OR)


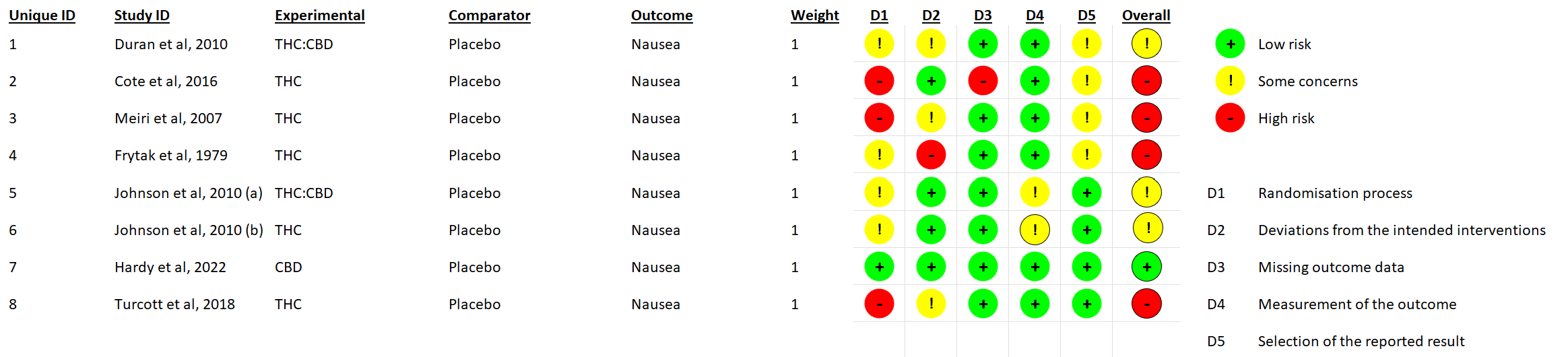


**Figure 14. Risk of bias for studies included in the NAUSEA analysis;** annotations: Tetrahydrocannabinol (THC), Cannabidiol (CBD).

1. ***Daily opioid consumption***


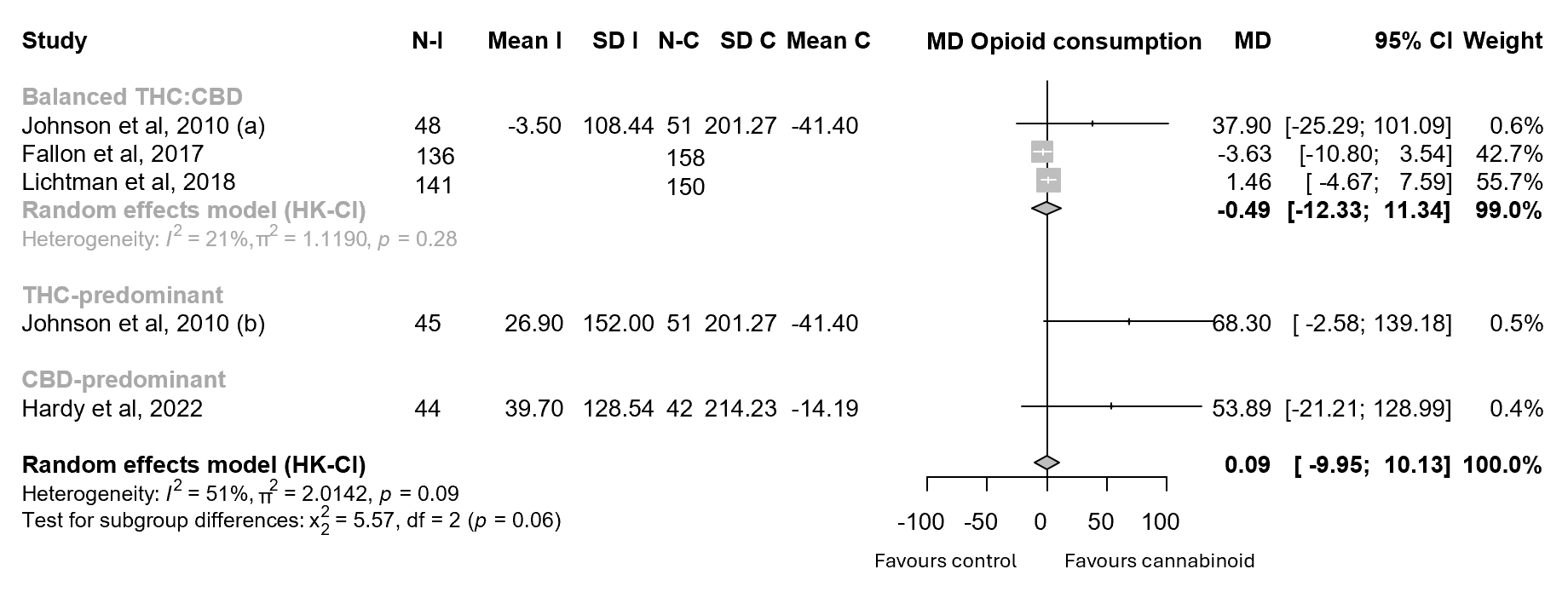


**Figure 15. Forest plots for the improvement of DAILY OPIOID CONSUMPTION of cancer patients undergoing cannabinoid treatment;** annotations: Tetrahydrocannabinol (THC), Cannabidiol (CBD), Sample Size (N), Intervention (I), Control (C), Standard Deviation (SD), Confidence Interval (CI), Mean Difference (MD).


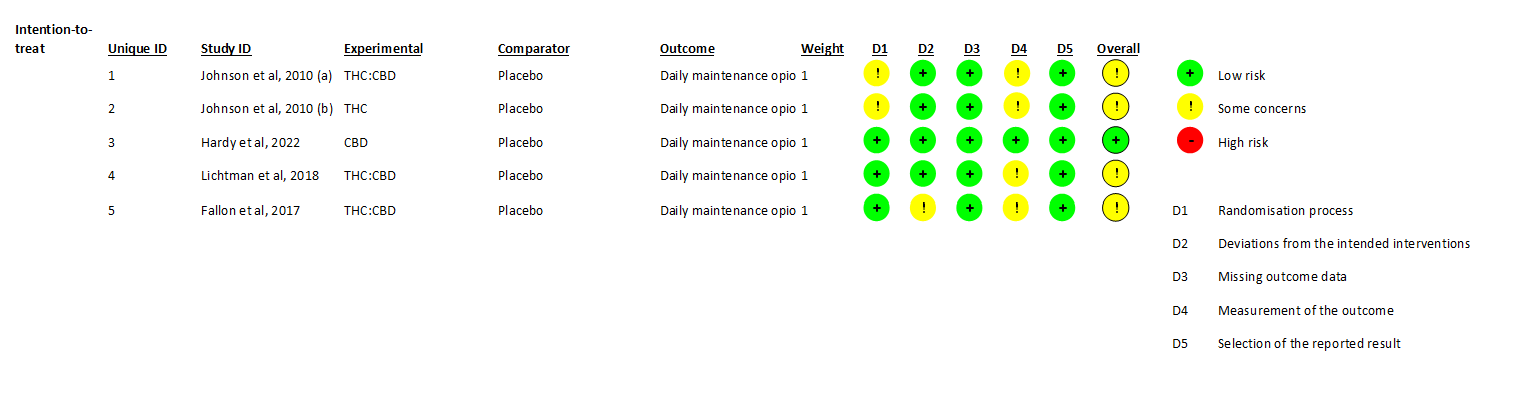


**Figure 16. Risk of bias for studies included in the DAILY OPIOID CONSUMPTION analysis;** annotations: Tetrahydrocannabinol (THC), Cannabidiol (CBD).

1. ***Pain***


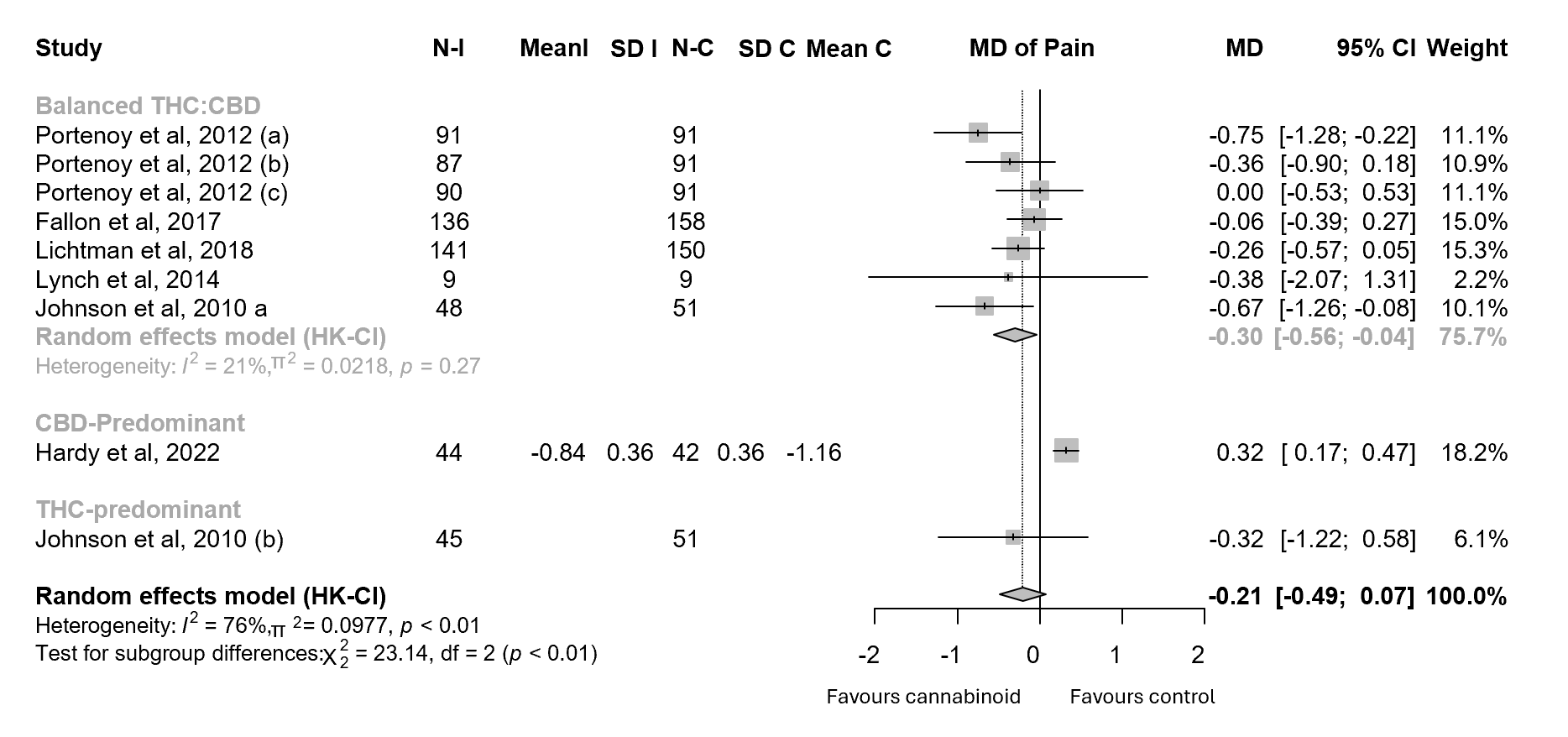


**Figure 17. Forest plots for the improvement of PAIN of cancer patients undergoing cannabinoid treatment;** annotations: Tetrahydrocannabinol (THC), Cannabidiol (CBD), Sample Size (N), Intervention (I), Control (C), Standard Deviation (SD), Confidence Interval (CI), Mean Difference (MD), Standardised Mean Difference (SMD).


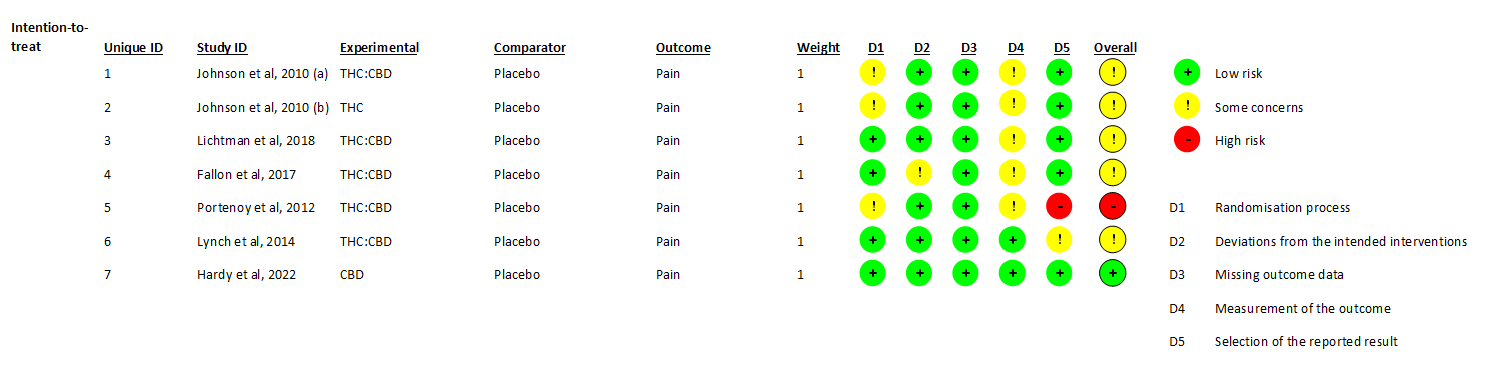


**Figure 18. Risk of bias for studies included in the PAIN analysis;** annotations: Tetrahydrocannabinol (THC), Cannabidiol (CBD).

1. ***Quality of life (QoL)***


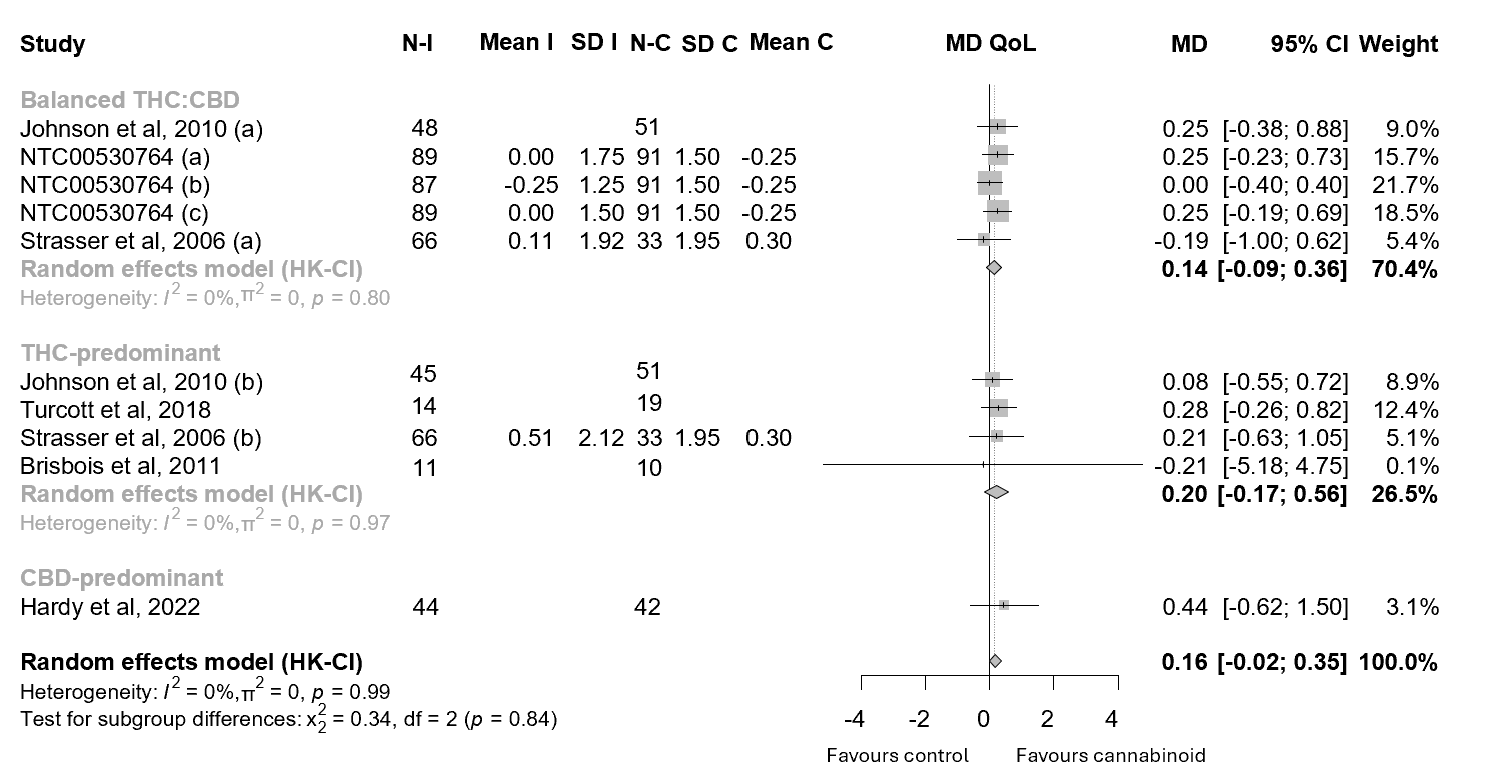


**Figure 19. Forest plots for the improvement of QoL of cancer patients undergoing cannabinoid treatment;** annotations: Tetrahydrocannabinol (THC), Cannabidiol (CBD), Sample Size (N), Intervention (I), Control (C), Standard Deviation (SD), Confidence Interval (CI), Mean Difference (MD).


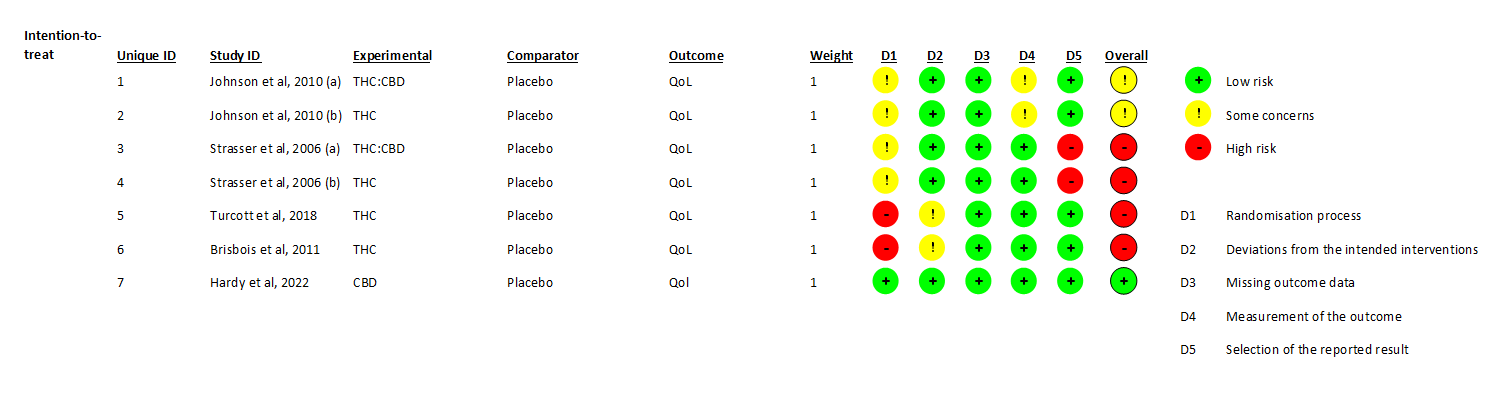


**Figure 20. Risk of bias for studies included in the QoL analysis;** annotations: Tetrahydrocannabinol (THC), Cannabidiol (CBD).

1. ***Subject’s global impression of change***


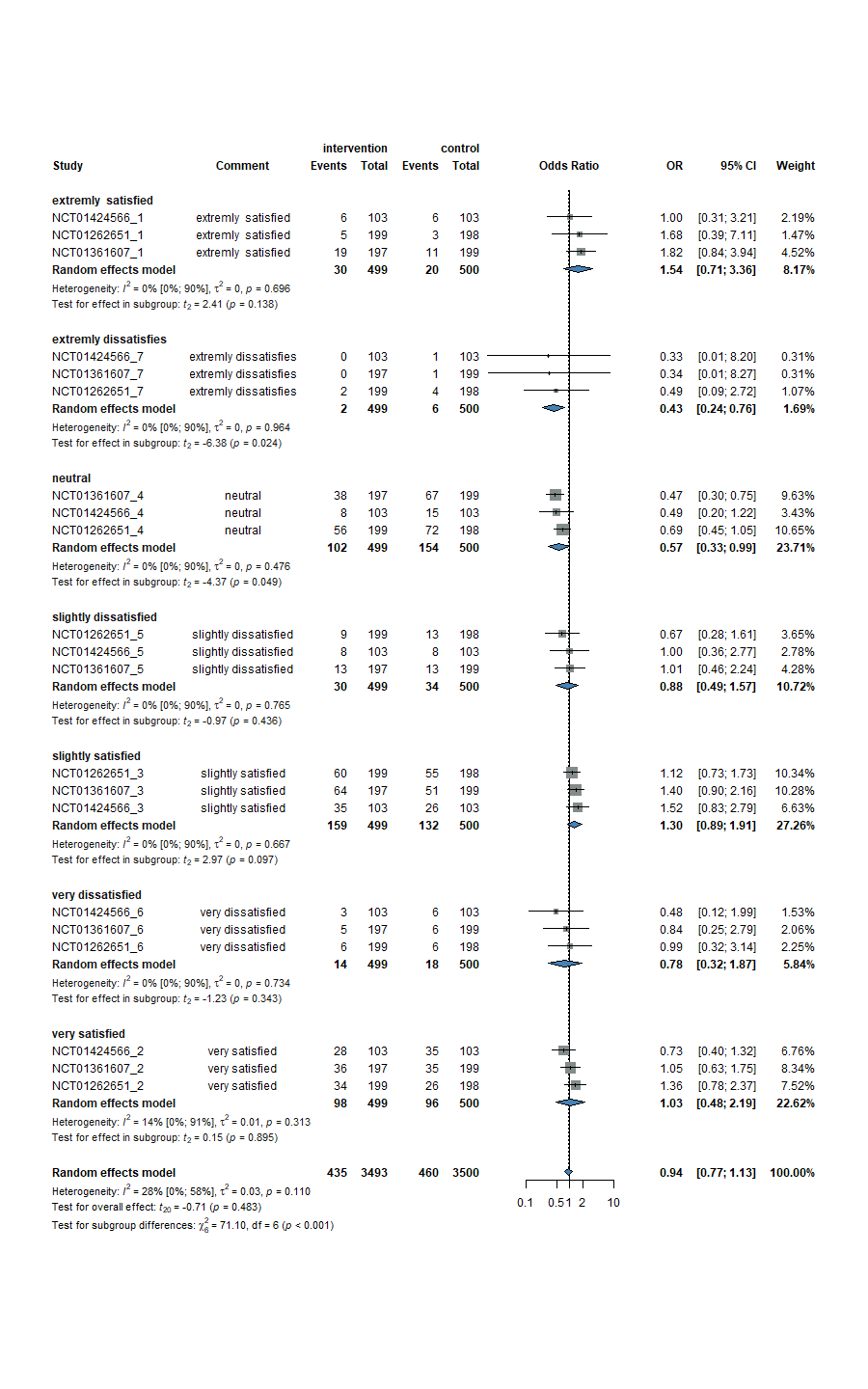


**Figure 21. Forest plots for Subject’s global impression of change for cancer patients undergoing cannabinoid treatment;** annotations: Tetrahydrocannabinol (THC), Cannabidiol (CBD), Sample Size (N), Intervention (I), Control (C), Standard Deviation (SD), Confidence Interval (CI), Odds Ratio (OR).

1. **One-arm studies (baseline vs after intervention)**
2. ***Anxiety***


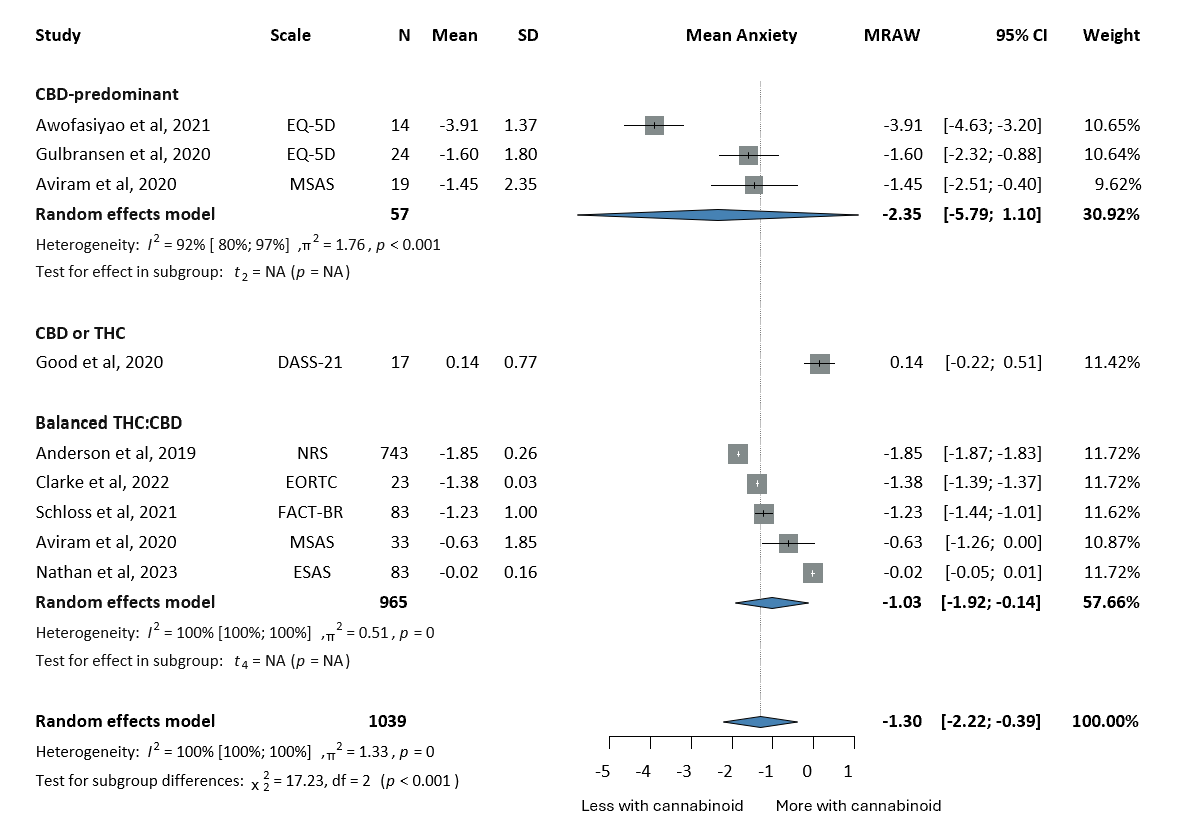


**Figure 22. Forest plots for the improvement of ANXIETY of cancer patients undergoing cannabinoid treatment;** annotations: Tetrahydrocannabinol (THC), Cannabidiol (CBD), Sample Size (N), Standard Deviation (SD), Confidence Interval (CI), Raw or untransformed mean (MRAW), European Organisation For Research And Treatment of Cancer- Core Quality of Life questionnaire (EORTC-QLQ30), Numerical Rating Scale (NRS), Funtional Assessment of Cancer Therapy (FACT), EuroQol (EQ-5D), Edmonton Symptom Assessment Scale (ESAS), Memorial Symptom Assessment Scale Scale (MSAS), Depression, Anxiety and Stress Scale – 21 (DASS-21).


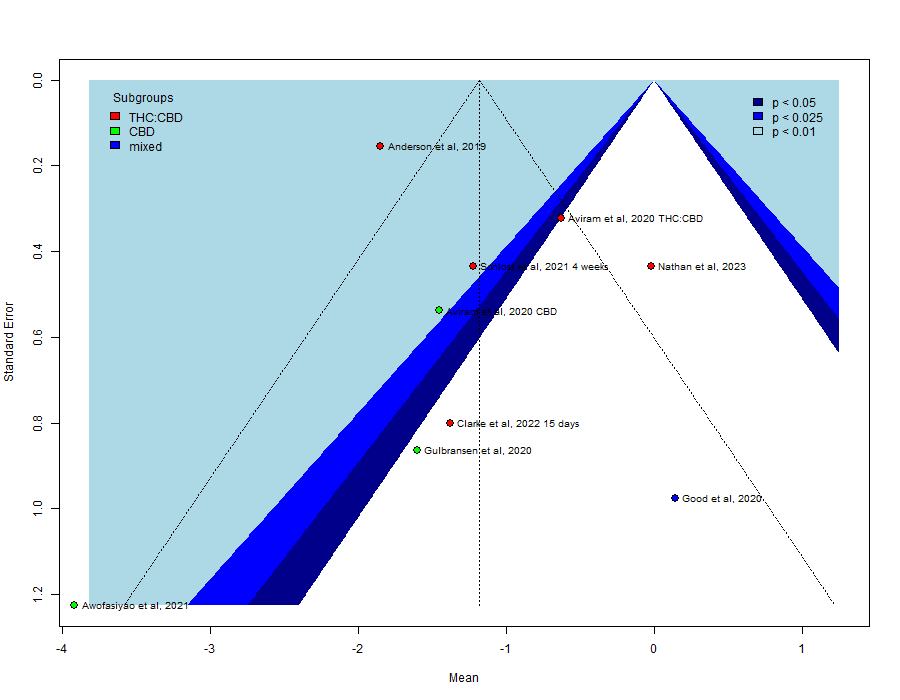


**Figure 23. Publication bias for ANXIETY outcome visualized on funnel plots;** annotations: Tetrahydrocannabinol (THC), Cannabidiol (CBD).

1. ***Appetite loss***


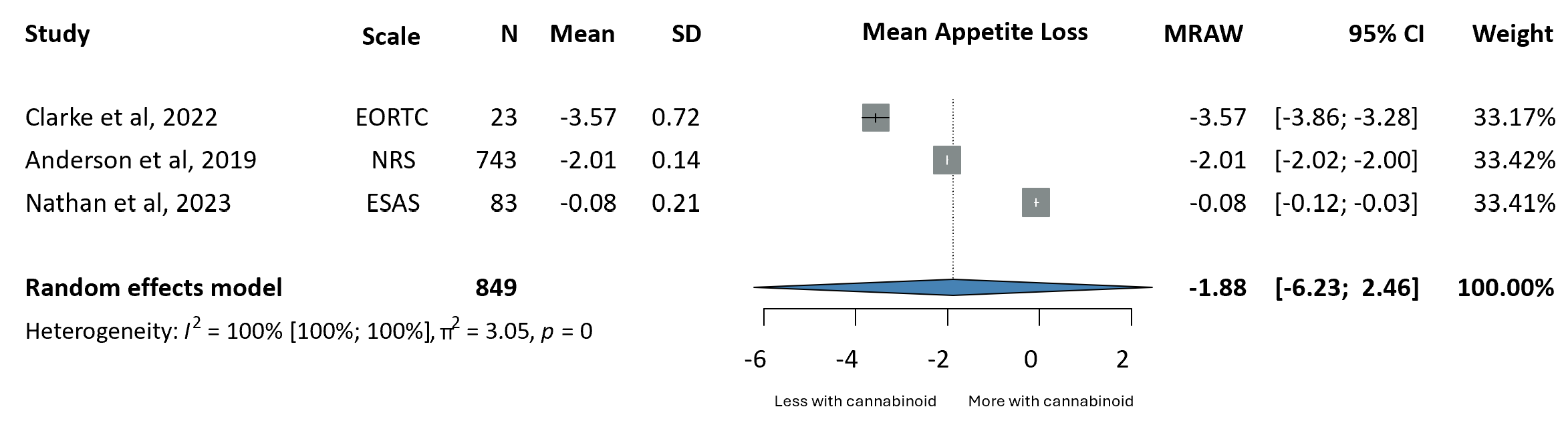


**Figure 24. Forest plots for APPETITE LOSS of cancer patients undergoing cannabinoid treatment;** annotations: Tetrahydrocannabinol (THC), Cannabidiol (CBD), Sample Size (N), Standard Deviation (SD), Confidence Interval (CI), Raw or untransformed mean (MRAW), European Organisation For Research And Treatment of Cancer- Core Quality of Life questionnaire (EORTC-QLQ30), Numerical Rating Scale (NRS), Edmonton Symptom Assessment Scale (ESAS), Memorial Symptom Assessment Scale Scale (MSAS).


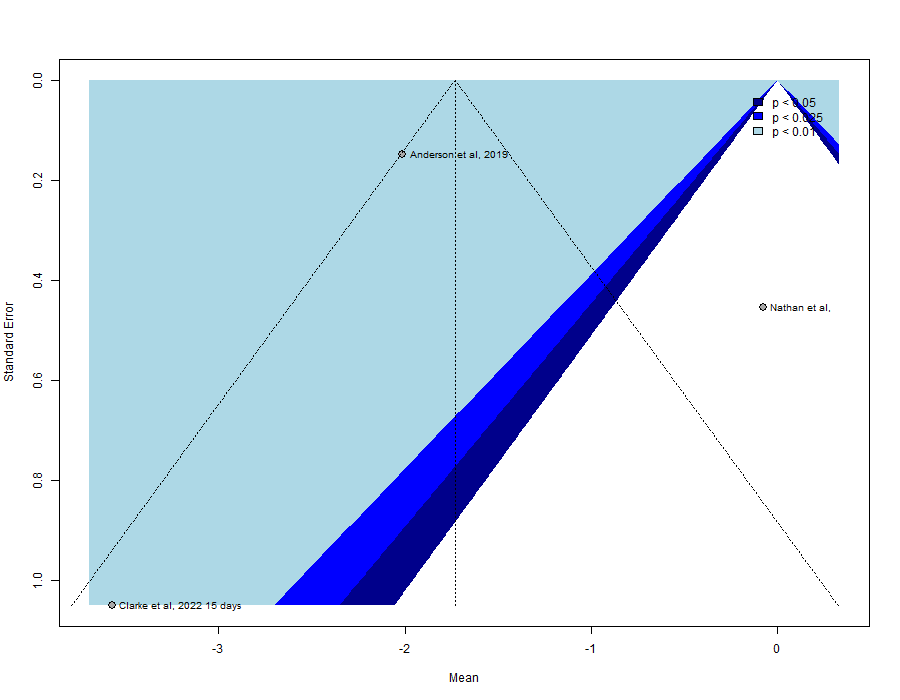


**Figure 24. Publication bias for APPETITE LOSS outcome visualized on funnel plots;** annotations: Tetrahydrocannabinol (THC), Cannabidiol (CBD).

1. ***Insomnia***


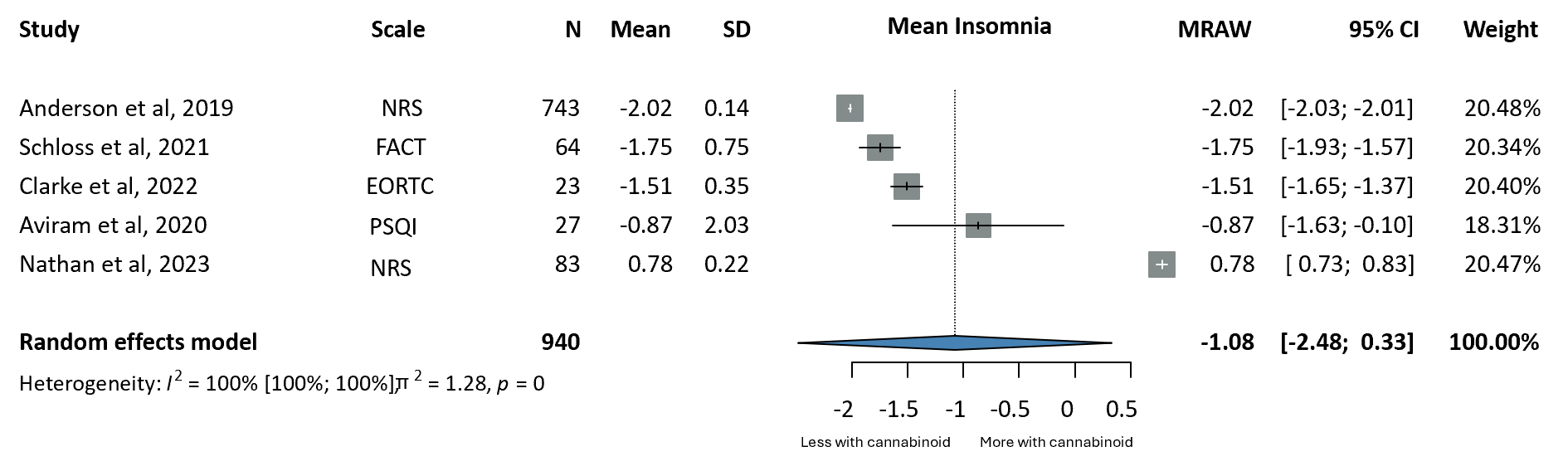


**Figure 25. Forest plots for the effects of cannabinoids on INSOMNIA;** annotations: Tetrahydrocannabinol (THC), Cannabidiol (CBD), Sample Size (N), Standard Deviation (SD), Confidence Interval (CI), Raw or untransformed mean (MRAW), European Organisation For Research And Treatment of Cancer- Core Quality of Life questionnaire (EORTC-QLQ30), Numerical Rating Scale (NRS), Edmonton Symptom Assessment Scale (ESAS), Funtional Assessment of Cancer Therapy (FACT), [Pittsburgh Sleep Quality Index (PSQI).](https://www.med.upenn.edu/cbti/assets/user-content/documents/Pittsburgh%20Sleep%20Quality%20Index%20(PSQI).pdf)


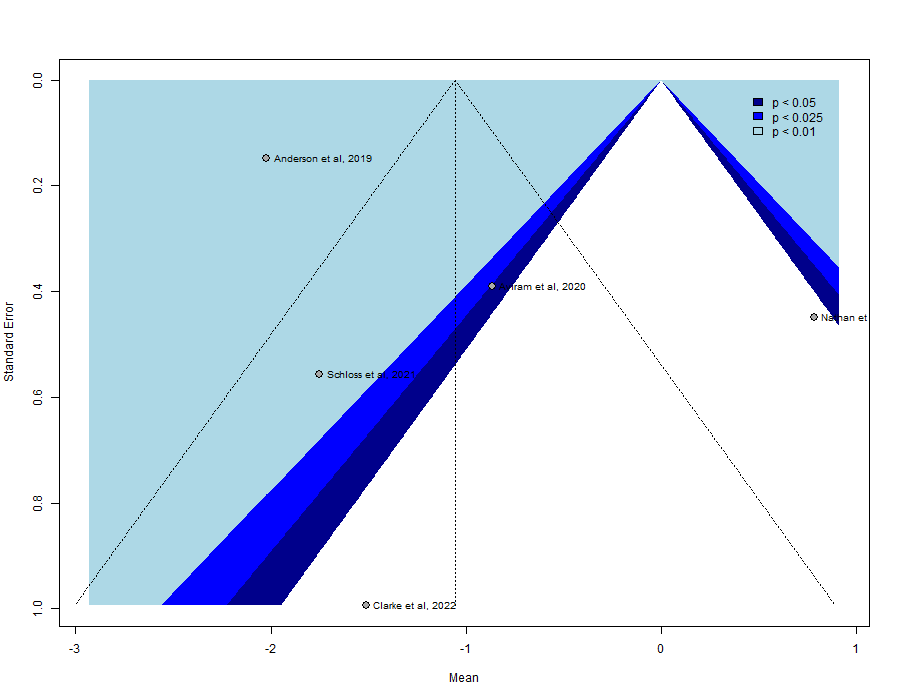


**Figure 26. Publication bias for INSOMNIA outcome visualized on funnel plots;** annotations: Tetrahydrocannabinol (THC), Cannabidiol (CBD).

1. ***Nausea***


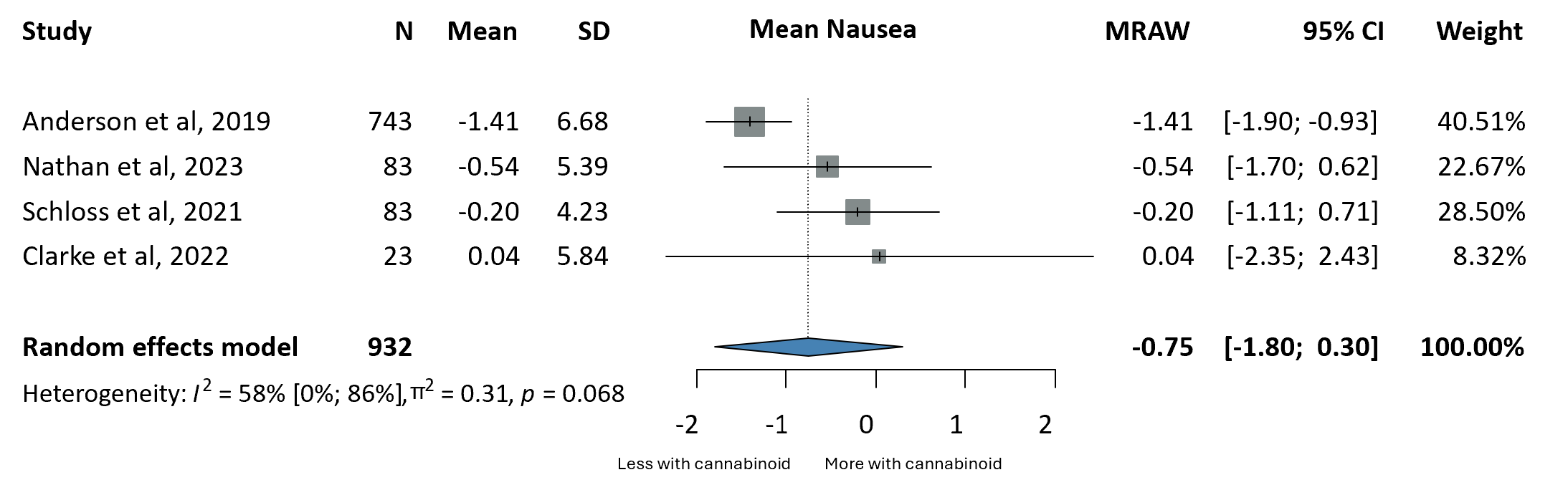


**Figure 27. Forest plots for the effects of cannabinoids on NAUSEA;** annotations: Tetrahydrocannabinol (THC), Cannabidiol (CBD), Sample Size (N), Standard Deviation (SD), Confidence Interval (CI), Raw or untransformed mean (MRAW).


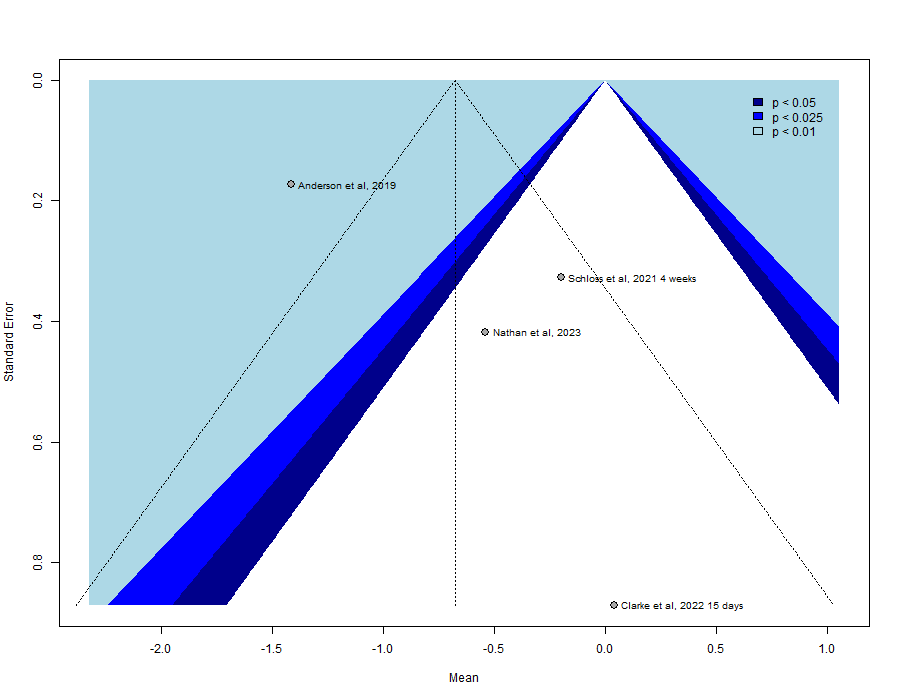


**Figure 28. Publication bias for NAUSEA outcome visualized on funnel plots;** annotations: Tetrahydrocannabinol (THC), Cannabidiol (CBD).

1. ***Pain***


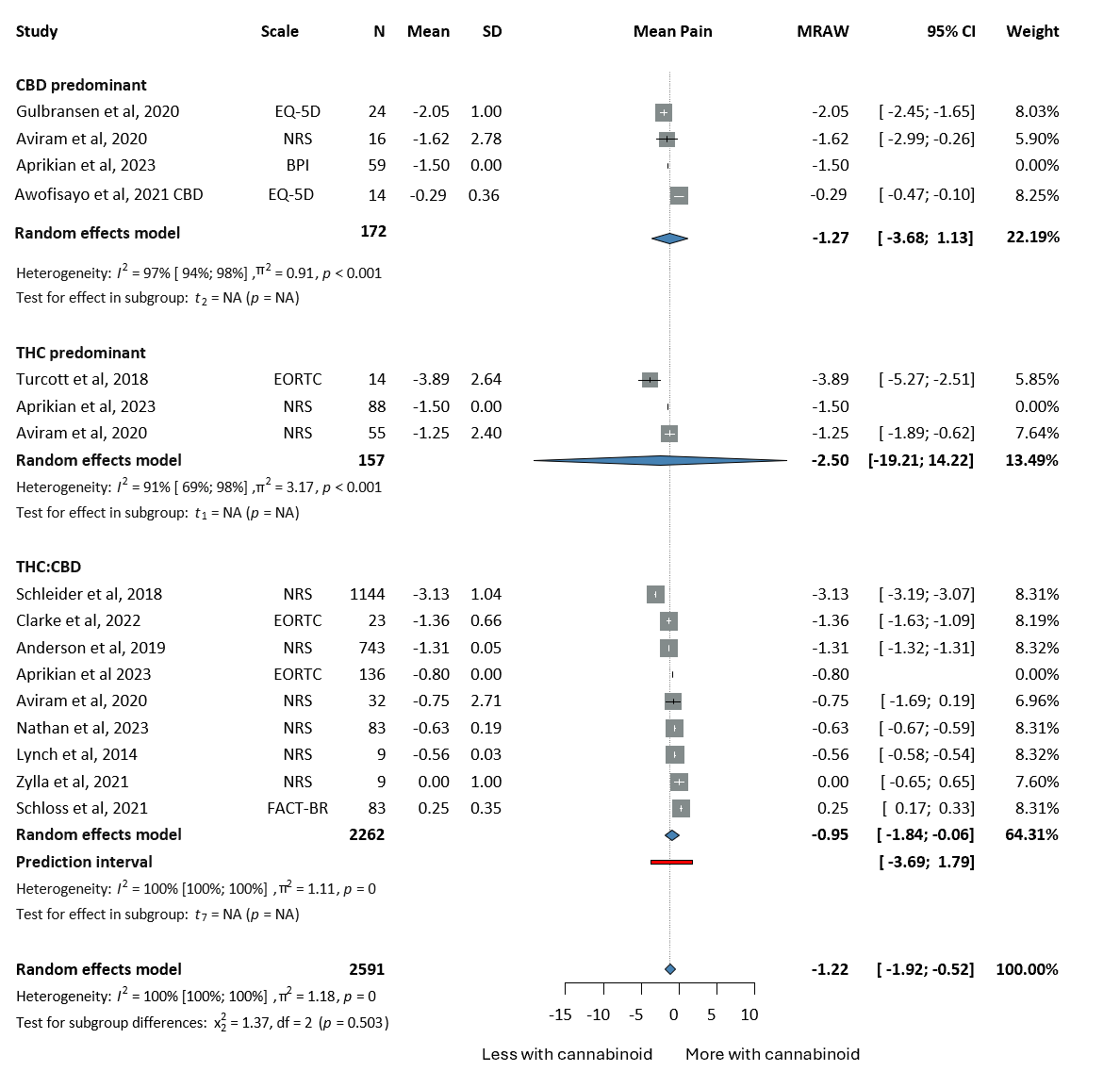


**Figure 29. Forest plots for the improvement of PAIN of cancer patients undergoing cannabinoid treatment;** annotations: Tetrahydrocannabinol (THC), Cannabidiol (CBD), Sample Size (N), Standard Deviation (SD), Confidence Interval (CI), Raw or untransformed mean (MRAW), European Organisation For Research And Treatment of Cancer- Core Quality of Life questionnaire (EORTC-QLQ30), Numerical Rating Scale (NRS), Brief Pain Inventory (BPI), Funtional Assessment of Cancer Therapy (FACT), EuroQol (EQ-5D), Edmonton Symptom Assessment Scale (ESAS).


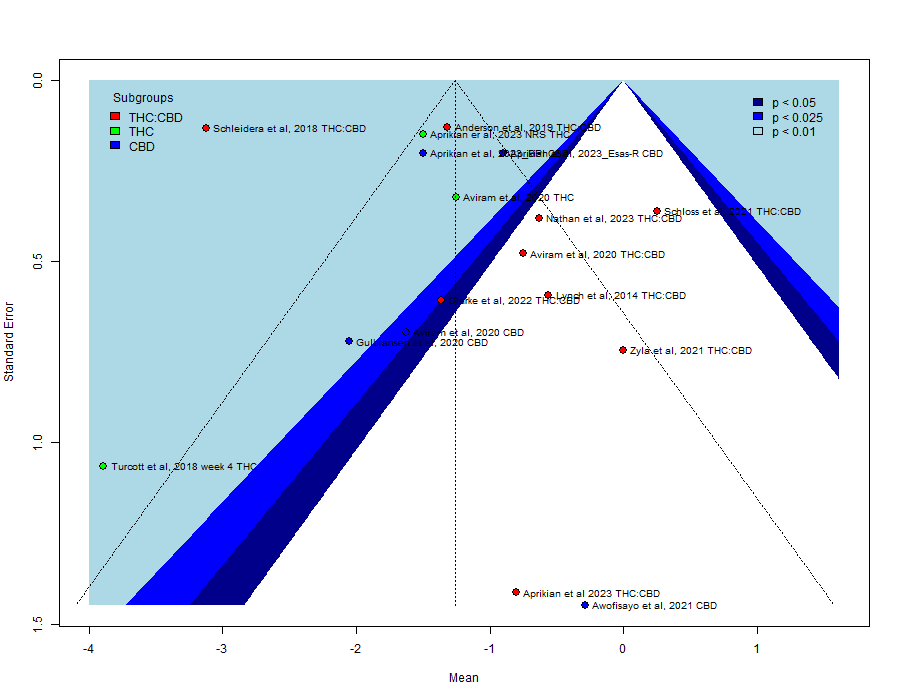


**Figure 30. Publication bias for PAIN outcome visualized on funnel plots;** annotations: Tetrahydrocannabinol (THC), Cannabidiol (CBD).

1. ***Quality of life (QoL)***


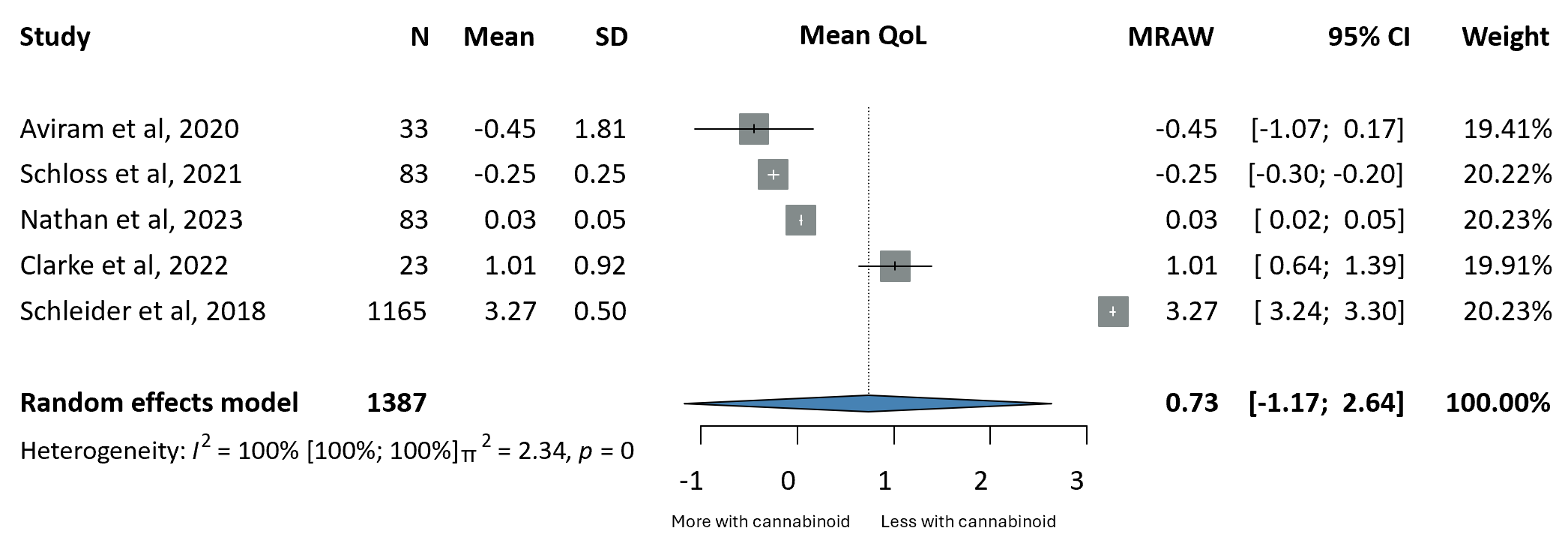


**Figure 31. Forest plots for the improvement of QoL of cancer patients undergoing cannabinoid treatment;** annotations: Tetrahydrocannabinol (THC), Cannabidiol (CBD), Sample Size (N), Standard Deviation (SD), Confidence Interval (CI), Raw or untransformed mean (MRAW).


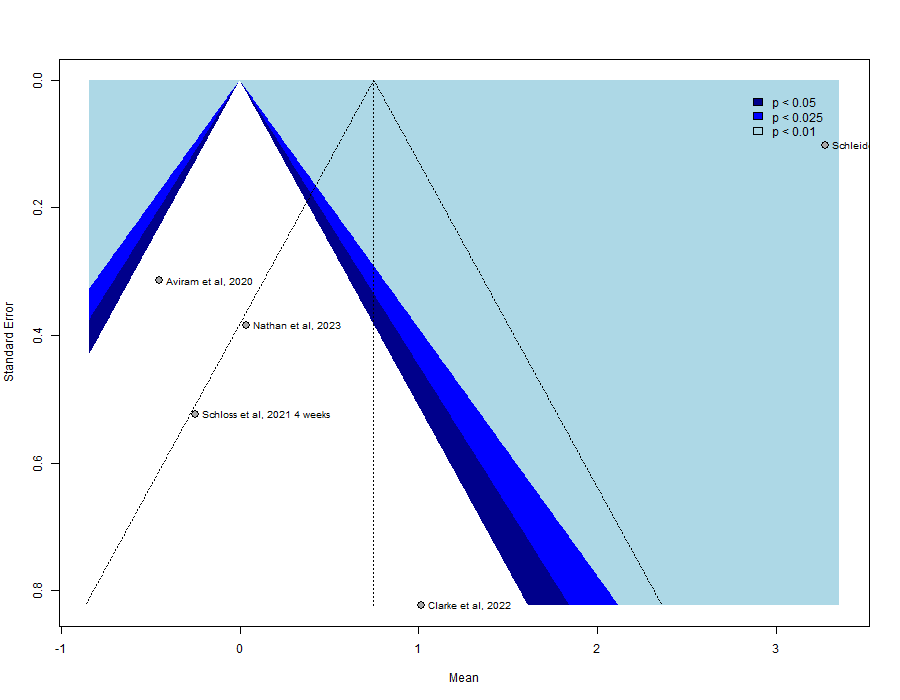


**Figure 32. Publication bias for QoL outcome visualized on funnel plots;** annotations: Tetrahydrocannabinol (THC), Cannabidiol (CBD).

| **Study** | **D1** | **D2** | **D3** | **D4** | **D5** | **D6** | **D7** | **Overall** |
| --- | --- | --- | --- | --- | --- | --- | --- | --- |
| Wongkongdech et al, 2022 |  |  |  |  |  |  |  |  |
| Zhang et al, 2018 |  |  |  |  |  |  |  |  |
| Chang et al, 2019 |  |  |  |  |  |  |  |  |
| Donovan et al, 2019 |  |  |  |  |  |  |  |  |
| Aviram et al, 2020 |  |  |  |  |  |  |  |  |
| Clarke et al, 2022 |  |  |  |  |  |  |  |  |
| Nathan et al, 2023 |  |  |  |  |  |  |  |  |
| Anderson et al, 2019 |  |  |  |  |  |  |  |  |
| Bar-Sela et al, 2013 |  |  |  |  |  |  |  |  |
| Aprikian et al, 2023 |  |  |  |  |  |  |  |  |
| Schleidera et al, 2018 |  |  |  |  |  |  |  |  |
| Awofisayo et al, 2021 |  |  |  |  |  |  |  |  |
| Gulbransen et al, 2020 |  |  |  |  |  |  |  |  |
| Pitchard et al, 2019 |  |  |  |  |  |  |  |  |
| Good et al, 2020 |  |  |  |  |  |  |  |  |

**Table 3. Risk of bias for studies included in one-arm studies analysis.**

| Low risk | D1=Bias due to confounding |
| --- | --- |
|  | D2=Selection of participants |
| Some risk | D3=Classification of interventions |
|  | D4-Deviation from intended interventions |
| High risk | D5=Missing data |
|  | D6=Measurement of outcomes |
|  | D7=Selection of reported results |

1. **Two-arm studies (intervention vs Prochlorperazine)**
2. ***Nausea***


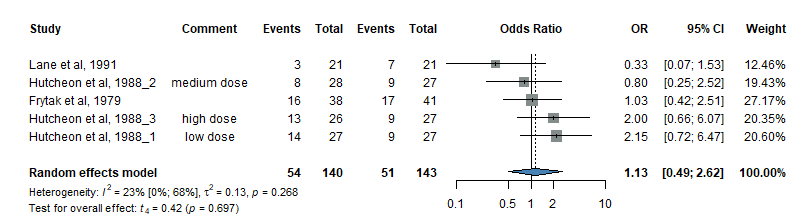


**Figure 33. Forest plots for the improvement of NAUSEA of cancer patients undergoing cannabinoid treatment versus Prochlorperazie treatment;** annotations: Tetrahydrocannabinol (THC), Cannabidiol (CBD), Sample Size (N), Standard Deviation (SD), Confidence Interval (CI), Odds Ratio (OR).

1. ***Vomiting***


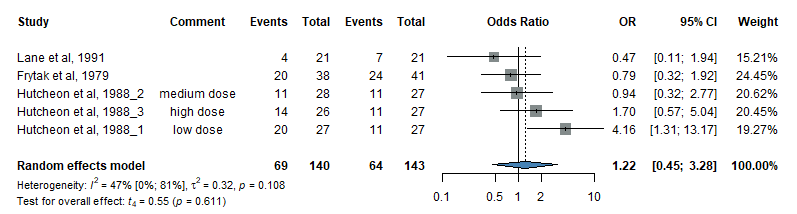


**Figure 34. Forest plots for the improvement of VOMITING of cancer patients undergoing cannabinoid treatment versus Prochlorperazie treatment;** annotations: Tetrahydrocannabinol (THC), Cannabidiol (CBD), Sample Size (N), Standard Deviation (SD), Confidence Interval (CI), Odds Ratio (OR).

1. ***Patient’s preference for intervention***


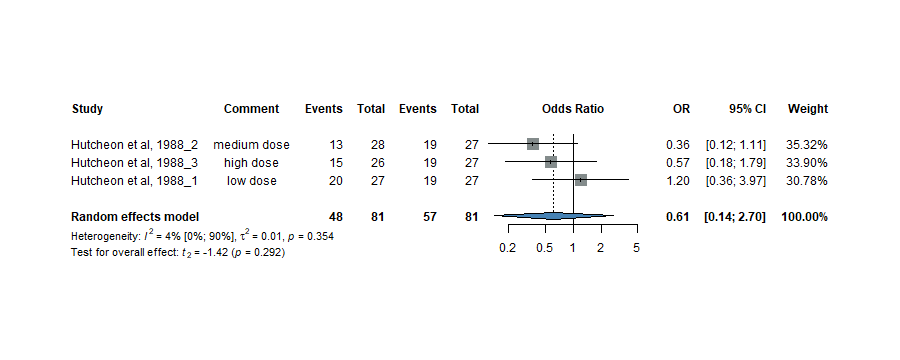


**Figure 35. Forest plots for PATIENT’S PREFERENCE FOR INTERVENTION versus Prochlorperazie treatment;** annotations: Tetrahydrocannabinol (THC), Cannabidiol (CBD), Sample Size (N), Standard Deviation (SD), Confidence Interval (CI), Odds Ratio (OR).

1. **Side effects**
2. ***Neurological side effects; any type of cannabinoids***

(includes confusion, concentration difficulties, coordination problems, dizziness)


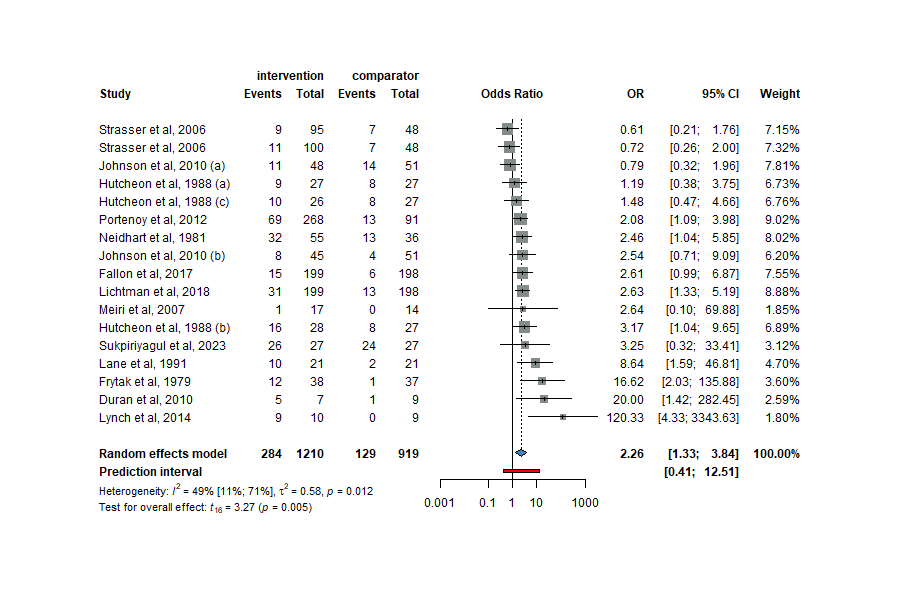


**Figure 36. Forest plots for the odds of NEUROLOGICAL SIDE EFFECTS when using any type of cannabinoids** annotations: Tetrahydrocannabinol (THC), Cannabidiol (CBD), Sample Size (N), Standard Deviation (SD), Confidence Interval (CI), Odds Ratio (OR).

1. ***Neurological side effects; THC-predominant drugs***

(includes confusion, concentration difficulties, coordination problems, dizziness)


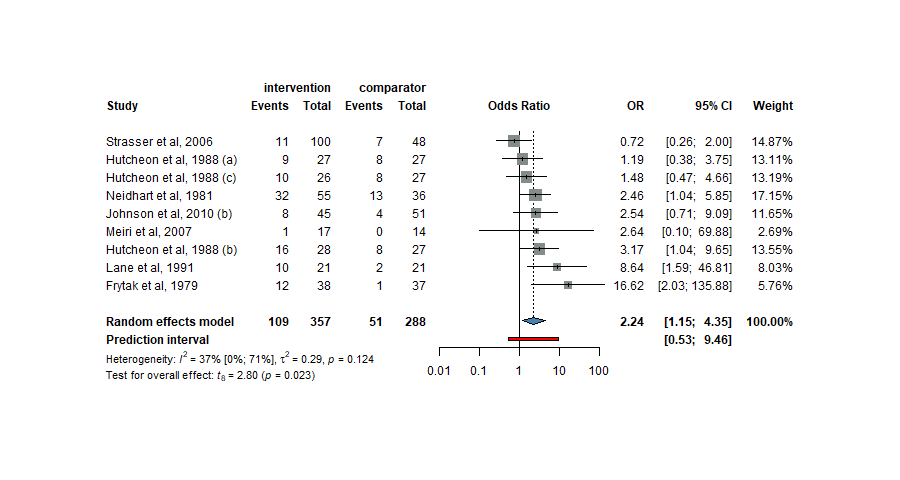


**Figure 37. Forest plots for the odds of NEUROLOGICAL SIDE EFFECTS when using THC-predomiant cannabinoids** annotations: Tetrahydrocannabinol (THC), Cannabidiol (CBD), Sample Size (N), Standard Deviation (SD), Confidence Interval (CI), Odds Ratio (OR).

1. ***Neurological side effects; Balanced THC:CBD drugs***

(includes confusion, concentration difficulties, coordination problems, dizziness)


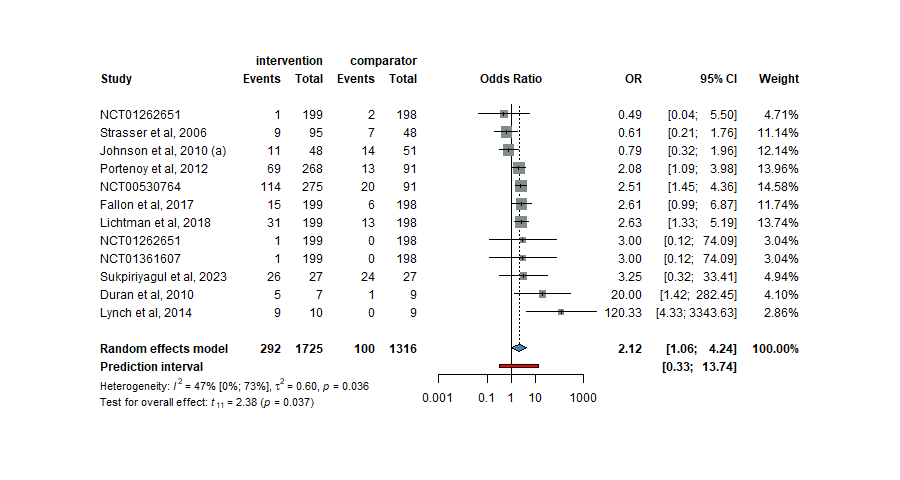


**Figure 38. Forest plots for the odds of NEUROLOGICAL SIDE EFFECTS when using balanced THC:CBD cannabinoids** annotations: Tetrahydrocannabinol (THC), Cannabidiol (CBD), Sample Size (N), Standard Deviation (SD), Confidence Interval (CI), Odds Ratio (OR).

1. ***Pain; any type of cannabinoids***


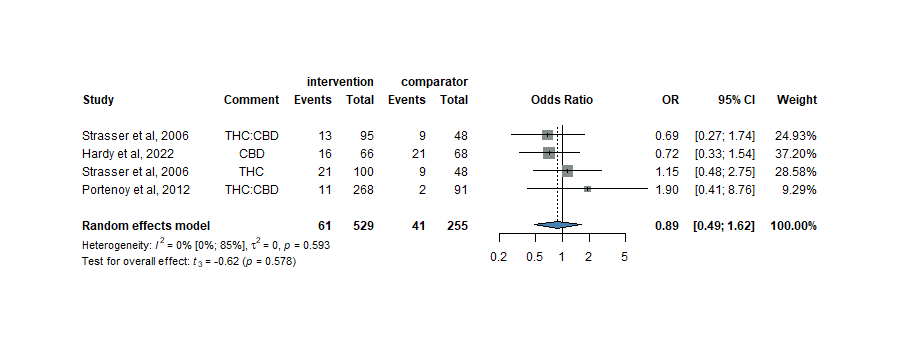


**Figure 39. Forest plots for the odds of PAIN, when using any type of cannabinoids** annotations: Tetrahydrocannabinol (THC), Cannabidiol (CBD), Sample Size (N), Standard Deviation (SD), Confidence Interval (CI), Odds Ratio (OR).

1. ***Psychiatric side effects; any type of cannabinoids***

(includes hallucinations, delusion, paranoia, psychosis, nightmares, anxiety, mood swings, paranoia)


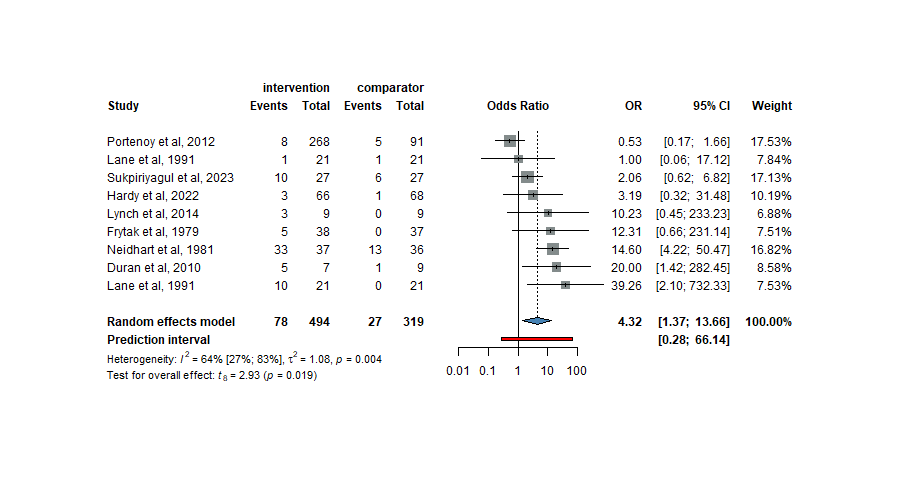


**Figure 40. Forest plots for the odds of PSYCHIATRIC SIDE EFFECTS, when using any type of cannabinoids** annotations: Tetrahydrocannabinol (THC), Cannabidiol (CBD), Sample Size (N), Standard Deviation (SD), Confidence Interval (CI), Odds Ratio (OR).

1. ***Psychiatric side effects; balanced THC:CBD drugs***

(includes hallucinations, delusion, paranoia, psychosis, nightmares, anxiety, mood swings, paranoia)


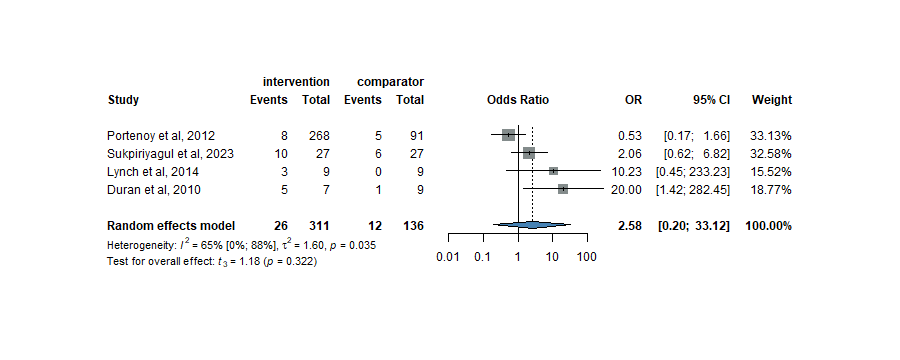


**Figure 41. Forest plots for the odds of PSYCHIATRIC SIDE EFFECTS, when using balanced THC:CBD cannabinoids** annotations: Tetrahydrocannabinol (THC), Cannabidiol (CBD), Sample Size (N), Standard Deviation (SD), Confidence Interval (CI), Odds Ratio (OR).

1. ***Psychiatric side effects; THC-predominant drugs***

(includes hallucinations, delusion, paranoia, psychosis, nightmares, anxiety, mood swings, paranoia)


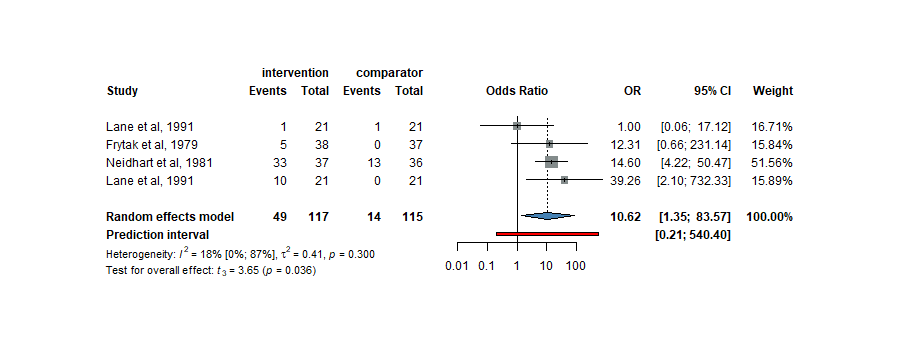


**Figure 42. Forest plots for the odds of PSYCHIATRIC SIDE EFFECTS, when using THC-predominant cannabinoids** annotations: Tetrahydrocannabinol (THC), Cannabidiol (CBD), Sample Size (N), Standard Deviation (SD), Confidence Interval (CI), Odds Ratio (OR).

1. ***Stoping the cannabinoid treatment due to side effects; any type of cannabinoids***


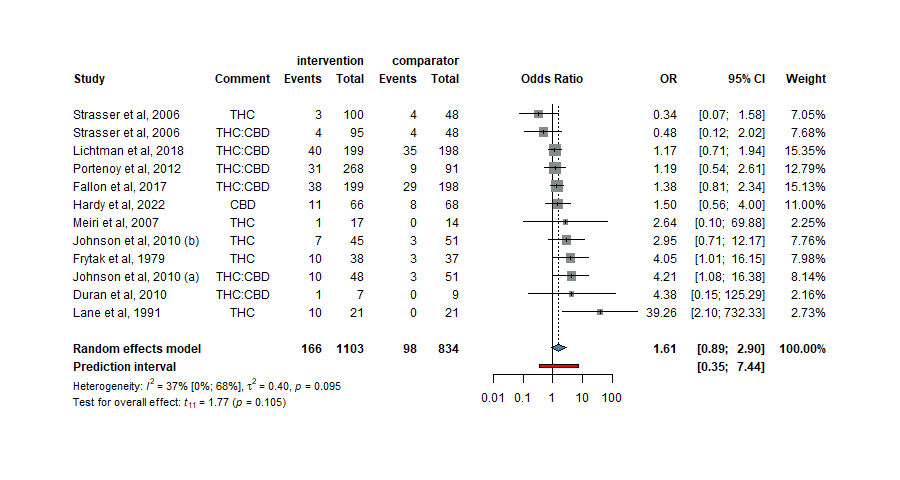


**Figure 43. Forest plots for the odds of STOPPING CANNABINOID TREATMENT DUE TO SIDE EFFECTS, any type of cannabinoids;** annotations: Tetrahydrocannabinol (THC), Cannabidiol (CBD), Sample Size (N), Standard Deviation (SD), Confidence Interval (CI), Odds Ratio (OR).

1. ***Stoping the cannabinoid treatment due to side effects; THC-predominant drugs***


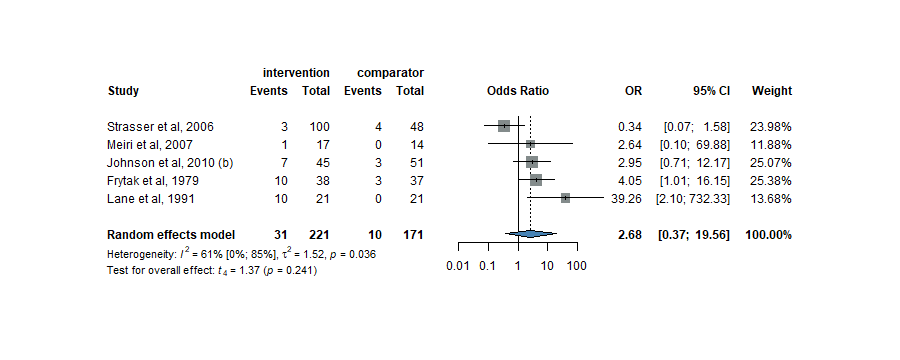


**Figure 44. Forest plots for the odds of STOPPING CANNABINOID TREATMENT DUE TO SIDE EFFECTS, THC-predominant drugs;** annotations: Tetrahydrocannabinol (THC), Cannabidiol (CBD), Sample Size (N), Standard Deviation (SD), Confidence Interval (CI), Odds Ratio (OR).

1. ***Stoping the cannabinoid treatment due to side effects; balanced THC:CBD drugs***


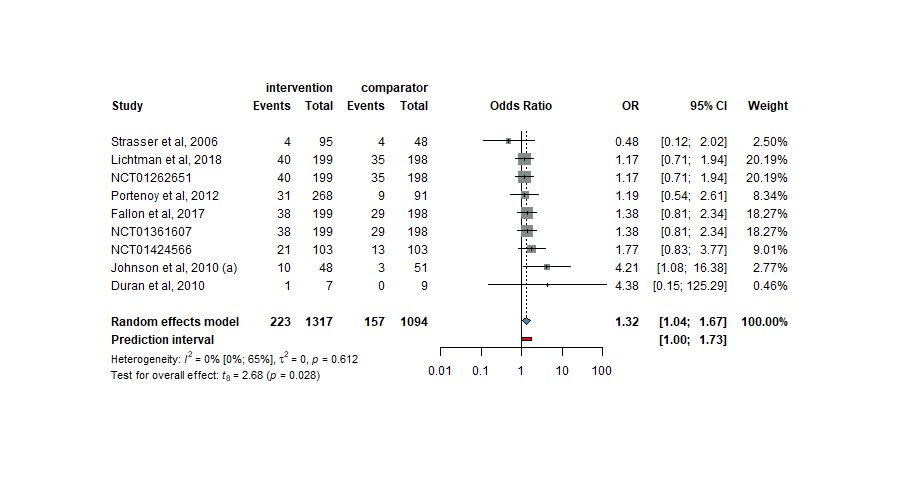


**Figure 45. Forest plots for the odds of STOPPING CANNABINOID TREATMENT DUE TO SIDE EFFECTS, balanced THC:CBD drugs;** annotations: Tetrahydrocannabinol (THC), Cannabidiol (CBD), Sample Size (N), Standard Deviation (SD), Confidence Interval (CI), Odds Ratio (OR).

1. ***Anorexia; any type of cannabinoid***

(includes appetite loss, anorexia, weight loss)


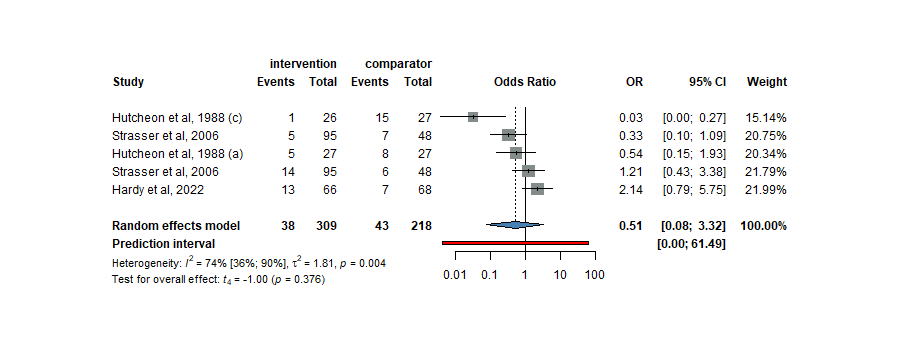


**Figure 46. Forest plots for the odds of ANOREXIA, when using any type of cannabinoids;** annotations: Tetrahydrocannabinol (THC), Cannabidiol (CBD), Sample Size (N), Standard Deviation (SD), Confidence Interval (CI), Odds Ratio (OR).

1. ***Asthenia; any type of cannabinoid***


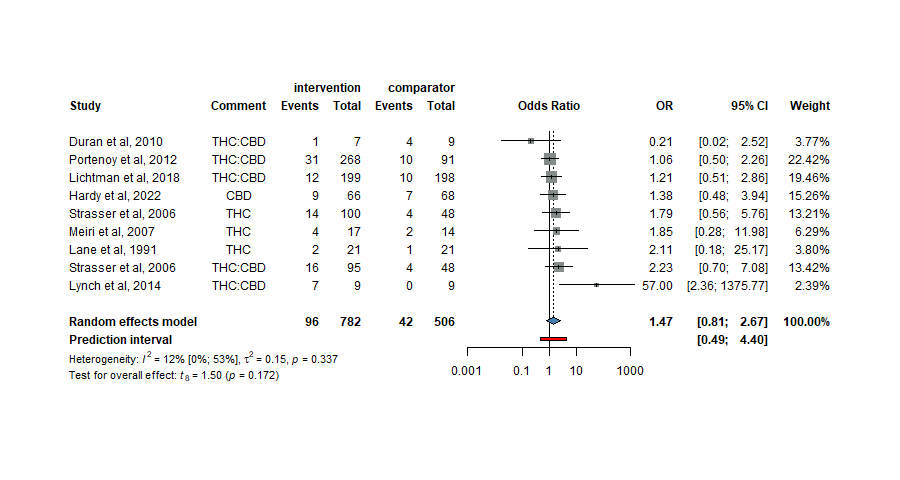


**Figure 47. Forest plots for the odds of ASTHENIA, when using any type of cannabinoids;** annotations: Tetrahydrocannabinol (THC), Cannabidiol (CBD), Sample Size (N), Standard Deviation (SD), Confidence Interval (CI), Odds Ratio (OR).

1. ***Asthenia; balanced THC:CBD drugs***


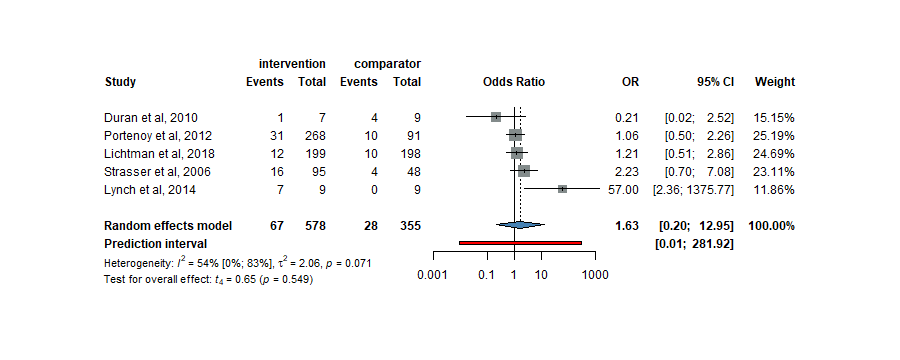


**Figure 47. Forest plots for the odds of ASTHENIA, when using balanced THC:CBD cannabinoids;** annotations: Tetrahydrocannabinol (THC), Cannabidiol (CBD), Sample Size (N), Standard Deviation (SD), Confidence Interval (CI), Odds Ratio (OR).

1. ***Asthenia; THC-predominant drugs***


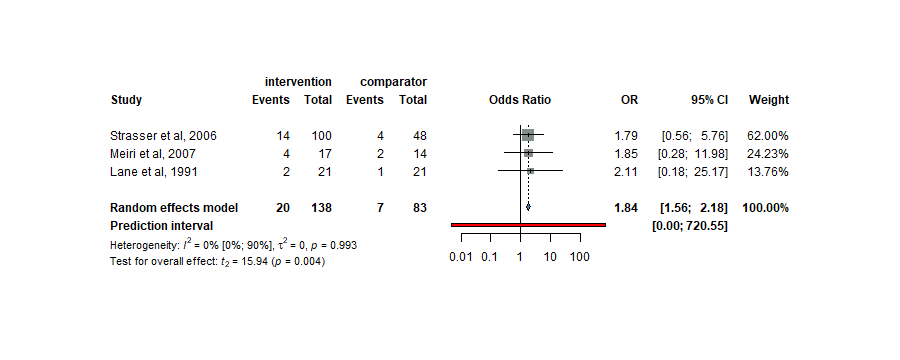


**Figure 48. Forest plots for the odds of ASTHENIA, when using THC-predominant cannabinoids;** annotations: Tetrahydrocannabinol (THC), Cannabidiol (CBD), Sample Size (N), Standard Deviation (SD), Confidence Interval (CI), Odds Ratio (OR).

1. ***Cardiovascular side effects; any type of cannabinoid***

(includes tachycardia, hypotension)


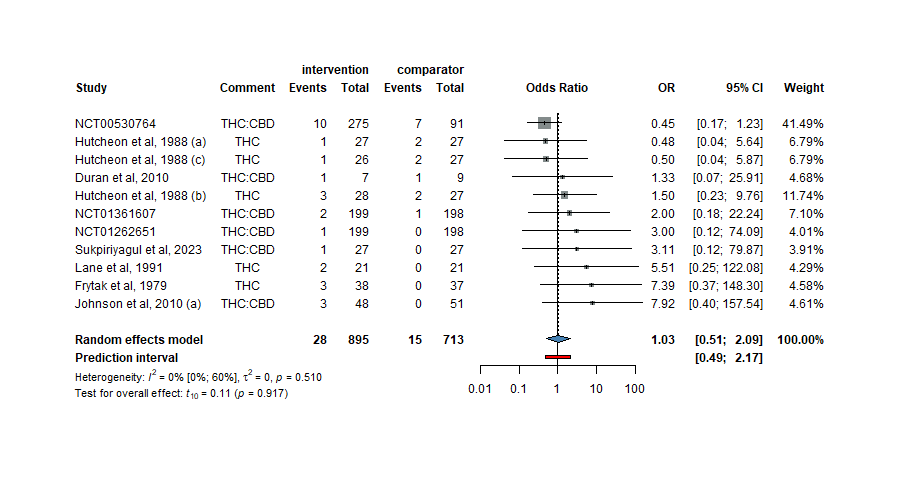


**Figure 49. Forest plots for the odds of CARDIOVASCULAR SIDE EFFECTS, when using any type of cannabinoids;** annotations: Tetrahydrocannabinol (THC), Cannabidiol (CBD), Sample Size (N), Standard Deviation (SD), Confidence Interval (CI), Odds Ratio (OR).

1. ***Cardiovascular side effects; balanced THC:CBD drugs***

(includes tachycardia, hypotension)


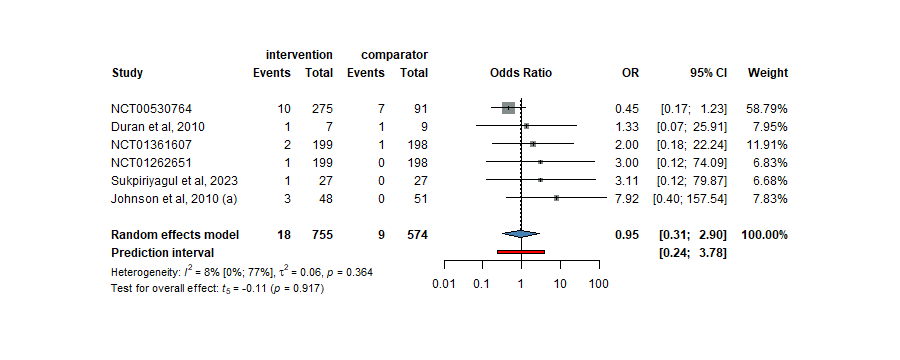


**Figure 50. Forest plots for the odds of CARDIOVASCULAR SIDE EFFECTS, when using balanced THC:CBD cannabinoids;** annotations: Tetrahydrocannabinol (THC), Cannabidiol (CBD), Sample Size (N), Standard Deviation (SD), Confidence Interval (CI), Odds Ratio (OR).

1. ***Cardiovascular side effects; THC-predominant drugs***


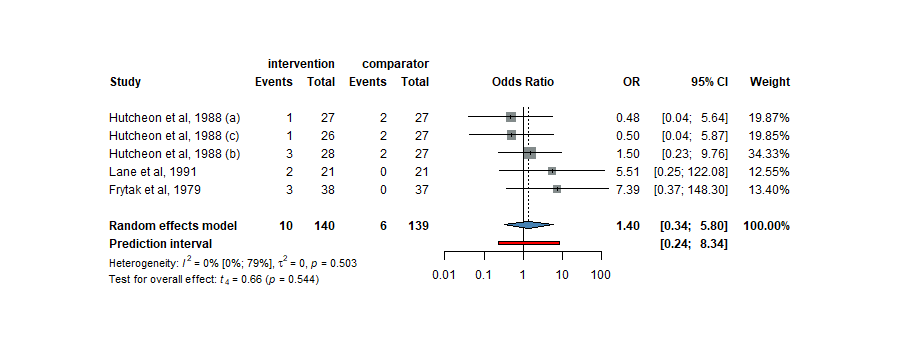


**Figure 51. Forest plots for the odds of CARDIOVASCULAR SIDE EFFECTS, when using THC-predominant cannabinoids;** annotations: Tetrahydrocannabinol (THC), Cannabidiol (CBD), Sample Size (N), Standard Deviation (SD), Confidence Interval (CI), Odds Ratio (OR).

1. ***Dyspnea; any type of cannabinoids***


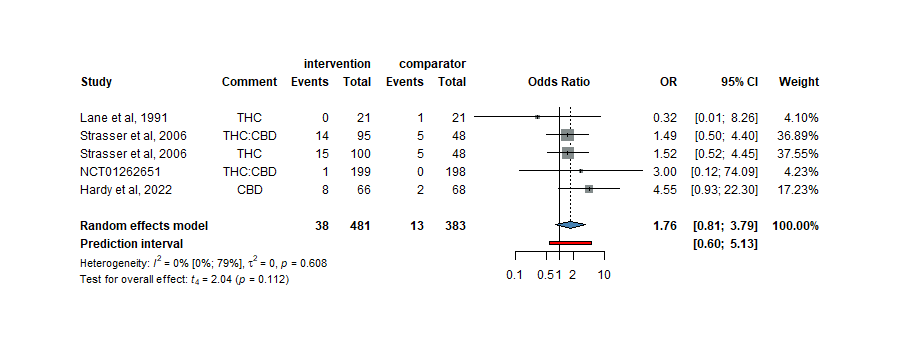


**Figure 52. Forest plots for the odds of DYSPNEA, when using any type of cannabinoids;** annotations: Tetrahydrocannabinol (THC), Cannabidiol (CBD), Sample Size (N), Standard Deviation (SD), Confidence Interval (CI), Odds Ratio (OR).

1. ***Gastrointestinal side effects; any type of cannabinoids***

(includes diarrhea, constipation, abdominal pain, nausea, vomiting, dry mouth, stomatitis, raised gamma-glutamil-transferase (GGT))


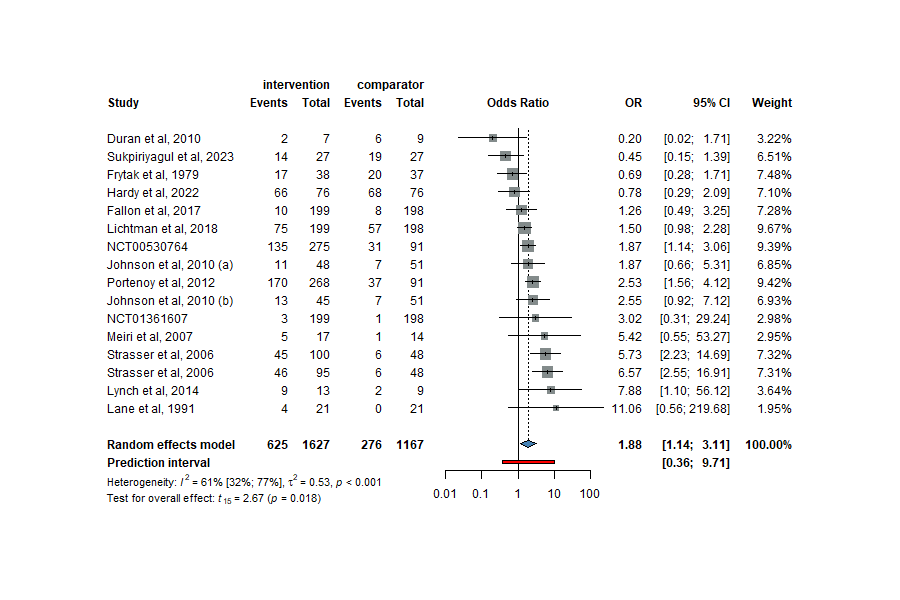


**Figure 53. Forest plots for the odds of GASTROINTESTINAL SIDE EFFECTS, when using any type of cannabinoids;** annotations: Tetrahydrocannabinol (THC), Cannabidiol (CBD), Sample Size (N), Standard Deviation (SD), Confidence Interval (CI), Odds Ratio (OR).

1. ***Gastrointestinal side effects; THC-predominant drugs***

(includes diarrhea, constipation, abdominal pain, nausea, vomiting, dry mouth, stomatitis, raised gamma-glutamil-transferase (GGT))


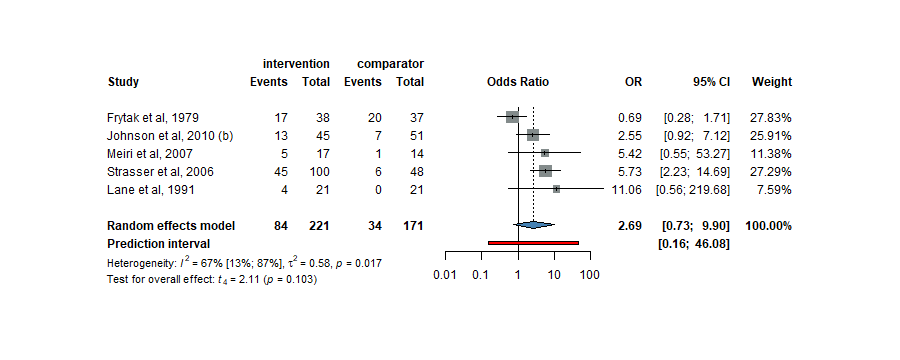


**Figure 54. Forest plots for the odds of GASTROINTESTINAL SIDE EFFECTS, when using THC-predominant cannabinoids;** annotations: Tetrahydrocannabinol (THC), Cannabidiol (CBD), Sample Size (N), Standard Deviation (SD), Confidence Interval (CI), Odds Ratio (OR).

1. ***Gastrointestinal side effects; balanced THC:CBD drugs***

(includes diarrhea, constipation, abdominal pain, nausea, vomiting, dry mouth, stomatitis, raised gamma-glutamil-transferase (GGT))


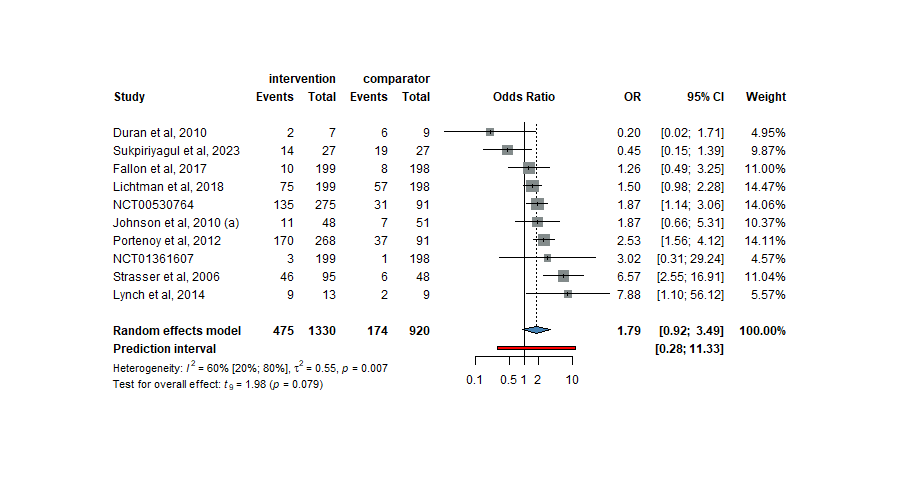


**Figure 55. Forest plots for the odds of GASTROINTESTINAL SIDE EFFECTS, when using balanced THC:CBD cannabinoids;** annotations: Tetrahydrocannabinol (THC), Cannabidiol (CBD), Sample Size (N), Standard Deviation (SD), Confidence Interval (CI), Odds Ratio (OR).

1. ***Hematological side effects, any type of cannaninoid***

(includes anemia)


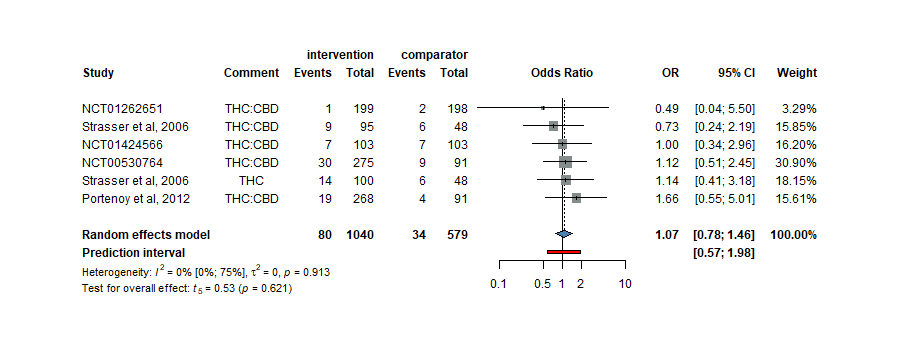


**Figure 56. Forest plots for the odds of HEMATOLOGICAL SIDE EFFECTS, when using any type of cannabinoids;** annotations: Tetrahydrocannabinol (THC), Cannabidiol (CBD), Sample Size (N), Standard Deviation (SD), Confidence Interval (CI), Odds Ratio (OR).

1. ***Neoplasm progression, Nabiximols***


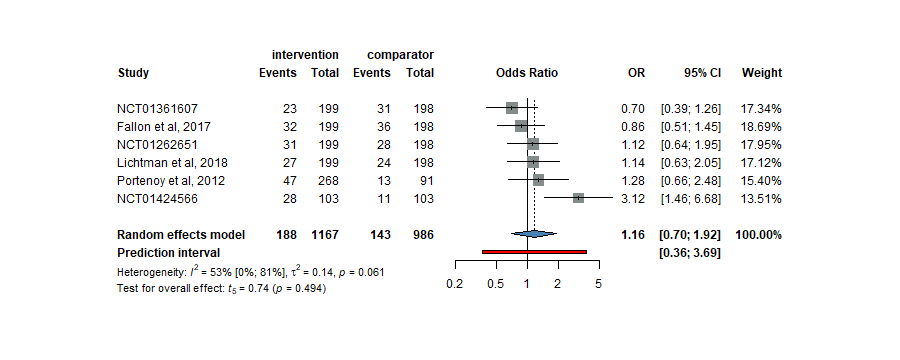


**Figure 57. Forest plots for the odds of NEOPLASM PROGRESSION, when using Nabiximols;** annotations: Tetrahydrocannabinol (THC), Cannabidiol (CBD), Sample Size (N), Standard Deviation (SD), Confidence Interval (CI), Odds Ratio (OR).

1. ***Somnolence, any type of cannabinoid***

(includes somnolence, drowsiness, sedation)


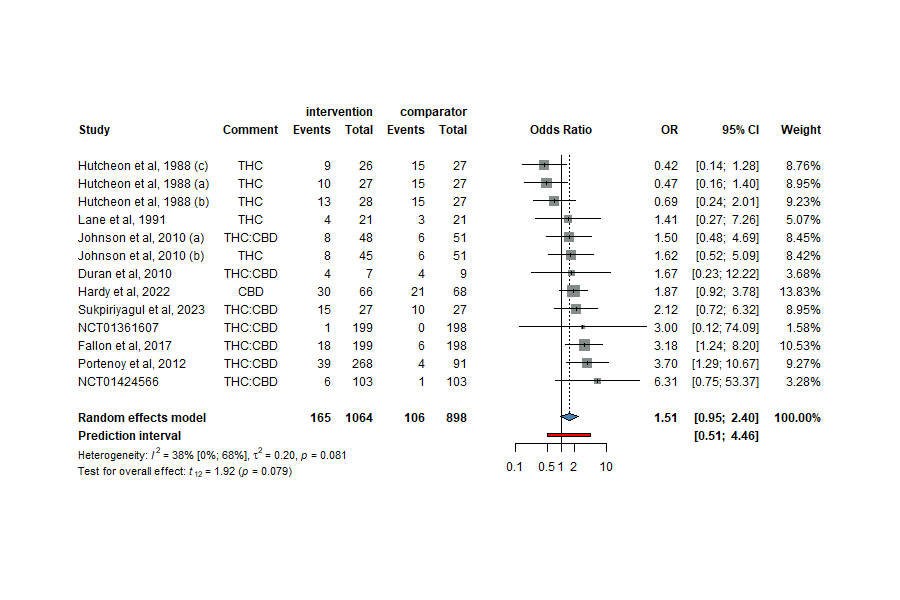


**Figure 58. Forest plots for the odds of SOMNOLENCE, when using any type of cannabinoids;** annotations: Tetrahydrocannabinol (THC), Cannabidiol (CBD), Sample Size (N), Standard Deviation (SD), Confidence Interval (CI), Odds Ratio (OR).

1. ***Somnolence, balanced THC:CBD drugs***

(includes somnolence, drowsiness, sedation)


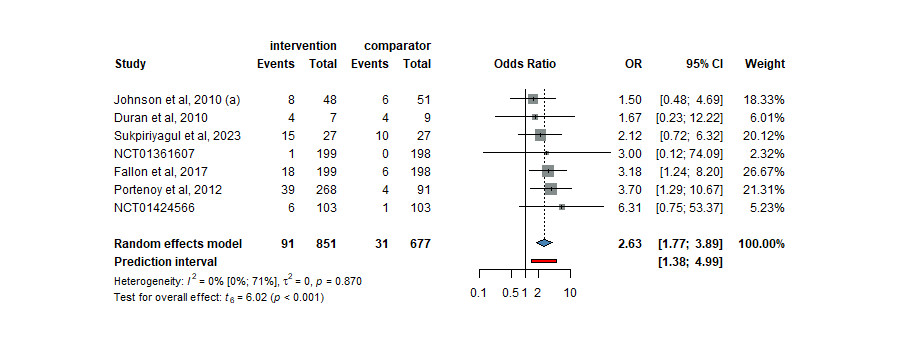


**Figure 59. Forest plots for the odds of SOMNOLENCE, when using balanced THC:CBD cannabinoids;** annotations: Tetrahydrocannabinol (THC), Cannabidiol (CBD), Sample Size (N), Standard Deviation (SD), Confidence Interval (CI), Odds Ratio (OR).

1. ***Somnolence, THC-predominant drugs***

(includes somnolence, drowsiness, sedation)


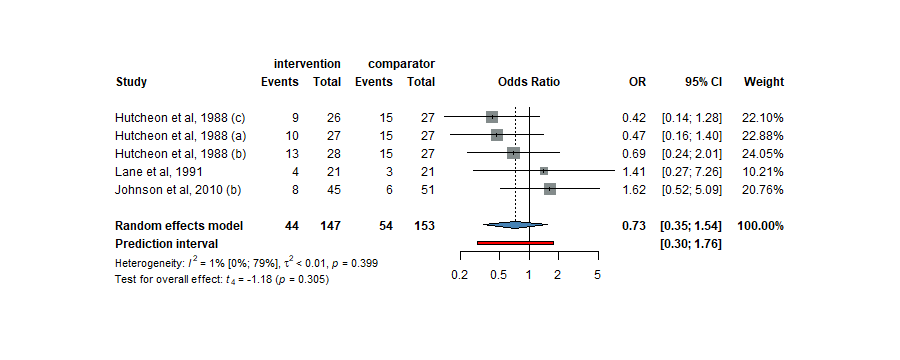


**Figure 60. Forest plots for the odds of SOMNOLENCE, when using THC-predominant cannabinoids;** annotations: Tetrahydrocannabinol (THC), Cannabidiol (CBD), Sample Size (N), Standard Deviation (SD), Confidence Interval (CI), Odds Ratio (OR).

**Table 4. Cannabinoid administration particularities**

| **Nr.** | **Author, Year** | **Type of cannabinoid** | **Concentration** | **Form of administration** | **Fixed dosage dosage/patient-titrated** | **Dosage/24h** |
| --- | --- | --- | --- | --- | --- | --- |
| 1 | Johnson et al, 2010 (a) | Balanced THC:CBD extract | 2.7mg:2.5mg/100µl | oromucosal spray | patient-titrated | 100-1100 µl |
| 2 | Johnson et al, 2010 (b) | THC-predominant extract | 2.7mg/100µl | oromucosal spray | patient-titrated | 100-1000 µl |
| 3 | Portenoy et al, 2012 | Nabiximols (balanced THC:CBD) | 2.7mg:2.5mg/100µl | oromucosal spray | patient-titrated | 100-1600 µl |
| 4 | Aviram et al, 2020 (a) | Medical cannabis (THC-predominant) | NR | mixed | patient-titrated | NA |
| 5 | Aviram et al, 2020 (b) | Medical cannabis (CBD-predominant) | NR | mixed | patient-titrated | NA |
| 6 | Aviram et al, 2020 (c) | Medical cannabis (balanced THC:CBD) | NR | mixed | patient-titrated | NA |
| 7 | Chang et al, 2019 | THC-predominant | NR | mixed | patient-titrated | NA |
| 8 | Clarke et al, 2022 | Balanced THC:CBD nanoparticle | 1.25 mg:1.25mg/100µl | oromucosal spay | fixed dosage | 300-3600 µl |
| 9 | Cone et al, 1982 | Nabilone (THC-predominant) | 1 mg | capsule | fixed dosage | 2-4 mg |
| 10 | Cronin et al, 1981 | Levonantradol (THC-predominant) | 0.5 mg | intramuscular | fixed dosage | 3 mg |
| 11 | Awofisayo et al, 2021 | CBD-predominant | NR | oral oil | patient titrated | NA |
| 12 | Fallon et al, 2017 | Sativex (Balanced THC:CBD) | 2.7mg:2.5mg/100µl | oromucosal spray | patient-titrated | 100-1000 µl |
| 13 | Frytak et al, 1979 | THC-predominant | 15 mg | capsule | fixed dosage | 45 mg |
| 14 | Good et al, 2020 (a) | THC -predominant | 10 mg/ml | oral oil | patient-titrated | 2.5-30 mg |
| 15 | Good et al, 2020 (b) | CBD-predominant | 100 mg/ml | oral oil | patient-titrated | 50-600 mg |
| 16 | Gulbransen et al, 2020 | CBD-predominant | NR | oral oil | patient-titrated | 40-300 mg |
| 17 | Hardy et al, 2022 | CBD-predominant | 100 mg/ml | oral oil | patient-titrated | 50 mg-200 mg |
| 18 | Zylla et al, 2021 | Medical Cannabis (Balanced THC:CBD) | 2.5-5 mg:2.5-5 mg | mixed | patient-titrated | 40 mg:40 mg |
| 19 | Zhang et al, 2018 | Marijuana (Balanced THC:CBD) | NR | mixed | patient-titrated | NA |
| 20 | Wongkongdech et al, 2022 (a) | Medical Cannabis (THC-predominant) | 12.5 mg | mixed | patient-titrated | NA |
| 21 | Wongkongdech et al, 2022 (b) | DTAM Ganja Oil (Balanced THC:CBD) | 2 mg : 0.5 mg | mixed | patient-titrated | NA |
| 22 | Wongkongdech et al, 2022 (c) | Medical Cannabis (Balanced THC:CBD) | 27 mg : 25 mg | mixed | patient-titrated | NA |
| 23 | Welsh et al, 1983 | Levonantradol (THC-predominant) | 0.25 mg-1 mg | capsule | fixed dosage | 1.5 mg-6 mg |
| 24 | Waissengrin et al, 2015 | Medical Cannabis (Balanced THC:CBD) | NR | NR | patient titrated | NA |
| 25 | Turcott et al, 2018 | Nabilone (THC-predominant) | 0.5 mg | capsule | fixed dosage | 0.5 mg-1 mg |
| 26 | Sukpiriyagul et al, 2023 | Balanced THC:CBD extract | 2.7 mg:2.5 mg/ml | oral oil | fixed dosage | 8.1 mg-7.5 mg |
| 27 | Strasser et al, 2006 (a) | Balanced THC:CBD extract | 2.5mg:1mg | capsule | fixed dosage | 5mg:2mg |
| 28 | Strasser et al, 2006 (b) | THC-predominant | 2.5mg | capsule | fixed dosage | 5mg |
| 29 | Stambaugh et al, 1984 | Levonantradol (THC-predominant) | 0.5 mg | intramuscular | fixed dosage | 0.5 mg-3 mg |
| 30 | Pitchard et al, 2019 | Cannabis (Balanced THC:CBD) | NR | mixed | patient-titrated | NA |
| 31 | Lichtman et al, 2018 | Nabiximols (Balanced THC:CBD) | 2.7mg:2.5mg/100µl | oromucosal spray | patient-titrated | 100-1000 µl |
| 32 | Aprikian et al, 2023 (a) | Medical cannabis (THC-predominant) | NR | mixed | patient-titrated | NA |
| 33 | Aprikian et al, 2023 (b) | Medical cannabis (Balanced THC:CBD) | NR | mixed | patient-titrated | NA |
| 34 | Aprikian et al, 2023 (c) | Medical cannabis (CBD-predominant) | NR | mixed | patient-titrated | NA |
| 35 | Nathan et al, 2023 | Medical Cannabis (Balanced THC:CBD) | NR | mixed | patient-titrated | NA |
| 36 | Schloss et al, 2021 | Medical cannabis (Balanced THC:CBD) | 4.6mg:4.8mg/ml and 15mg:3.8mg/ml) | mixed | patient-titrated | 10.35-27 mg: 10.8- 6.8 mg |
| 37 | Duran et al, 2010 | Whole plant cannabis-based medicine (Balanced THC:CBD) | 2.7mg:2.5mg/100µl | oromucosal spray | patient-titrated | 100-3200 µl |
| 38 | Lynch et al, 2014 | Nabiximols (Balanced THC:CBD) | 2.7mg:2.5mg/100µl | oromucosal spray | patient-titrated | 100-1200 µl |
| 39 | Meiri et al, 2007 | Dronabinol (THC-predominant) | 2.5 mg | capsule | patient-titrated | 20 mg |
| 40 | Bar-Sela et al, 2013 | Medical cannabis (Balanced THC:CBD) | NR | mixed | patient-titrated | NA |
| 41 | Chang A. et al, 1979 | THC-predominant | 10mg/m2 | mixed | fixed dosage | 50 mg/m2 |
| 42 | Chang A. et al, 1981 | THC-predominant | 10mg/m3 | mixed | fixed dosage | 51 mg/m2 |
| 43 | Côté et al, 2016 | Nabilone (THC-predominant) | 0.5 mg | capsule | fixed dosage | 0.5mg-2mg |
| 44 | Anderson et al, 2019 | Medical cannabis (Balanced THC:CBD) | NR | mixed | patient-titrated | NA |
| 45 | Donovan et al, 2019 | THC-predominant | NR | mixed | patient-titrated | NA |
| 46 | Schleidera et al, 2018 | Medical cannabis (Balanced THC:CBD) | NR | mixed | patient-titrated | NA |
| 47 | Lane et al, 1991 | Dronabinol (THC-predominant) | 10 mg | capsule | fixed dosage | 40 mg |
| 48 | Neidhart et al, 1981 | THC-predominant | 10 mg | capsule | fixed dosage | 80 mg |
| 49 | Hutcheon et al, 1988 | Levonantradol (THC-predominant) | 0.5-1 mg | intramuscular | fixed dosage | 2-4 mg |
| 50 | Ahmedzai et al, 1983 | Nabilone (THC-predominant) | 1 mg | capsule | fixed dosage | 4 mg |
| 51 | Chan et al, 1986 | Nabilone (THC-predominant) | 1 mg | capsule | fixed dosage | 4 mg |
| 52 | Crawford et al, 1986 | Nabilone (THC-predominant) | 1 mg | capsule | fixed dosage | 2 mg |
| 53 | Cunningham et al, 1988 | Nabilone (THC-predominant) and Prochlorperazine | 2mg +5mg | capsule | fixed dosage | 4mg+10mg |
| 54 | Dalzell et al, 1986 | Nabilone (THC-predominant) | 0.5 mg | capsule | fixed dosage | 3 mg |
| 55 | Einhorn et al, 1982 (a) | Nabilone (THC-predominant) | 2 mg | capsule | fixed dosage | 4-8 mg |
| 56 | Einhorn et al, 1981 | Nabilone (THC-predominant) | 2 mg | capsule | fixed dosage | 8 mg |
| 57 | Gerhartz et al, 1983 (a) | Levonantradol (THC-predominant) | 0.5 mg | intramuscular | fixed dosage | 2 mg |
| 58 | Grimison et al, 2020 | Balanced THC:CBD extract | 2.5mg:2.5mg | capsule | patient-titrated | 10mg:10mg |
| 59 | Herman et al, 1979 | Nabilone (THC-predominant) | 1 mg | capsule | fixed dosage | 6 mg |
| 60 | Johansson et al, 1982 | Nabilone (THC-predominant) | 2 mg | capsule | fixed dosage | 4 mg |
| 61 | Jones et al, 1982 | Nabilone (THC-predominant) | 2 mg | capsule | fixed dosage | 4 mg |
| 62 | Wada et al, 1982 | -predominant | 2 mg | capsule | fixed dosage | 4 mg |
| 63 | Underleider et al, 1985 | Compazine (THC-predominant) | 7.5 mg for body surface area<1.4 m2; 10 mg for body surface area 1.4 m2- 1.8 m2; 12.5 mg for body surface area >1.8 m2 | capsule | fixed dosage | 30-50 mg |
| 64 | Sallan et al, 1975 | THC-predominant | 15 mg-20 mg | capsule | fixed dosage | 45mg - 60 mg |
| 65 | Orr et al, 1980 (a) | THC-predominant | 7 mg/m2 | capsule | fixed dosage | 42 mg/ m2 |
| 66 | Niiranen et al, 1985 | Nabilone (THC-predominant) | 1 mg | oral | fixed dosage | 2 mg |
| 67 | Heim et al, 1984 | Levonantradol (THC-predominant) | 0.5 mg | im | fixed dosage | 1mg |
| 68 | Levitt et al, 1982 | Nabilone (THC-predominant) | NR | NR | NR | NA |
| 69 | Mccabe et al, 1988 | THC-predominant | 15mg/m2 | capsule | fixed dosage | 90mg/m2 |
| 70 | Jatoi et al, 2002 | Dronabinol (THC-predominant) | 2.5 mg | capsule | fixed dosage | 5 mg |
| 71 | Lucraft et al, 1982 | Levonantradol (THC-predominant) | 0.5 mg- 0.75 mg | NR | fixed dosage | 0.5 mg- 0.75 mg |
| 72 | Dominikak et al, 2023 | CBD-predominant | 150 mg | oral | fixed dosage | 300 mg |
| 73 | Heim et al, 1982 | Levonantradol (THC-predominant) | 1 mg | intramuscular | fixed dosage | 3 mg |
| 74 | Neiderle et al, 1986 | Nabilone (THC-predominant) | 2 mg | capsule | fixed dosage | 4 mg |
| 75 | Kasvis et al, 2022 | Medical Cannabis (THC-predominant, Balanced THC:CBD, CBD-predominant) | NR | mixed | patient-titrated | mixed |
| 76 | Davies et al, 1974 | THC-predominant | 10 mg | oral | fixed dosage | 10 mg |
| 77 | Brisbois et al, 2011 | Dronabinol (THC-predominant) | 2.5 mg | capsule | fixed dosage | 5 mg |
| 78 | Eliott et al, 2016 | Medical cannabis (Balanced THC:CBD) | NR | NR | NR | NA |
| 79 | Diasio et al, 1981 | Levonantradol (THC-predominant) | 0.5 mg-1 mg | NR | fixed dosage | 70 mg |
| 80 | Aviram et al, 2022 | Medical cannabis (Balanced THC:CBD) | NR | NR | NR | NA |
| 81 | Bar Sela et al, 2018 | Medical cannabis (Balanced THC:CBD) | NR | NR | NR | 30 mg |
| 82 | Elder et al, 2015 | Dronabinol (THC-predominant) | NR | NR | NR | NA |
| 83 | Abrahamov et al, 1995 | Delta 8-THC-predominant | 18 mg/m2 | oral | patient-titrated | 90 mg/m2 |
| 84 | Lee et al, 2023 | Medical cannabis (Balanced THC:CBD) | NR | NR | NR | NA |
| 85 | Laszlo et al, 1961 | Levonantradol (THC-predominant) | 0.5 mg- 2 mg | oral | fixed dosage | 14 mg |
| 86 | Joss et al, 1982 | Levonantradol (THC-predominant) | NR | NR | NR | NA |
| 87 | Johnson et al 2013 (a) | Balanced THC:CBD extract | 2.7mg:2.5mg/100µl | oromucosal spray | patient-titrated | 480 µl |
| 88 | Johnson et al 2013 (b) | THC-predominant extract | 2.7mg/100µl | oromucosal spray | patient-titrated | 481 µl |
| 89 | Lucas et al, 1980 | THC-predominant | 5 mg- 15 mg/m2 | oral | fixed dosage | 75 mg |
| 90 | Macari et al, 2020 | Medical cannabis (Balanced THC:CBD) | NR | NR | NR | NA |
| 91 | Niiranen et al, 1987 | Nabilone (THC-predominant) | 2 mg | oral | fixed dosage | 4 mg |
| 92 | Meghani et al, 2021 | CBD-predominant | NR | NR | NR | NA |
| 93 | Maida et al, 2008 | Nabilone (THC-predominant) | NR | NR | NR | NA |
| 94 | Pasawarat et al, 2020 | Medical cannabis (Balanced THC:CBD) | NR | NR | NR | NA |
| 95 | McClure et al, 2023 | Medical cannabis (Balanced THC:CBD) | NR | NR | NR | NA |
| 96 | Nielsen et al, 2022 | Medical cannabis (Balanced THC:CBD) | NR | NR | NR | NA |
| 97 | Nelson et al, 1994 | THC-predominant | 2.5 mg | oral | fixed dosage | 5 mg- 7.5 mg |
| 98 | Polito et al, 2018 | Nabilone (THC-predominant) | NR | NR | NR | NA |
| 99 | Pomeroy et al, 1986 | Nabilone (THC-predominant) | 1 mg | oral | fixed dosage | 3 mg |
| 100 | Saadeh et al, 2018 | Medical cannabis (Balanced THC:CBD) | NR | NR | NR | NA |
| 101 | Underleider et al, 1982 | THC-predominant | 7.5 mg for body surface area<1.4 m2; 10 mg for body surface area 1.4 m2- 1.8 m2; 12.5 mg for body surface area >1.8 m2 | capsule | fixed dosage | 30-50 mg |
| 102 | Scheidler et al, 1984 | Levonantradol (THC-predominant) | 1 mg | intramuscular | fixed dosage | 4 mg |
| 103 | Zutt et al, 2006 | Dronabinol (THC-predominant) | 2.5 | oral | fixed dosage | 5 mg |
| 104 | Zaki et al, 2017 | Medical cannabis (Balanced THC:CBD) | NR | NR | NR | NA |
| 105 | Waissengrin et al, 2021 | Medical cannabis (Balanced THC:CBD) | NR | NR | NR | NA |
| 106 | Sweet et al, 1981 | THC-predominant | 5 mg/m2 | oral | fixed dosage | 15 mg/m2 |

NR=not reported, NA=not applicable, THC=tetrahydrocannabinol, CBD=cannabidiol

**Table 5. GRADE assessment**

| **Certainty assessment** | | | | | | | **№ of patients** | | **Effect** | | **Certainty** | **Importance** |
| --- | --- | --- | --- | --- | --- | --- | --- | --- | --- | --- | --- | --- |
| **№ of studies** | **Study design** | **Risk of bias** | **Inconsistency** | **Indirectness** | **Imprecision** | **Other considerations** | **cannabinoids** | **placebo** | **Relative (95% CI)** | **Absolute (95% CI)** |  |  |
| **Quality of life (assessed with: NRS; Scale from: 0 to 10)** | | | | | | | | | | | | |
| 8 | randomised trials | very serious | not serious | serious^a^ | not serious | none | 559 | 512 | - | MD **0.16 points more** (0.02 fewer to 0.35 more) | ⨁◯◯◯ Very low^a^ | CRITICAL |
| **Pain (assessed with: NRS scale; Scale from: 0 to 10)** | | | | | | | | | | | | |
| 7 | randomised trials | serious | serious^b^ | serious^a^ | not serious | none | 592 | 632 | - | SMD **0.1 SD lower** (0.58 lower to 0.39 higher) | ⨁◯◯◯ Very low^a,b^ | CRITICAL |
| **Appetite (assessed with: NRS; Scale from: 0 to 10)** | | | | | | | | | | | | |
| 7 | randomised trials | serious | very serious^c^ | serious | not serious | none | 331 | 290 | - | SMD **0.46 SD higher** (0.29 lower to 1.2 higher) | ⨁◯◯◯ Very low^c^ | CRITICAL |
| **Constipation (assessed with: NRS; Scale from: 0 to 10)** | | | | | | | | | | | | |
| 5 | randomised trials | serious | serious^b^ | not serious | not serious | none | 454 | 452 | - | MD **0.19 points lower** (0.68 lower to 0.3 higher) | ⨁⨁◯◯ Low^b^ | IMPORTANT |
| **Opioid consumption (Daily maintenance opioid intake) (assessed with: mg ; Scale from: 0 to indefinite)** | | | | | | | | | | | | |
| 5 | randomised trials | serious | serious^b^ | not serious | very serious^d^ | none | 414 | 452 | - | MD **0.09 mg higher** (9.95 lower to 10.13 higher) | ⨁◯◯◯ Very low^b,d^ | IMPORTANT |
| **Depression (assessed with: NRS; Scale from: 0 to 10)** | | | | | | | | | | | | |
| 3 | randomised trials | serious | very serious^c^ | serious^a^ | not serious | none | 383 | 389 | - | MD **0.6 points higher** (0.65 lower to 1.86 higher) | ⨁◯◯◯ Very low^a,c^ | IMPORTANT |
| **Fatigue (assessed with: NRS; Scale from: 0 to 10)** | | | | | | | | | | | | |
| 5 | randomised trials | very serious | very serious^c^ | serious^a^ | serious^d^ | none | 228 | 237 | - | MD **0.3 points higher** (1.24 lower to 1.83 higher) | ⨁◯◯◯ Very low^a,c,d^ | IMPORTANT |
| **Mobility (assessed with: NRS; Scale from: 0 to 10)** | | | | | | | | | | | | |
| 6 | randomised trials | very serious | serious^b^ | serious^a^ | not serious | none | 237 | 246 | - | MD **0.05 points lower** (0.42 lower to 0.32 higher) | ⨁◯◯◯ Very low^a,b^ | IMPORTANT |
| **Nausea and vomiting (assessed with: Complete response)** | | | | | | | | | | | | |
| 6 | randomised trials | very serious | serious^b^ | serious^a,e^ | very serious^d^ | none | 139/272 (51.1%) | 388/774 (50.1%) | **OR 2.18** (0.79 to 6.00) | **185 more per 1,000** (from 59 fewer to 356 more) | ⨁◯◯◯ Very low^a,b,d,e^ | CRITICAL |
| **Nausea (assessed with: NRS; Scale from: 0 to 10)** | | | | | | | | | | | | |
| 6 | randomised trials | very serious | very serious^b^ | serious^a^ | not serious | none | 252 | 264 | - | MD **0.08 points higher** (0.49 lower to 0.65 higher) | ⨁◯◯◯ Very low^a,b^ | IMPORTANT |
| **Insomnia (assessed with: NRS; Scale from: 0 to 10)** | | | | | | | | | | | | |
| 6 | randomised trials | very serious | not serious | not serious | not serious | none | 648 | 693 | - | MD **0.27 points lower** (0.47 lower to 0.08 lower) | ⨁⨁◯◯ Low | IMPORTANT |
| **Nausea (assessed with: NRS; Scale from: 0 to 10)** | | | | | | | | | | | | |
| 4 | non-randomised studies | serious | serious^b^ | very serious^a,e^ | not serious | none | 932 |  | - | MD **0.75 lower** (1.8 lower to 0.3 higher) | ⨁◯◯◯ Very low^a,b,e^ | CRITICAL |
| **Appetite loss (assessed with: NRS; Scale from: 0 to 10)** | | | | | | | | | | | | |
| 3 | non-randomised studies | serious | very serious^c^ | very serious^a,e^ | very serious^d^ | none | 849 |  | - | MD **1.88 lower** (6.23 lower to 2.46 higher) | ⨁◯◯◯ Very low^a,c,d,e^ | CRITICAL |
| **Anxiety (assessed with: NRS; Scale from: 0 to 10)** | | | | | | | | | | | | |
| 9 | non-randomised studies | very serious | very serious^c^ | very serious^a,e^ | serious^d^ | none | 1039 |  | - | MD **1.03 lower** (2.22 lower to 0.39 lower) | ⨁◯◯◯ Very low^a,c,d,e^ | CRITICAL |
| **Quality of life (assessed with: NRS; Scale from: 0 to 10)** | | | | | | | | | | | | |
| 5 | non-randomised studies | very serious | very serious^c^ | very serious^a,e^ | very serious^d^ | none | 1387 |  | - | MD **0.73 higher** (1.17 lower to 2.64 higher) | ⨁◯◯◯ Very low^a,c,d,e^ | CRITICAL |
| **Pain (assessed with: NRS; Scale from: 0 to 10)** | | | | | | | | | | | | |
| 16 | non-randomised studies | very serious | very serious^c^ | very serious^a,e^ | not serious | none | 2591 |  | - | MD **1.22 lower** (1.92 lower to 0.52 lower) | ⨁◯◯◯ Very low^a,c,e^ | CRITICAL |
| **Insomnia (assessed with: NRS; Scale from: 0 to 10)** | | | | | | | | | | | | |
| 5 | non-randomised studies | serious | very serious^c^ | very serious^a,e^ | not serious | none | 940 |  | - | MD **1.08 lower** (2.48 lower to 0.33 higher) | ⨁◯◯◯ Very low^a,c,e^ | CRITICAL |

**CI:** confidence interval; **MD:** mean difference; **OR:** odds ratio; **SMD:** standardised mean difference

Explanations

a. Different THC/CBD content

b. Moderate heterogeneity

c. High heterogeneity

d. High CI range

e. Differences in population

**Table 6. Summary of findings table**

| Outcomes | **Anticipated absolute effects^*^** (95% CI) | | Relative effect (95% CI) | № of participants (studies) | Certainty of the evidence (GRADE) | Comments |
| --- | --- | --- | --- | --- | --- | --- |
|  | **Risk with placebo** | **Risk with cannabinoids** |  |  |  |  |
| Quality of life assessed with: NRS Scale from: 0 to 10 | The mean quality of life was **0** points | MD **0.16 points more** (0.02 fewer to 0.35 more) | - | 1071 (8 RCTs) | ⨁◯◯◯ Very low^a^ |  |
| Pain assessed with: NRS scale Scale from: 0 to 10 | - | SMD **0.1 SD lower** (0.58 lower to 0.39 higher) | - | 1224 (7 RCTs) | ⨁◯◯◯ Very low^a,b^ |  |
| Appetite assessed with: NRS Scale from: 0 to 10 | - | SMD **0.46 SD higher** (0.29 lower to 1.2 higher) | - | 621 (7 RCTs) | ⨁◯◯◯ Very low^c^ |  |
| Constipation assessed with: NRS Scale from: 0 to 10 | The mean constipation was **0** points | MD **0.19 points lower** (0.68 lower to 0.3 higher) | - | 906 (5 RCTs) | ⨁⨁◯◯ Low^b^ |  |
| Opioid consumption (Daily maintenance opioid intake) assessed with: mg  Scale from: 0 to indefinite | The mean opioid consumption (Daily maintenance opioid intake) was **0** mg | MD **0.09 mg higher** (9.95 lower to 10.13 higher) | - | 866 (5 RCTs) | ⨁◯◯◯ Very low^b,d^ |  |
| Depression assessed with: NRS Scale from: 0 to 10 | The mean depression was **0** points | MD **0.6 points higher** (0.65 lower to 1.86 higher) | - | 772 (3 RCTs) | ⨁◯◯◯ Very low^a,c^ |  |
| Fatigue assessed with: NRS Scale from: 0 to 10 | The mean fatigue was **0** points | MD **0.3 points higher** (1.24 lower to 1.83 higher) | - | 465 (5 RCTs) | ⨁◯◯◯ Very low^a,c,d^ |  |
| Mobility assessed with: NRS Scale from: 0 to 10 | The mean mobility was **0** points | MD **0.05 points lower** (0.42 lower to 0.32 higher) | - | 483 (6 RCTs) | ⨁◯◯◯ Very low^a,b^ |  |
| Nausea and vomiting assessed with: Complete response | 501 per 1,000 | **687 per 1,000** (443 to 858) | **OR 2.18** (0.79 to 6.00) | 1046 (6 RCTs) | ⨁◯◯◯ Very low^a,b,d,e^ |  |
| Nausea assessed with: NRS Scale from: 0 to 10 | The mean nausea was **0** points | MD **0.08 points higher** (0.49 lower to 0.65 higher) | - | 516 (6 RCTs) | ⨁◯◯◯ Very low^a,b^ |  |
| Insomnia assessed with: NRS Scale from: 0 to 10 | The mean insomnia was **0** points | MD **0.27 points lower** (0.47 lower to 0.08 lower) | - | 1341 (6 RCTs) | ⨁⨁◯◯ Low |  |
| Nausea assessed with: NRS Scale from: 0 to 10 | The mean nausea was **0** | MD **0.75 lower** (1.8 lower to 0.3 higher) | - | 932 (4 non-randomised studies) | ⨁◯◯◯ Very low^a,b,e^ |  |
| Appetite loss assessed with: NRS Scale from: 0 to 10 | The mean appetite loss was **0** | MD **1.88 lower** (6.23 lower to 2.46 higher) | - | 849 (3 non-randomised studies) | ⨁◯◯◯ Very low^a,c,d,e^ |  |
| Anxiety assessed with: NRS Scale from: 0 to 10 | The mean anxiety was **0** | MD **1.03 lower** (2.22 lower to 0.39 lower) | - | 1039 (9 non-randomised studies) | ⨁◯◯◯ Very low^a,c,d,e^ |  |
| Quality of life assessed with: NRS Scale from: 0 to 10 | The mean quality of life was **0** | MD **0.73 higher** (1.17 lower to 2.64 higher) | - | 1387 (5 non-randomised studies) | ⨁◯◯◯ Very low^a,c,d,e^ |  |
| Pain assessed with: NRS Scale from: 0 to 10 | The mean pain was **0** | MD **1.22 lower** (1.92 lower to 0.52 lower) | - | 2591 (16 non-randomised studies) | ⨁◯◯◯ Very low^a,c,e^ |  |
| Insomnia assessed with: NRS Scale from: 0 to 10 | The mean insomnia was **0** | MD **1.08 lower** (2.48 lower to 0.33 higher) | - | 940 (5 non-randomised studies) | ⨁◯◯◯ Very low^a,c,e^ |  |
| ***The risk in the intervention group** (and its 95% confidence interval) is based on the assumed risk in the comparison group and the **relative effect** of the intervention (and its 95% CI).  **CI:** confidence interval; **MD:** mean difference; **OR:** odds ratio; **SMD:** standardised mean difference | | | | | | |
| **GRADE Working Group grades of evidence** **High certainty:** we are very confident that the true effect lies close to that of the estimate of the effect. **Moderate certainty:** we are moderately confident in the effect estimate: the true effect is likely to be close to the estimate of the effect, but there is a possibility that it is substantially different. **Low certainty:** our confidence in the effect estimate is limited: the true effect may be substantially different from the estimate of the effect. **Very low certainty:** we have very little confidence in the effect estimate: the true effect is likely to be substantially different from the estimate of effect. | | | | | | |

**Explanations**

a. Different THC/CBD content

b. Moderate heterogeneity

c. High heterogeneity

d. High CI range

e. Differences in population

**Table 7. Search key**

| ***Database*** | ***Search Key*** |
| --- | --- |
| ***PubMed***  ***CENTRAL Cochrane*** | (“Cancer” OR Cancer* OR “Neoplasia” OR “Neoplasm” OR Neoplas* OR “Tumor” OR “Tumors” OR “Tumour” OR “Tumours” OR “Malignancy” OR “Malignancies” OR Malignan* OR “Carcinoma” OR Carcin* OR Oncolog* OR “Adenocarcinoma” OR Adenocarcin* OR “Squamous cell carcinoma” OR “Chondrosarcoma” OR Ewing* OR “Histiocytoma” OR “Osteosarcoma” OR “Rhabdomyosarcoma” OR “Leiomyosarcoma” OR “Myxosarcoma” OR “Fibrosarcoma” OR “Fibromyxoid” OR “Myxofibrosarcoma” OR “Liposarcoma” OR “Angiosarcoma” OR “Kaposi” OR Sarcom* OR “Astrocytoma” OR “Glioma” OR “Oligodendroglioma” OR “Ependymoma” OR “Glioblastoma” OR “Medulloblastoma” OR “Neuroblastoma” OR “Blastoma” OR “Retinoblastoma” OR “cholangiocarcinoma” OR “Hepatocarcinoma” OR Hepatocarcinom* OR “Hepatocellular carcinoma” OR “Hepatocellular” carcinom* OR “Carcinoid” OR “Gastrointestinal stromal tumor” OR “Germ cell tumor” OR “Gestational trophoblastic tumor” OR “Wilms tumor” OR “nephroblastoma” OR “teratoma” OR “germinoma” OR “dysgerminoma” OR “seminoma” OR “gonadoblastoma” OR “Melanoma” OR Melanom* OR “Mesothelioma” OR Mesotheliom* OR “non small cell lung cancer” OR “small cell lung cancer” OR “Thymoma” OR leukem* OR leukaem* OR “leukemia” OR “leukaemia” OR lymphoma* OR “leucocythaemia” OR “myelodysplastic syndrome” OR “myelodysplastic syndromes” OR “myeloproliferative” OR Hodgkin*) AND (“Cannabinoids” OR “cannabis” OR cannabi* OR “phytocannabinoid” OR “sativa” OR “indica” OR “bhang” OR “ganja” OR “charas” OR “hashish” OR “hemp” OR “marihuana” OR “marijuana” OR “cannabinol” OR “cannabidiol” OR “CBD” OR “cannabigerol” OR “cannabichromene” OR “cannabigerivarin” OR “cannabidivarin” OR “cannabielsoin” OR “cannabichromevarin” OR “tetrahydrocannabinol” OR tetrahydrocannabi* OR “THC” OR "9 THC" OR “9” tetrahydrocannabi* OR "delta 9 THC" OR “delta 9” tetrahydrocannabi* OR “nabilone” OR “cesamet” OR “canemes” OR “cannador” OR “dronabinol” OR “palmidrol” OR “dexanabinol” OR “levonantradol” OR “nabiximols” OR “epidiolex” OR “Marinol” OR “Sativex” OR endocannabinoid*) |
| ***Embase*** | ((‘Cancer’ OR Cancer* OR ‘Neoplasia’ OR ‘Neoplasm’ OR Neoplas* OR ‘Tumor’ OR ‘Tumors’ OR ‘Tumour’ OR ‘Tumours’ OR ‘Malignancy’ OR ‘Malignancies’ OR Malignan* OR ‘Carcinoma’ OR Carcin* OR Oncolog* OR ‘Adenocarcinoma’ OR Adenocarcin* OR ‘Squamous cell carcinoma’ OR ‘Chondrosarcoma’ OR Ewing* OR ‘Histiocytoma’ OR ‘Osteosarcoma’ OR ‘Rhabdomyosarcoma’ OR ‘Leiomyosarcoma’ OR ‘Myxosarcoma’ OR ‘Fibrosarcoma’ OR ‘Fibromyxoid’ OR ‘Myxofibrosarcoma’ OR ‘Liposarcoma’ OR ‘Angiosarcoma’ OR ‘Kaposi’ OR Sarcom* OR ‘Astrocytoma’ OR ‘Glioma’ OR ‘Oligodendroglioma’ OR ‘Ependymoma’ OR ‘Glioblastoma’ OR ‘Medulloblastoma’ OR ‘Neuroblastoma’ OR ‘Blastoma’ OR ‘Retinoblastoma’ OR ‘cholangiocarcinoma’ OR ‘Hepatocarcinoma’ OR Hepatocarcinom* OR ‘Hepatocellular carcinoma’ OR (Hepatocellular carcinom*) OR ‘Carcinoid’ OR ‘Gastrointestinal stromal tumor’ OR ‘Germ cell tumor’ OR ‘Gestational trophoblastic tumor’ OR ‘Wilms tumor’ OR ‘nephroblastoma’ OR ‘teratoma’ OR ‘germinoma’ OR ‘dysgerminoma’ OR ‘seminoma’ OR ‘gonadoblastoma’ OR ‘Melanoma’ OR Melanom* OR ‘Mesothelioma’ OR Mesotheliom* OR ‘non small cell lung cancer’ OR ‘small cell lung cancer’ OR ‘Thymoma’ OR leukem* OR leukaem* OR ‘leukemia’ OR ‘leukaemia’ OR lymphoma* OR ‘leucocythaemia’ OR ‘myelodysplastic syndrome’ OR ‘myelodysplastic syndromes’ OR ‘myeloproliferative’ OR Hodgkin*) AND (‘Cannabinoids’ OR ‘cannabis’ OR cannabi* OR ‘phytocannabinoid’ OR ‘sativa’ OR ‘indica’ OR ‘bhang’ OR ‘ganja’ OR ‘charas’ OR ‘hashish’ OR ‘hemp’ OR ‘marihuana’ OR ‘marijuana’ OR ‘cannabinol’ OR ‘cannabidiol’ OR ‘CBD’ OR ‘cannabigerol’ OR ‘cannabichromene’ OR ‘cannabigerivarin’ OR ‘cannabidivarin’ OR ‘cannabielsoin’ OR ‘cannabichromevarin’ OR ‘tetrahydrocannabinol’ OR tetrahydrocannabi* OR ‘THC’ OR ‘9 THC’ OR (9 tetrahydrocannabi*) OR ‘delta 9 THC’ OR (delta 9 tetrahydrocannabi*) OR ‘nabilone’ OR ‘cesamet’ OR ‘canemes’ OR ‘cannador’ OR ‘dronabinol’ OR ‘palmidrol’ OR ‘dexanabinol’ OR ‘levonantradol’ OR ‘nabiximols’ OR ‘epidiolex’ OR ‘marinol’ OR ‘sativex’ OR endocannabinoid*)):ab,kw,ti |
